# Photoactivation of titanium-oxo cluster [Ti_6_O_6_(OR)_6_(O_2_C^*t*^Bu)_6_]: mechanism, photoactivated structures, and onward reactivity with O_2_ to a peroxide complex†

**DOI:** 10.1039/d2sc05671b

**Published:** 2022-12-07

**Authors:** Stephen E. Brown, Ioanna Mantaloufa, Ryan T. Andrews, Thomas J. Barnes, Martin R. Lees, Frank De Proft, Ana V. Cunha, Sebastian D. Pike

**Affiliations:** a Department of Chemistry, University of Warwick Coventry UK sebastian.pike@warwick.ac.uk; b Department of Physics, University of Warwick Coventry UK; c Research Group of General Chemistry (ALGC), Vrije Universiteit Brussel (VUB) Brussels Belgium ana.cunha@uantwerpen.be; d University of Antwerp Antwerp Belgium

## Abstract

The molecular titanium-oxo cluster [Ti_6_O_6_(O^i^Pr)_6_(O_2_C^*t*^Bu)_6_] (1) can be photoactivated by UV light, resulting in a deeply coloured mixed valent (photoreduced) Ti (iii/iv) cluster, alongside alcohol and ketone (photooxidised) organic products. Mechanistic studies indicate that a two-electron (not free-radical) mechanism occurs in this process, which utilises the cluster structure to facilitate multielectron reactions. The photoreduced products [Ti_6_O_6_(O^i^Pr)_4_(O_2_C^*t*^Bu)_6_(sol)_2_], sol = ^i^PrOH (2) or pyridine (3), can be isolated in good yield and are structurally characterized, each with two, uniquely arranged, antiferromagnetically coupled d-electrons. 2 and 3 undergo onward oxidation under air, with 3 cleanly transforming into peroxide complex, [Ti_6_O_6_(O^i^Pr)_4_(O_2_C^*t*^Bu)_6_(py)(O_2_)] (5). 5 reacts with isopropanol to regenerate the initial cluster (1) completing a closed cycle, and suggesting opportunities for the deployment of these easily made and tuneable clusters for sustainable photocatalytic processes using air and light. The redox reactivity described here is only possible in a cluster with multiple Ti sites, which can perform multi-electron processes and can adjust its shape to accommodate changes in electron density.

## Abstract 2

The molecular titanium-oxo cluster [Ti_6_O_6_(O^i^Pr)_6_(O_2_C^*t*^Bu)_6_] can be photoactivated by UV light *via* a 2-electron pathway, which avoids free radicals, resulting in a deeply coloured mixed valent (photoreduced) Ti(iii/iv) cluster that can react with O_2_ to form a peroxide complex.

## Graphical abstract

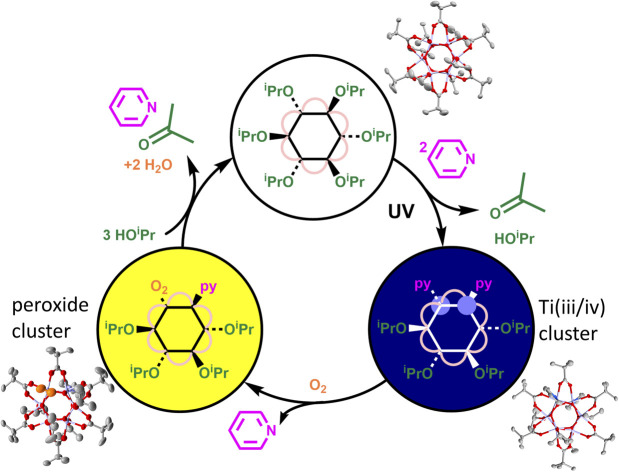

## Introduction

Titanium-oxo clusters are fascinating molecules which typically exhibit similar properties to bulk/nano TiO_2_ materials,^1,2^ and can act as atomically defined models for bulk materials, catalytic sites and dye-sensitised materials.^3–6^ Previous research has uncovered a wide variety of cluster structures and discovered many practical uses for these molecules, including as precursors to (nano)materials and metal–organic-frameworks (MOFs),^7–10^ or for use in dye-sensitised materials,^5^ gas storage materials,^11^ electron transport layers^12^ and as photocatalysts.^13^ They may also resemble the building units in Ti-MOFs.^14–16^ The ability for Ti-oxo clusters to absorb light, typically in the UV range, and to form photoexcited clusters capable of mediating redox transformations provides opportunity for their use in photocatalysis. Their well-defined structures also allows for detailed mechanistic study of these processes, providing relevant information for the reactivity of their bulk TiO_2_ counterparts.^17^ The collection of several titanium centres in a cluster enables multielectron processes which are unlikely for an isolated Ti centre.

TiO_2_ is a very important, earth-abundant, semiconductor material used in a wide range of applications. The band gap of TiO_2_ is 3–3.2 eV, depending on its structure, which only allows utilisation of UV photons for photoredox processes; therefore, a great amount of work has been invested in tuning the band gap by introducing transition metal or non-metal (*e.g.* N, S) dopants.^18^ Alternatively reduction of TiO_2_ to form ‘coloured TiO_2_’ is also of significant interest.^19^ In coloured TiO_*x*_ materials the reduction of Ti(iv) to Ti(iii) results in a material which can absorb visible light by virtue of Ti (3d) based electrons, and that can be used for photochemical production of hydrogen.^18–20^ This topic has been recently reviewed,^20^ and studied in related wide band gap oxides,^21^ however, it is noted that the structural and electronic nature of catalytic sites in these coloured TiO_2_ materials is elusive. Whilst elegant work investigating nanoparticles of TiO_2_ provides valuable understanding,^18,22–26^ by studying molecular Ti-oxo clusters, detailed insight into electronic structure and atomically resolved surface reactivity can be uncovered.^16,17^

In a previous study, Pike *et al.* discovered that Ti-oxo-alkoxide-phosphinate clusters with a heterocubane Ti_4_O_4_ core undergo a photoredox reaction in which a mixed valent cluster with a Ti(iv)_2_Ti(iii)_2_O_4_ core is formed alongside generation of ketone and alcohol organic products.^17^ These results imply that charge separation in Ti-oxo clusters can occur in even the smallest clusters, consistent with findings supporting the formation of highly localized charge-transfer excitons in bulk metal oxide semiconductors.^27^ In the [Ti_4_O_4_(O^i^Pr)_4_(O_2_PR_2_)_4_] clusters UV light induces an oxygen to metal charge-transfer process which results in the homolytic cleavage of a Ti–O^i^Pr bond. The unstable ·O^i^Pr radical formed by this process rapidly transfers an electron to the cluster core as well as transferring a proton to an adjacent alkoxide. The result of this is the formation of one acetone, one alcohol and a two-electron reduced cluster. Mechanistic studies suggested that a concerted intramolecular 2-electron process occurs. Such a process is consistent with the concept of ‘current doubling’ observed upon using TiO_2_ photoelectrodes, in which a two-electron process is triggered by a single photon absorption.^23,28^ This previous study reported that mixed valent Ti-oxo clusters underwent reaction with O_2_ to form superoxide species.^17^

In this report a simple Ti-oxo-alkoxide-carboxylate cluster is photoactivated to give well-defined mixed valence compounds with deep blue or purple colours, which can be simply prepared with good yields. Whilst several Ti-oxo cluster systems are known to undergo a colour change under UV-light,^29–32^ structural characterization and isolation of the photoexcited state remains very rare.^17^ The strong colours induced by photochemical activation makes these clusters interesting prospects for photochromic devices, including smart windows and re-writable paper.^18,29^ The photoactivated mixed-valence compounds are readily oxidised with oxygen, with one forming a structurally characterized peroxide cluster. Subsequently, by addition of alcohol to the peroxide complex, the cluster can be returned to its original state, demonstrating a closed cycle for the oxidation of alcohols using UV light and air. The oxidation of alcohols is performed industrially on a very large scale, and whilst traditional methods involve expensive and/or toxic stoichiometric oxidants, current research focusses on the use of sustainable methodologies to drive this process.^33–36^ Therefore, the catalytic competency of these low-cost, earth-abundant and organic-soluble clusters for photocatalytic alcohol oxidation under air is explored.

## Results and discussion

**Scheme 1:**
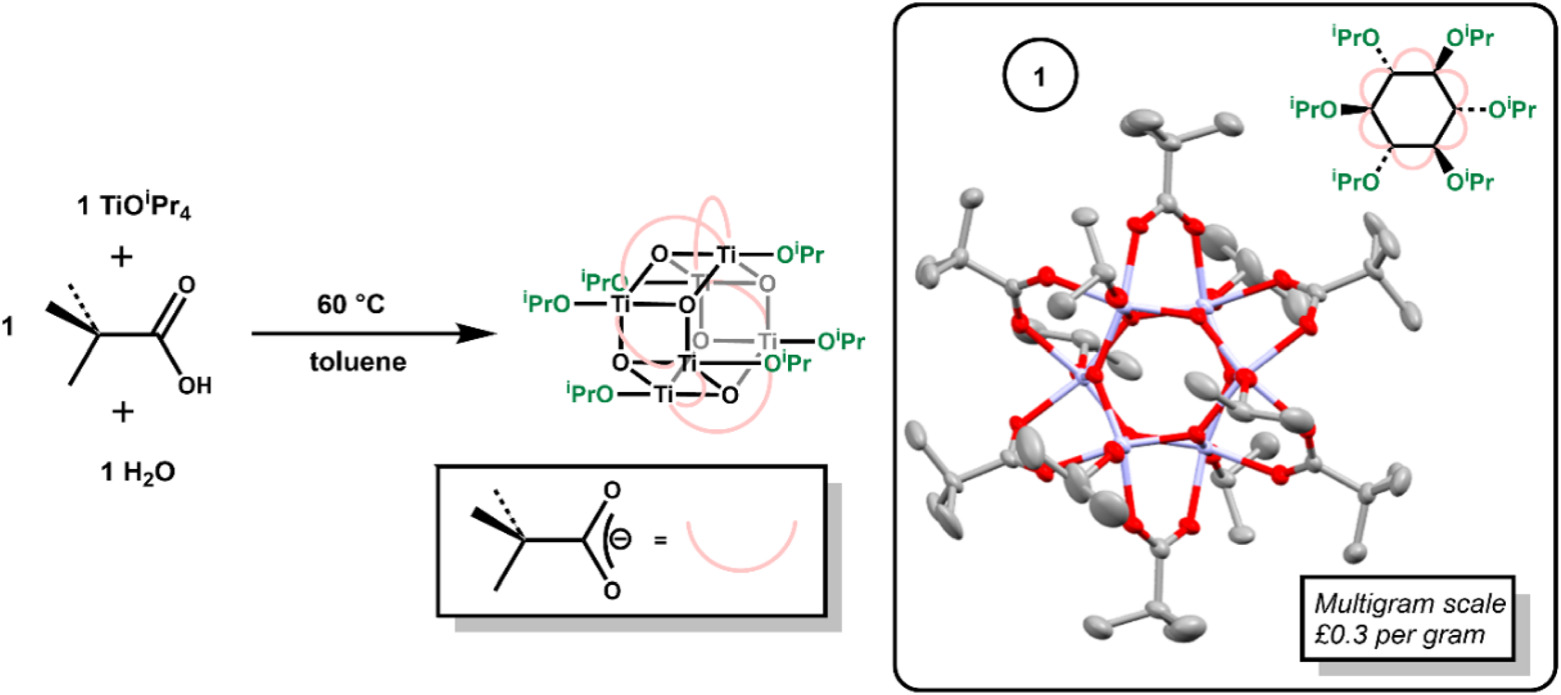
Synthesis of 1, with the solid-state structure of 1 (ellipsoids shown at 50% probability, hydrogens omitted for clarity).

Hexagonal Ti_6_-oxo clusters with formula [Ti_6_O_6_(OR)_6_(carboxylate)_6_] are well known, with examples extending to include photoactive carboxylate ligands.^6,10,11,30,37–40^ The simple Ti-oxo cluster [Ti_6_O_6_(O^i^Pr)_6_(O_2_C^*t*^Bu)_6_], 1, previously reported by Piszczek *et al.*,^37^ was prepared *via* the reaction of [Ti(O^i^Pr)_4_] with pivalic acid and water (Scheme 1). Mixing [Ti(O^i^Pr)_4_] and pivalic acid first forms the (previously reported)^39^ dimeric complex [Ti_2_(O^i^Pr)_6_(O_2_CCMe_3_)_2_(HO^i^Pr)] (Fig. S1†). To this, one equivalent of water was added, and the solution heated to 60 °C overnight to produce crystalline 1 on the multigram scale (Fig. S2–S6†). Considering the low cost of the starting materials (both [Ti(O^i^Pr)_4_] and pivalic acid cost ∼£0.1 per g) 1 is very cheap to produce, with an ingredient cost of approximately £0.3 per gram of product.

Compound 1 crystallises from toluene as a mixture of two polymorphs (Fig. S3–S5†); one of which includes a toluene solvent molecule. A third polymorph was also previously reported.^37^ Both new polymorphs were characterized by single crystal X-ray diffraction, with identical cluster geometries (Scheme 1, Fig. S3 and S4†). The structure comprises a hexagonal prismatic Ti_6_O_6_ core, with shorter Ti–O bonds around the hexagonal ring (1.879(2)–1.928(2) Å) than between the two rings (2.140(2)–2.161(2) Å). The edges of the cluster are supported by pivalate ligands, whilst the two hexagonal faces are appended with three isopropoxide units.

Compound 1 was studied in solution by UV spectroscopy, revealing an absorption onset of 3.52 ± 0.03 eV (352 nm) (Fig. S7 and S8†), similar to that observed for [Ti_4_O_4_(O^i^Pr)_4_(O_2_PR_2_)_4_] (R = Cy or Ph) and other Ti-oxo clusters without conjugated ligands.^17,41^ The HOMO–LUMO gap is larger than that seen in the band-gap of bulk TiO_2_, consistent with quantum confinement concepts – with the gap formed between discreet molecular orbitals, in contrast to the extended band structure in TiO_2_.^17^ TD-DFT calculations indicate the lowest energy electronic absorptions in 1 are oxygen to metal charge transfer, without significant contribution from the carboxylate ligand (Fig. S9 and S10,† >50% donor orbital density on the oxo units). The lowest excited states are calculated at 365 nm, with stronger absorptions at 357 nm (excited states 11 and 12, Fig. S9†). The natural transition orbitals (Fig. S10†) show that an electron is transferred from the oxygen lone-pairs of the [Ti_6_O_6_] moiety to the empty d-orbitals centered at the Ti-atoms.

**Fig. 1:**
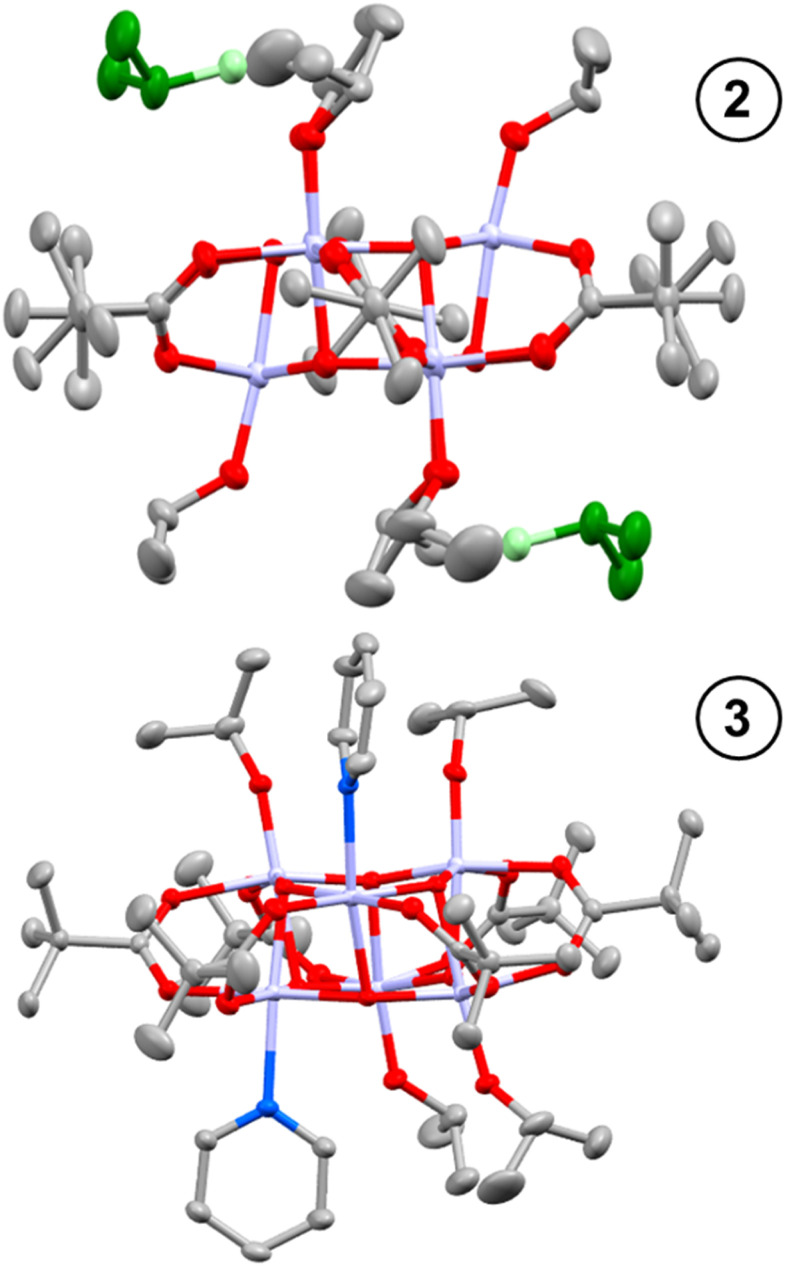
Solid-state structures of 2 (top) and 3 (below), hydrogen atoms omitted for clarity. Ellipsoids displayed at 50% probability. Ti – pale purple; N – blue; C – grey; O – red. In 2, H-bonded ^i^PrOH solvent molecules are depicted in green.

**Scheme 2:**
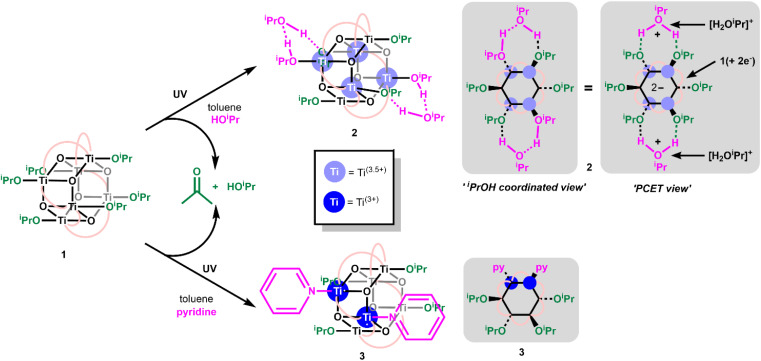
Photoreaction of 1 in the presence of coordinating solvents to form 2 or 3 alongside acetone and isopropanol.

Toluene solutions of 1 (under inert atmosphere) undergo photoredox reactivity under medium-wave UV light (∼302 nm, 3 mW cm^−2^ @ 1 cm, Analytik Jena UVLM-26 EL series UV lamp) generating dark blue solutions (Fig. S11–S14†).^42^ In previous studies of the photoredox reaction of tetrameric [Ti_4_O_4_(O^i^Pr)_4_(O_2_PR_2_)_4_] the photoreaction was promoted by the presence of coordinating solvents, with no photoreaction in their absence.^17^ The coordinating solvents are also suggested to bind to the resultant low-coordinate Ti(iii) sites. To study this influence, 1 was dissolved in toluene and irradiated in the absence of additives, and also in the presence of 30 equivalents of coordinating solvents (pyridine, THF, ^i^PrOH). In contrast to previous findings,^17^1 readily undergoes a photoredox reaction in the absence of coordinating species (approx. 47% reaction after 30 minutes under 302 nm lamp, Fig. S11†), however, the reaction occurs more rapidly in the presence of coordinating solvents (*i.e.* reaction is complete within 30 minutes with 30 equiv ^i^PrOH, Fig. S12–S14†). In most cases ^1^H NMR spectroscopy data was consistent with the formation of an equal amount of acetone and isopropanol (Fig. S15†), as expected from the photoreaction mechanism suggested for similar clusters.^17^ An exception was observed when no additive is included, in which case only acetone is observed (Fig. S11†), it is likely any ^i^PrOH formed remains coordinated to the photoproduct under these conditions and is not observed as free alcohol. The NMR spectra also show the formation of a cluster photoproduct during the photoreactions (Fig. S16–S22†). The solutions become deeply coloured on photoreaction, turning dark purple with pyridine or dark blue with THF or ^i^PrOH (Fig. S23–S26†). Excitingly, the coloured solutions slowly formed crystals or precipitates of dark-coloured compounds. The photoproducts with ^i^PrOH (2) and with pyridine (3) gave crystals suitable for X-ray diffraction (Fig. 1), allowing for structural characterisation. In both cases two isopropoxide ligands have been lost from 1 (as isopropanol and the photooxidation product acetone), and the photoreduced cluster has a formula of [Ti_6_O_6_(O^i^Pr)_4_(sol)_2_(O_2_CCMe_3_)_6_] (sol = ^i^PrOH, 2; or pyridine, 3) (Scheme 2). In 3 bond-valence sum calculations clearly indicate that the Ti atoms coordinated to pyridine are Ti(iii), with the other four Ti as Ti(iv) (Tables S1–S3†) with Ti–O^i^Pr bond lengths very similar to those in 1 (1, 1.773(2)–1.785(2) Å; 3, 1.792(2)–1.806(2) Å) (Fig. S27 and Table S4†). In 2, the situation is more complex, two Ti atoms are clearly Ti(iv) with Ti–O^i^Pr bond lengths of 1.801(3) Å, but the other four Ti atoms exhibit elongated bond lengths to the coordinated O^i^Pr group (1.941(3) Å and 1.953(3) Å) (Fig. S27†). Bond-valence sum calculations for these Ti sites give a value consistent with an oxidation state of 3.5 (Table S2†), indicating that two d-electrons are delocalized over these four Ti sites. A disordered molecule of isopropanol solvent crystallises in the space between the two elongated Ti–O^i^Pr groups (Fig. 1 and S27†), which suggests a total of two protons are shared between the two coordinated O^i^Pr groups and the one solvent molecule (forming a Ti(3.5)-OR-H-OR-H-OR-Ti(3.5) structure (see Scheme 2), consistent with the expected valence of the molecule. From an alternative viewpoint, 2 can be considered as a molecule of 1 which has gained two electrons and two protonated isopropanols (*i.e.*, 2 = 1(+2e^−^)·2[H_2_O^i^Pr]^+^, Scheme 2), fully consistent with recent reports of proton-coupled electron-transfer (PCET) observed in metal-oxo clusters, TiO_2_ nanoparticles and in Ti-MOFs.^15,22,25,26,43^ This mixed valence structure is also consistent with the previously described photoreduced Ti cluster [Ti_4_O_4_(O_2_PPh_2_)_6_] which also shows four Ti atoms with a ‘3.5’ oxidation state.^17^ The structure of 2 exhibits *C*_i_ symmetry, and shows minor distortions compared to 1 (Table S4†). The structure of 3, with two coordinated pyridine groups, is less symmetrical than 1 or 2 and exhibits a wider range of bond lengths and angles (Table S4†).

**Fig. 2:**
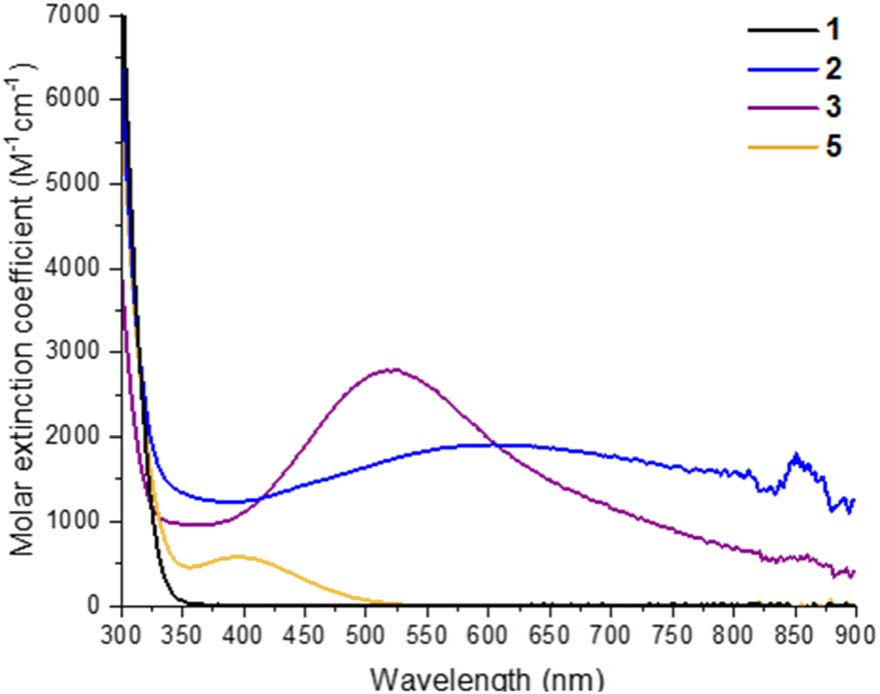
UV/visible absorption spectra of 1, 2, 3 and 5 recorded at 0.29 mM in toluene. Spectrum of 5 prepared by adding dry air to a solution of 3.

2 and 3 can be straightforwardly prepared as dark blue and dark purple (both almost black) crystalline solids (Fig. S26†) in good, isolated yield (2, 73%; 3, 63%) by exposure of 1 in toluene + isopropanol/pyridine to 302 nm light for several hours. This makes them an attractive prospect as well-defined highly reducing compounds containing either two high energy electrons (3) or two high energy electrons + two protons (2), which could have a variety of uses in synthesis or for electron transfer studies.^24^ Furthermore, the deep colouration of these compounds, caused by intervalence charge transfer and d–d excitations,^17,44^ makes for a strong photochromic effect in contrast to colourless 1 (Fig. 2). The molar extinction coefficient of 2 is above 1 × 10^3^ M^−1^ cm^−1^ across the visible region and approaches 2 × 10^3^ M^−1^ cm^−1^ at its maximum of 608 nm (Fig. 2, S23†). 3 has a narrower absorption peak, with a maximum at 523 nm with a molar extinction coefficient of 2.8 × 10^3^ M^−1^ cm^−1^ (Fig. 2, S24†). The isolated crystals of 2 and 3 were redissolved in d_8_-toluene and gave identical spectra to the products observed by *in situ*^1^H NMR spectroscopy during the photochemical reaction of 1 (Fig. S19–S22†). 1D and 2D ^1^H NMR spectra of 3 clearly show two sets of O^i^Pr environments are observed, in line with the symmetry of 3 in the solid state, and a single environment for coordinated pyridine is also resolved (Fig. S21†). The ^1^H NMR spectrum of 2, is very similar to that of 1, with only slight shifts in O^i^Pr and pivalate resonances, and shows only a single, averaged, site for six O^i^Pr groups on the cluster plus two equivalents of free ^i^PrOH, as is expected considering the crystal structure (Fig. S19†). A broad protic resonance at 8.4 ppm displays a relative integral value of four, suggesting that the four alcohol protons associated with the cluster are in dynamic exchange. At low temperature (223 K) the Ti–O^i^Pr signal begins to split into a 4 : 2 ratio, suggesting localization of the protons within the structure (Fig. S20†). The NMR spectra for 1 photoactivated in the presence of THF, was consistent with a structure of similar symmetry to 3, *i.e.* [Ti_6_O_6_(O^i^Pr)_4_(THF)_2_(O_2_CCMe_3_)_6_] (4), with both THFs on adjacent Ti positions (Fig. S22†). The proton signals at the 1,4-positions of coordinated THF display as a pair of roofed multiplets, suggesting a relatively fixed geometry, and differing environments on each face of the THF unit when coordinated to the cluster (see Fig. S27† for the related structure of 3). Diffraction quality crystals of 4 were not able to be isolated.

EPR spectroscopy and the Evans NMR method confirm that 2 and 3 do not contain unpaired electrons, consistent with their sharp NMR spectra (Fig. S28†). It is noteworthy that a minor paramagnetic signal (∼1% of sample) associated with a Ti-containing by-product species was observed by EPR spectroscopy and SQUID magnetometry in some batches of 2 (Fig. S28–S32†). When a solution of 2 is irradiated with 302 nm the signal of the paramagnetic by-product grows (monitored by EPR spectroscopy, Fig. S29–S31†), and a trace of acetone and extra isopropanol is observed by ^1^H NMR spectroscopy, hinting at a further photoprocess involving the remaining alkoxides on 2.

To further probe the electronic structure of 2 and 3, DFT calculations were performed. For 3, three different solutions were obtained, which describe 3 with either a closed-shell (diamagnetic), broken symmetry (diradical diamagnetic) or open-shell triplet (paramagnetic) structure. The singlet, closed-shell solution was found to be highest in energy (+0.42 or +0.05 eV, using B3LYP/TZ2P or TPSSh/TZ2P//PBE/TZ2P respectively), while the triplet and broken symmetry solutions were found to be near-degenerate.^45^ The spin density (Fig. S33b†) indicates that the radical centers are localized on the Ti atoms that are connected to the pyridine ligands, in line with the observations based on the X-ray structure. Calculated hyperfine coupling constants indicate strong coupling of the unpaired electrons with the Ti-centers. For 2, similar instabilities in the Kohn–Sham wavefunction were found as for 3, indicating that the electronic structure of 2 is similar to that of 3, however, 2 is expected to be more dynamical than 3 due to the fact that the different configurations of 2 can easily interconvert into each other by proton transfer. The closed-shell solutions are higher in energy (+0.99 eV or +0.27 eV, using B3LYP/TZ2P or TPSSh/TZ2P//PBE/TZ2P respectively) than the open-shell singlet and triplet states, which are degenerate. The spin density in the open-shell singlet case (Fig. S33a†) is localized on the Ti atoms that are connected to the protonated HO^i^Pr groups. Reported computational analysis of related tetrameric mixed-valent Ti_4_O_4_(O_2_PPh_2_)_6_ found a triplet state to be the most stable, but open-shell diradical and closed shell singlet states were located very close in energy (Δ*E* = +0.06 and +0.26 eV respectively, using B3LYP).^17^ Similarly to 2 and 3, when Ti_4_O_4_(O_2_PPh_2_)_6_ is isolated as a solid compound, it does not exhibit an EPR signal (solid-state, 100 K). Considering the near degeneracy of the different solutions for 2, 3 and Ti_4_O_4_(O_2_PPh_2_)_6_, environment (solvent) effects could shift the energy ordering, resulting in different ground states in different environments. Combining the experimental and computational analysis, 2 may be best described as an open-shell ‘diradical’ singlet, with two antiferromagnetically coupled but spatially separated d-electrons (3.6 Å apart, Fig. S33a†), although a closed-shell singlet ground state description is also plausible. For 3 both descriptions are possible. For both compounds calculations do not predict any significant metal–metal bonding interaction between the singly occupied d-orbitals (ESI note 1†). 2 rapidly reacts with pyridine in solution to form 3, demonstrating that the electronic structure will easily adjust upon changing the coordination environment around the cluster.

**Scheme 3:**
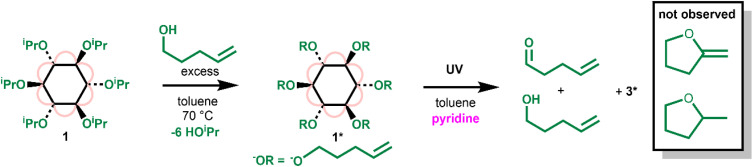
Preparation of 1* and its photoredox reaction under UV light.

To gain a greater understanding of the photoactivation of 1, the related compound [Ti_6_O_6_(O(CH_2_)_3_CH=CH_2_)_6_(O_2_C^*t*^Bu)_6_] (1*) was prepared by an alkoxide exchange process by heating 1 with an excess of 4-pentene-1-ol (Scheme 3 and Fig. S34†). 4-Pentene-1-oxyl radicals are known to undergo a rapid irreversible 5-*exo*-trig ring-closure to produce the carbon based radical of 2-methyltetrahydrofuran (with a reported rate of cyclisation of k^5−*exo*^ = (4 ± 2) × 10^8^ s^−1^ (30 °C)).^46^ However, upon irradiating 1* with UV light (in the presence of pyridine) the organic products identified by ^1^H NMR spectroscopy were exclusively 4-pentene-1-ol and pent-4-enal, the expected alcohol and aldehyde products following a concerted two-electron photoredox process (Scheme 3, Fig. S35 and S36†). No new signals in the 3–4.2 ppm region which would be consistent with a cyclic ether product were observed. This result strongly implies that photoreactivity does not occur *via* a free-radical pathway, which suggests that 1 should give enhanced selectivity in oxidation processes.

**Scheme 4:**
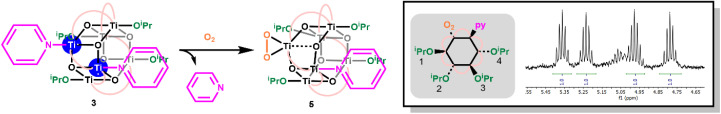
Reaction of 3 with O_2_. Insert shows symmetry of the cluster when viewed from above hexagonal plane, and ^1^H NMR Ti–O^i^Pr resonances in 5, N.B. minor additional O^i^Pr heptet signal is a trace of 1.

**Fig. 3:**
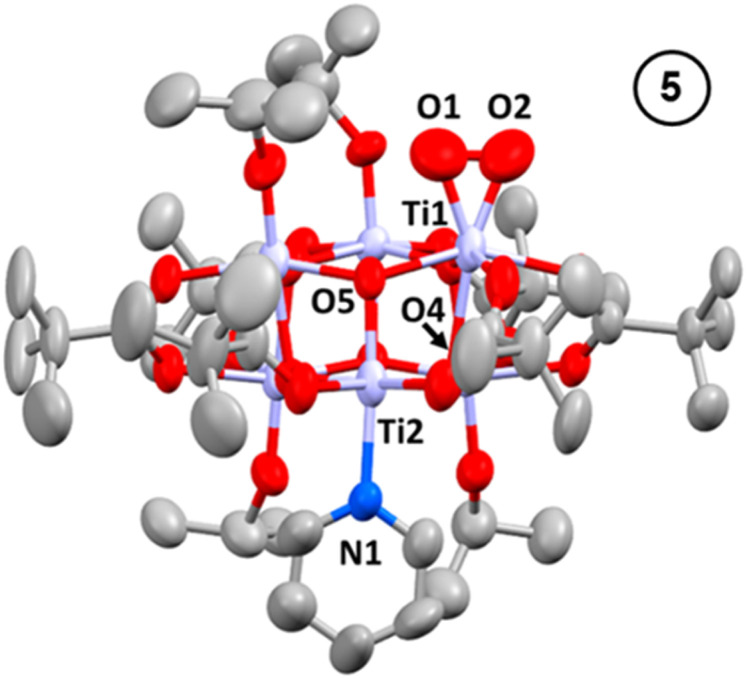
Solid-state structures of 5, hydrogen atoms and solvent molecule omitted for clarity. Ellipsoids displayed at 50% probability. Selected bond lengths (Å) and angles (°): Ti1–O1, 1.842(9); Ti1–O2, 1.831(9); O1–O2, 1.275(11); Ti1–O4, 2.551(6); Ti2–O5, 1.926(5); Ti2–N1, 2.166(5); O1–Ti1–O2, 40.6(3).

Oxidation of 3, either as a powder or dissolved in toluene, using dry air results in a colour change to yellow. In solution, the reaction occurs rapidly, and is complete within 90 seconds. Solution NMR spectra of this yellow species reveals loss of one pyridine ligand, but retention of the other. Four independent O^i^Pr signals are now resolved, and no further proton signals are observed, indicating a cluster with formula [Ti_6_O_6_(O^i^Pr)_4_(py)(O_2_)(O_2_CCMe_3_)_6_] (5) (Scheme 4 and Fig. S37†). 5 was crystallised from CH_2_Cl_2_ and single crystal X-ray diffraction confirms this structure, showing a side-on binding mode of the peroxide unit to a single Ti site (Fig. 3). Formally, the two Ti 3d electrons have now been transferred to the O_2_ unit, resulting in all Ti atoms in the +4 oxidation state, which is confirmed by bond-valence sum calculations (Table S5†). The formally zwitterionic nature of 5 is compensated by distorted bond lengths in the structure – with the formally negatively charged Ti–(O_2_) unit, displaying longer bonds to the surrounding O atoms and the formally positive Ti–py unit showing shorter bonds to surrounding O (*e.g.* the Ti–O bond connecting the hexagonal faces is drastically different at these positions; Ti1–O4, 2.551(6) Å; Ti2–O5, 1.926(5) Å), this suggests the charges are distributed across the cluster, and the multinuclear cluster structure is important for accommodating this change of electron density. Several examples of Ti–peroxide complexes are known, with the majority showing the same side-on binding mode.^47–51^ The O–O bond length in 5 is determined to be 1.275(11) Å from the X-ray data, although minor disorder in the structure may influence this measurement (ESI note 2†). The measured bond length is longer than in O_2_ (1.21 Å) but shorter than for H_2_O_2_ (1.48 Å) and other reported Ti-peroxo compounds. EPR spectroscopy of solid 5 shows no signals, consistent with a diamagnetic peroxide species and ruling out the possibility of a superoxide complex. DFT calculations on 5 show a closed-shell ground state, with the O_2_ π* orbitals doubly occupied. The optimized O–O bond length is calculated to be 1.458 Å, much closer to the O–O bond length in H_2_O_2_ and other Ti–O_2_ complexes.^47–51^ The calculated Ti–N bond length is also considerably longer than the one found in the X-ray data: 2.353 Å *vs.* 2.166(5) Å (measured), but all other bond lengths are in good agreement with the X-ray data. Peroxide coordination is in contrast to the previously reported oxidation of mixed-valent [Ti_4_O_4_(O_2_PPh_2_)_6_] which, instead, formed the superoxide salt [Ti_4_O_4_(O_2_PPh_2_)_6_][O_2_]_2_.^17^ In [Ti_4_O_4_(O_2_PPh_2_)_6_] the Ti sites are coordinatively saturated by strongly bound chelating ligands, which contrasts to 2 or 3 which have a coordinatively labile solvent molecule, this may cause electron transfer to O_2_ to occur *via* different mechanisms, perhaps explaining the different oxidation products. The IR spectrum of 1,^37^2, 3 and 5 are very similar (Fig. S38–S42†), however, two new signals are observed for 5 at 892 and 805 cm^−1^. Previously characterized Ti–(O_2_) peroxo complexes report O–O stretching frequencies in the range of 856–950 cm^−1^ and, by DFT, the O–O stretching frequency in 5 is predicted to be 943 cm^−1^,^44,47–51^ therefore, we tentatively attribute the signal at 892 cm^−1^ as the peroxide O–O stretch. The UV/vis spectrum of 5 shows a new peak with a maximum at 395 nm which extends into the visible region (Fig. 2, S23–S25†). Calculations imply the absorption onset occurs at 1.81 eV (683 nm), corresponding to an excitation from the O_2_ π* orbitals to the mixed Ti(d) and π* orbital of the pyridine ligand (Fig. S43 and S44†). Stronger absorptions are found at 3.29 eV (377 nm), mainly corresponding to a ligand to metal charge-transfer, O_2_(π*) → Ti(d), excitation (Fig. S44†) consistent with other reported Ti-peroxo complexes.^51^

**Fig. 4:**
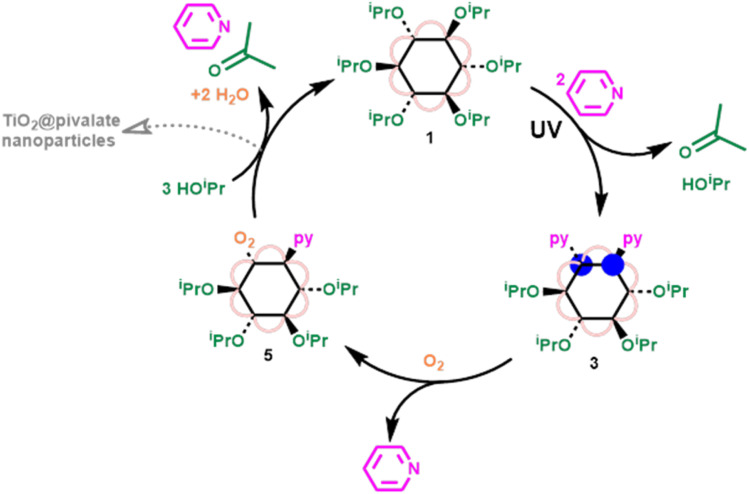
Closed cycle, showing photoreduction of 1 in the presence of pyridine to form 3, oxidation of 3 by O_2_ to form 5, and reformation of 1*via* reaction of 5 with excess ^i^PrOH.

Adding excess ^i^PrOH to 5 results in the relatively fast reformation of 1 (using ∼25 equivalents of ^i^PrOH, ∼47% conversion to 1 had occurred within 10 minutes), with acetone produced as an oxidized byproduct, completing a closed cycle (Fig. 4 and S45†). Conversion of 5 to 1 requires reaction with three isopropanol molecules; one becoming oxidized by the peroxide unit to give acetone and water (see ESI note 3†), a second protonating the cluster and displacing the neutral pyridine ligand with an alkoxide. After these steps the resulting cluster would be a hydroxide compound [Ti_6_O_6_(O^i^Pr)_5_(OH)(O_2_CCMe_3_)_6_], and the third alcohol could initiate OH/OR exchange to regenerate 1. The order of these three steps is not clear, as NMR spectroscopic analysis only shows direct formation of 1, without observation of intermediates. However, the formation of a hydroxide intermediate, suggests that condensation of Ti–OH units is possible, resulting in larger clusters or macromolecular agglomerates as a side reaction (Fig. 4), as has been previously reported during the hydrolysis of acetate capped [Ti_6_O_6_(O^i^Pr)_6_(O_2_CMe)_6_].^10,52,53^ During the reaction of 5 with ^i^PrOH, 1 is the only cluster product observed by solution NMR spectroscopy, however, some precipitate also forms, with ∼30% of the expected NMR signal lost by analysis of integrals *versus* an internal standard, which is attributed to condensation reactions by Ti–OH intermediates.

Oxidation of 2 by dry air induces a colour change to yellow, similar to the oxidation of 3, however, the presence of four ^i^PrOH units per cluster in 2 allows the reaction to proceed slowly all the way through to 1, which is observed by NMR, UV/vis and IR spectroscopy (Fig. S23, S39 and S46†). Immediately after 2 is oxidized by air, at least three minor alkoxide signals are observed by ^1^H NMR spectroscopy, which may correspond to a peroxide complex similar to 5, however, the major signals observed are from 1 (Fig. S46†). After standing overnight the yield of 1 is ∼66%, presumably with hydrolysis of the remaining clusters giving insoluble pivalate capped Ti-oxide nanostructures (N.B. some additional isopropanol, formed by hydrolysis, is also observed by NMR spectroscopy).^8,10,52,53^ A small quantity of acetone is formed, although less than would be expected, suggesting other side reactions or absorption of some acetone onto any nanoparticle surfaces.^54^ An unassigned minor signal at 1.3 ppm is also observed, which could be from a small quantity of dissociated pivalic acid.

The moisture instability of the system hinders its use as a robust well-defined photocatalyst, however, to investigate catalytic potential, 1 was tested for the photocatalytic oxidation of alcohols using only air as the oxidant and 302 nm irradiation (∼3 mW cm^−2^). Using isopropanol as the reagent, at a 0.1 mol% catalyst loading, at least 16 or 28 equivalents of acetone per cluster were produced over 4.5 or 11.5 hours respectively (monitored by NMR spectroscopy, see ESI note 4, Fig. S47†). Control reactions using no catalyst or using TiO_2_ powder (with an equivalent Ti content), resulted in no conversion or much slower production of acetone (∼0.15 of rate of cluster catalyst) respectively. 1 readily undergoes alkoxide exchange with free alcohols (ROH), hence, 1 can convert to [Ti_6_O_6_(*OR*)_6_(O_2_CCMe_3_)_6_] in order to facilitate oxidation of an added alcohol *via* the same structure. Therefore, the photooxidation of 1-butanol and 1-octanol selectively to linear aldehydes was demonstrated using 1 as the initial photocatalyst (with approximately 16 and 13 equivalents of aldehyde produced per cluster within 4.5 hours irradiation respectively). The ^1^H NMR signals of the cluster are lost during catalysis after 3–5 hours, and when isopropanol is the reagent a precipitate begins to form. It is anticipated that this is a result of condensation of clusters into macromolecules or nanoparticles (which are insoluble with O^i^Pr ligands but remain solubilised with O^*n*^Bu or O^*n*^Oct).^10,52,53^ Interestingly, the catalytic reaction continues even after loss of well-defined cluster, at a slightly slower rate, suggesting that the larger less-defined species formed continue catalysing the reaction. Based on previous studies of Ti_6_-oxo cluster hydrolysis, the macromolecular agglomerates may retain the hexagonal Ti_3_O_3_ face capped by three alkoxides, allowing for a similar photoredox mechanism to occur in the nanostructures.^10,52,53^

These photocatalytic experiments represent a proof-of-principle and suggest that photooxidation reactions can be achieved using well-defined Ti-oxo clusters and related nanostructures. Advantages may include enhanced selectivity (avoiding free-radicals) and substrate scope (clusters retain a hydrophobic surface) compared to other (photo)catalysts for aerobic alcohol oxidation. Whilst a brighter light source should increase reaction rate, it will be crucial to develop clusters with greater stability to onward hydrolysis and condensation, which could be achieved through steric protection of the alkoxide sites, the use of larger Ti-oxo clusters^55^ or by embedding the cluster structures into a rigid framework material.^14^

## Conclusions

The simple titanium oxo cluster 1, undergoes photoredox reactivity under UV light, with a drastic colourless to dark blue/purple colour change, and allows for detailed structural analysis of the resulting mixed valence Ti(iii)/Ti(iv) clusters. These can be considered molecular analogues of reduced ‘black TiO_2_’. Interaction with coordinating solvents is shown to dictate the physical and electronic structure of these reduced species. The photoredox reaction results in the oxidation of an alkoxide group to generate an aldehyde or ketone. The photoreduced clusters are capable of reaction with O_2_ to give a peroxide complex; a strongly oxidising species capable of further oxidation reactions. Therefore, oxidation of alcohols can be achieved photocatalytically using 1, although the catalyst slowly transforms into TiO_2_ nanostructures in the presence of the moisture by-product.

Perhaps one of the most remarkable and noteworthy discoveries in this study is the ability of the Ti_6_O_6_ cluster to adjust and modify its shape to accommodate changing electronic structure and charge distribution, smoothing the way for multi-electron redox reactions to occur. By allowing for two electrons to be injected into the Ti d-orbitals the cluster is able to undergo 2-electron (non-radical) reactivity which would not be possible for a complex with a single Ti atom. In this way the cluster behaves more like a late transition-metal complex, enabling selective (two-electron) bond breaking/forming processes and avoiding less selective free-radical based processes. In the formation of 2, both electrons and protons are simultaneously added to 1, fully consistent with expected proton-coupled electron-transfer observed in the photoreduction of TiO_2_ nanoparticles. Furthermore, reaction of photoreduced 3 with O_2_ gives a distorted, formally zwitterionic, peroxide cluster, again indicating the importance of the whole cluster for redistributing electron density upon reaction. These low-cost and earth abundant systems show promise for photochromic and photocatalytic applications, and their precise reactivity and highly tuneable structure highlights opportunity for further development.

## Data availability

The raw data that support the findings of this study are available from https://wrap.warwick.ac.uk/171205/.

## Author contributions

Original ideas by SDP. Synthesis, photochemistry and characterization by IM, SEB, RTA, TJB and SDP. EPR spectroscopy by SEB. Crystallography by SDP and SEB. Catalysis by SDP and TJB. Magnetometry by MRL. Computational studies by AVC and FDP. Paper written by SDP, SEB and AVC with all authors contributing to final version.

## Conflicts of interest

There are no conflicts to declare.

## Supplementary Material

SC-014-D2SC05671B-s001

SC-014-D2SC05671B-s002
