# Supplementary material for: Photoactivation of titanium-oxo cluster [Ti6O6(OR)6(O2CtBu)6]: mechanism, photoactivated structures, and onward reactivity with O2 to a peroxide complex
Source: Chem Sci. 2022 Dec 7;14(3):675–83. doi: 10.1039/d2sc05671b (PMC9847671; doi:10.1039/d2sc05671b)
Supplement: SC-014-D2SC05671B-s001 [file SC-014-D2SC05671B-s001.pdf]

Electronic Supplementary Material (ESI) for Chemical Science.
This journal is © The Royal Society of Chemistry 2022

Supporting Information

Photoactivation of titanium-oxo cluster, $[TiO(OR)(O_2C^tBu)]_6$: mechanism, photoactivated structures, and onward reactivity with O_2 to a peroxide complex.

Stephen E. Brown,[1#] Ioanna Mantaloufa,[1#] Ryan T. Andrews,[1] Thomas J. Barnes,[1] Martin R. Lees,[2] Frank De Proft,[3] Ana V. Cunha*,[3,4] Sebastian D. Pike*[1]

1. Department of Chemistry, University of Warwick, Coventry, U.K.
2. Department of Physics, University of Warwick, Coventry, U.K.
3. Vrije Universiteit Brussel, Brussels, Belgium
4. University of Antwerp, Antwerp, Belgium

# equal contribution of authors

Contents

Experimental details 2

Tauc plot method for determining absorption onset 3

Computational information 3

Preparation of compounds 4

Synthesis of 1 via $Ti_2(OR)_6(O_2CCMe_3)_2(HOR)$ 6

Figs S1-S2 6-7

Crystallography of 1 7

Figures S3-S10 8-12

In-situ NMR spectroscopy analysis of photoredox reaction of 1 14

Figures S10-S42 and Tables 14-47

Supporting note 1. Examination of any metal-metal bonding interactions 33

Supporting note 2. Crystallography of 5 46

Supporting note 3. Testing for hydrogen peroxide 46

Supporting note 4. Photocatalytic experiments 46

Crystallography Tables 47

Computational Data 48

References 103

Experimental details

All manipulations were undertaken using a nitrogen filled glovebox or using a Schlenk line, unless otherwise stated. Pivalic acid, 4-penten-1-ol, octanol and $Ti(O^iPr)_4$ were used directly from suppliers, As an air sensitive liquid, all additions of $Ti(O^iPr)_4$ were transferred by syringe within the glovebox (measured by negative weight of donor flask). 'Extra-dry' acetone and isopropanol, were purchased from Acros Organics, 'anhydrous' toluene and THF was purchased from Sigma Aldrich. Pentane, hexane and 1-butanol were purchased as standard grade. All solvents were degassed by bubbling with N_2 for 30 minutes and dried by storing over activated 4 Å molecular sieves (or 3 Å for isopropanol) under nitrogen. D^8-toluene and pyridine were dried by stirring over CaH_2 and distilled using grease-free trap-to-trap apparatus, before storing over 4 Å molecular sieves under nitrogen. Moisture analysis by Karl Fischer spectrometric analysis, toluene = 0 ppm, ^{i}PrOH = 15 ppm, pentane = 3 ppm, THF = 10 ppm. Dry air was prepared by sealing air in a 500 mL glass bomb containing activated 4Å molecular sieves and leaving overnight.

The raw data that support the findings of this study are available from http://wrap.warwick.ac.uk/171205/.

Photoirradiation was undertaken using a Analytik Jena UVLM-26 EL series UV lamp (output 302 nm, approx. 3 mW/cm^2 @ 1 cm, 6 W power) or using a Neo VU-3 DC (output 365 nm, ~14 mW/cm^2 @ 10 cm).[1] Light power was measured using a calibrated Hamamatsu S1337-66BQ Si photodiode. UV experiments were conducted in a fume-hood under a plastic cover to avoid exposure to UV light. According to the manufacturers available data we anticipate the output for long wave (355 nm) UV irradiation to have a maximum at 365 nm dropping to 10% intensity at ±15 nm (i.e. spanning at least 350-370 nm), and for the medium wave (302 nm) UV irradiation to range from 280-375 nm, with a maximum output at 302 nm. It is noteworthy that glass flasks (used throughout this study unless stated otherwise) begin to absorb photons <335 nm and will reduce the flux of any high energy photons from medium wave UV irradiation.

NMR spectra were recorded on Bruker Avance III HD 400 MHz or 300 MHz instruments and all chemical shifts reported in parts per million (ppm). Solid-state Fourier transform infrared spectra were recorded using a Agilent Cary 630 FTIR with an ATR Sampling Accessory. Solution ultraviolet spectroscopy was recorded using a Implen NanoPhotometer C40, using a bespoke Young's tap cuvette for air free analysis. All mass spectrometry measurements were performed using an Agilent 6130B single Quad (ESI) spectrometer. Elemental Analysis was determined at London Metropolitan University by Orla McCullough.

For in-situ analysis of photoreactions by NMR spectroscopy the starting material **1** (7 mg, ~5 µmol) was loaded into a Young's tap NMR tube inside a glovebox. Solvent (d^8-toluene, 0.5 mL) and 30 equiv. of additive (e.g. ^{i}PrOH, 12.5 µL) were added to the tube via syringe and micropipette respectively under a flow of N_2 using Schlenk line apparatus. The tube was sealed and then frozen in liquid nitrogen before exposure to vacuum, this cooling/vacuum proceedure was repeated once more and the headspace refilled with nitrogen to ensure an air free environment before photoirradiation.

Solution, frozen solution and powder EPR experiments were performed using a Bruker EMX spectrometer at X-band (~9.33 GHz) equipped with a liquid nitrogen cryostat, the microwave frequency was measured using an external frequency counter. Low temperature data was collected at 100 K. All EPR experiments were performed using the same cavity and the temperature was monitored using the temperature controller's thermocouple. EPR data was modelled using the EasySpin toolbox for MATLAB as a triplet with the following spin Hamiltonians:

$$H = g\beta B\cdot S + D\{S_Z^2 - S(S+1)/3\} + E(S_X^2 - S_Y^2)\ ,$$

$$H = g\beta B\cdot S + D\{S_Z^2 - S(S+1)/3\} + E(S_X^2 - S_Y^2) + SAI$$

With S=1, D and E are the axial and rhombic zero field splitting terms. A is the hyperfine splitting to ^{47}Ti (I = 5/2) and ^{49}Ti (I=7/2) at their natural abundances. Due to the lack of detail in hyperfine features for the low temperature spectra A was set as isotropic with a value of 0.00164 cm^{-1} determined from the room temperature spectrum.

In the isotropic room temperature solution spectrum, the signal was modelled with simplified spin Hamiltonian:

$$H = g\beta B\cdot S + SAI$$

With the isotropic limit of S'=1/2 and A as the hyperfine splitting to 1 Ti per spin centre.

Tauc plot method for determining absorption onset

UV spectra were collected over a range of concentrations ([Ti] = 1.75-0.05 mM). All results obey Beer-Lambert behaviour. The strongest concentrations were used to calculate the absorption onset using the Tauc plot method.[2, 3] The absorption onset is described by:

$$A = \frac{B(hv - E_g)^n}{hv}$$

Where *A* is absorption, *B* is the absorption constant for the transition, E_g is the energy gap in eV and hv is the photon energy. Absorption was most accurately modelled with a cubic dependence on increasing energy (Fig. S32), in keeping with previous studies of titanium-oxo clusters,[4] and therefore the exponent *n* is given a value of 3 (for bulk semiconductors this is considered a forbidden direct transition). A plot of $(Ahv)^{1/3}$ vs hv gives a straight line (Fig S8). The point of absorption onset (in eV) was derived at the x-value of the intercept of this straight line with a straight line describing the baseline (to avoid any error from extrapolating through an imperfect baseline).[3]

Computational information

All density functional theory (DFT) calculations were performed with the Amsterdam Modelling Suite 2021 (AMS2021).[5, 6] Geometries were optimized using the TZ2P basis set (no frozen core),[7] the PBE functional,[8] with inclusion of the ZORA scalar relativistic corrections,[9-11] and NumericalQuality setting to Good. UV/Vis spectra were calculated using the Time-dependent DFT (TDDFT) approach,[12, 13] with AMS2021 and the same basis set/functional combination as the geometry optimizations. Cartesian coordinates of all optimized structures are in the Supporting Information.

Additional spin polarized TPSSh/TZ2P//PBE/TZ2P calculations were performed for **2** and **3** (and also B3LYP/TZ2P for **3**) to determine the extent of spin symmetry breaking. EPR parameters[14] were evaluated for the triplet state of **3** using the PBE functional and TZ2P-J basis set.

Preparation of compounds

Synthesis of **1** (*adapted from Piszczek et al.*[15])

3.6 g (35.2 mmol) of pivalic acid (^tBuCOOH) was put into a nitrogen filled Schlenk flask and dissolved in 30 mL toluene. 10 g (35.2 mmol) of $Ti(O^iPr)_4$ was transferred to a second Schlenk flask with a stirrer bar, and dissolved in 20 mL toluene. The pivalic acid solution was then added dropwise to the $[Ti(O^iPr)_4]$ solution and the mixture stirred for 30 minutes. In a separate Schlenk flask 0.63 mL (35.2 mmol) of water was added to 15 mL of dry acetone and this mixture added slowly to the reaction mixture over 60 minutes, slow dropwise addition was achieved using a thin cannula (internal diameter 0.3 mm). The reaction solution was then heated at 60°C whilst stirring for 24 h. On cooling to room temperature **1** precipitates from solution as a colourless crystalline material, the flask was cooled to -20°C overnight to optimize yield. 4.3 g of crystalline product was dried under vacuum and isolated from the flask (55% yield).

^{1}H NMR spectroscopy ($CDCl_3$, 400 MHz): δ 4.90 (6H, septet (J_{HH} = 6 Hz), OC**H**Me_2); 1.38 (36H, d (J_{HH} = 6 Hz), OCH**Me_2**; 1.12 (54H, s, O_2C**^{t}Bu**).

^{1}H NMR spectroscopy (d^8-toluene, 400 MHz): δ 5.06 (6H, septet (J_{HH} = 6 Hz), OC**H**Me_2); 1.53 (36H, d (J_{HH} = 6 Hz), OCH**Me_2**); 1.26 (54H, s, O_2C**^{t}Bu**).

Elemental Analysis (predicted for **1**): % C, 42.99 (42.88); % H, 7.12 (7.20). N.B. no solvent of crystallisation is observed by ^{1}H NMR or elemental analysis in the dried isolated compound.

ESI Mass Spectrum: [**1**-H]$^+$ predicted 1345.32, found 1345.32.

Synthesis of **2**

300 mg (0.216 mM) of **1** was dissolved in 6 mL of toluene in a small Schlenk flask. To this, 0.83 mL (10.8 mM) of isopropanol (~50 equiv.) was added. The flask was freeze thaw degassed twice and refilled with N_2. The solution was gently heated to ensure solvation of **1** and then placed under a 302 nm UV lamp for 4-6.5 hours. The flask was allowed to stand overnight and then any solubles decanted leaving blue/black crystalline material which was dried under vacuum. After 4 hours, 180 mg isolated yield (52%); after 6.5 hours, 255 mg isolated yield (73%) but containing ~1% of unknown paramagnetic by-product.

^{1}H NMR spectroscopy (d^8-toluene, 400 MHz): δ 8.38 (4H, very broad, ^{i}PrO**H**), 5.09 (6H, septet, (J_{HH} = 6 Hz), Ti-OC**H**Me_2); 3.53 (2H, br, H-OC**H**Me_2); 1.60 (36H, d (J_{HH} = 6 Hz), Ti-OCH**Me_2**); 1.24 (54H, s, O_2C**^{t}Bu**); 1.00 (12H, d (J_{HH} = 6 Hz), H-OCH**Me_2**).

Elemental Analysis (predicted for **2**·$(toluene)_{0.2}$): % C, 43.43 (43.47); % H, 7.09 (7.36). Note that in the single-crystal solid-state structure a formula of **2**·$(^iPrOH)_2$(toluene) is observed, it is anticipated that the ^{i}PrOH and majority of toluene is lost under extended vacuum.

Synthesis of **3**

300 mg (0.216 mM) of **1** was dissolved in 6 mL of toluene in a small Schlenk flask. To this, 0.9 mL (11.2 mM) of pyridine (~50 equiv.) was added. The flask was freeze thaw degassed twice and refilled with N_2. The solution was gently heated to ensure solvation of **1** and then placed under a 302 nm UV lamp for 6.5 hours at ambient temperature. Crystalline product forms directly on the sides of the flask. The

purple solution was decanted off and discarded and the solid product dried under vacuum. 195 mg purple/black crystalline product (59% isolated yield)

^{1}H NMR spectroscopy (d^8-toluene, 400 MHz): δ 9.77 (4H, d (J_{HH} 5 Hz), py (2,6)); 7.06 (4H, part obscured), py (3,5)); 6.99 (2H, part obscured), py (4)); 5.28 (2H, septet (J_{HH} = 6 Hz), OC**H**Me$_2$); 4.87 (2H, septet (J_{HH} = 6 Hz), OC**H**Me$_2$); 1.60 (6H, d (J_{HH} = 6 Hz), OCH**Me$_2$**); 1.55 (6H, d (J_{HH} = 6 Hz), OCH**Me$_2$**); 1.52 (9H, s, O$_2$C^t**Bu**); 1.44 (6H, d (J_{HH} = 6 Hz), OCH**Me$_2$**); 1.26 (18H, s, O$_2$C^t**Bu**); 1.23 (9H, s, O$_2$C^t**Bu**); 1.19 (6H, d (J_{HH} = 6 Hz), OCH**Me$_2$**); 1.01 (18H, s, O$_2$C^t**Bu**).

Elemental Analysis (predicted for **3**·(toluene)$_{0.2}$): % C, 45.70 (45.72); % H, 6.65 (6.73); % N 1.67 (2.00). Note that in the single-crystal solid-state structure a formula of **3**·toluene is observed, it is anticipated that the majority of toluene is lost under extended vacuum.

Synthesis of **4** (as mixture with **1**)

100 mg (0.072 mM) of **1** was dissolved in 2 mL of toluene in a small Schlenk flask. To this, 0.5 mL (6.2 mM) of THF (~85 equiv.) was added. The flask was freeze thaw degassed twice and refilled with N_2. The solution was gently heated to ensure solvation of **1** and then placed under a 302 nm UV lamp for 4 hours at ambient temperature. A dark blue solution forms with some dark precipitate. The solution was concentrated to ~0.5 mL and then remaining soluble were discarded. The resulting dark blue solid contained ~3:1 ratio of **4** and **1**.

^{1}H NMR spectroscopy (d^8-toluene, 400 MHz): δ 5.30 (2H, septet (J_{HH} = 6 Hz), OC**H**Me$_2$); 5.16 (2H, septet (J_{HH} = 6 Hz), OC**H**Me$_2$); 4.35 (4H, m, 1,4-THF); 4.08 (4H, m, 1,4-THF); 1.79 (8H, m, 2,3-THF); 1.57 (6H, d (J_{HH} = 6 Hz), OCH**Me$_2$**); 1.55 (6H, d (J_{HH} = 6 Hz), OCH**Me$_2$**); 1.53 (6H, d (J_{HH} = 6 Hz), OCH**Me$_2$**); 1.52 (9H, s, O$_2$C^t**Bu**); 1.44 (6H, d (J_{HH} = 6 Hz); 1.38 (9H, s, O$_2$C^t**Bu**); 1.27 (18H, s, O$_2$C^t**Bu**); 1.06 (18H, s, O$_2$C^t**Bu**).

Synthesis of **5**

Air was dried by sealing a flask containing activated 4Å molecular sieves. This dry air was transferred into an evacuated flask containing **3** either in the solid state as a powder or as a solution in toluene. **5** can be recrystallized from minimal CH_2Cl_2 at -30 °C.

^{1}H NMR spectroscopy (d^8-toluene, 400 MHz): δ 9.53 (2H, d (J_{HH} 5 Hz), py (2,6)); 6.95 (2H, t d (J_{HH} 7 Hz),), py (4)); 6.78 (2H, dd, py (3,5)); 5.36 (1H, septet (J_{HH} = 6 Hz), OC**H**Me$_2$); 5.24 (1H, septet (J_{HH} = 6 Hz), OC**H**Me$_2$); 4.98 (1H, septet (J_{HH} = 6 Hz), OC**H**Me$_2$); 4.79 (2H, septet (J_{HH} = 6 Hz), OC**H**Me$_2$); 1.65-1.50 (15H, 5x d (J_{HH} = 6 Hz), OCH**Me$_2$**); 1.38-1.20 (9H, 3x d (J_{HH} = 6 Hz), OCH**Me$_2$**); 1.31 (18H, s, O$_2$C^t**Bu**); 1.26 (9H, s, O$_2$C^t**Bu**); 1.25 (18H, s, O$_2$C^t**Bu**); 1.13 (9H, s, O$_2$C^t**Bu**).

Elemental Analysis, sample prepared by oxidation of **3** by dry air in the solid-state followed by extended vacuum. (predicted for **3**·(toluene)$_{0.3}$): % C, 43.48 (43.20); % H, 6.33 (6.60); % N 0.96 (1.03). Note that in the solid-state structure of oxidised **3** will be **5**·(toluene)(pyridine), it is anticipated that the pyridine and majority of toluene is lost under extended vacuum.

Synthesis of **1***

250 mg (0.186 mmol) of **1** was dissolved in 5 mL toluene and a large excess (2.56 mL, 25 mmol) of 4-penten-1-ol was added. The solution was heated to 70 °C for 6 days and then reduced to dryness under vacuum. 70 mg (25% yield) of white powder was isolated.

^{1}H NMR spectroscopy (d^8-toluene, 400 MHz): δ 5.93 (6H, m, =C**H**R); 5.15 (6H, d (J_{HH} = 17 Hz), =C**H**H'); 5.02 (6H, d (J_{HH} = 10 Hz), =CH**H'**); 4.74 (12H, t (J_{HH} = 6 Hz), OC**H₂**R); 2.39 (12H, q (J_{HH} = 7 Hz), $OCH_2CH_2C\mathbf{H_2}CHCH_2$); 1.91 (12 H, m, $OCH_2C\mathbf{H_2}CH_2CHCH_2$); 1.27 (54H, s, O_2C^t**Bu**)

Elemental Analysis (predicted for **1***): % C, 47.18 (48.02); % H, 7.02 (7.25).

Synthesis of 1 via $[Ti_2(OR)_6(O_2CCMe_3)_2(HOR)]$

The initial reaction of $[Ti(O^iPr)_4]$ with 1 equiv. of pivalic acid (^{t}BuCOOH) in toluene at room temperature produces the previously reported structure $[Ti_2(OR)_6(O_2CCMe_3)_2(HOR)]$,[16] in which one carboxylate bridges the two metal centres and the other coordinates in a monodentate fashion to one Ti atom. This compound was confirmed by X-ray diffraction of crystals grown from the reaction at this stage (Fig. S1).[16] Whilst leaving the reaction mixture for several days has been reported to form Ti-oxo clusters, via an esterification reaction which provides water,[15, 17, 18] we chose to explicitly add 1 equivalent of water (dissolved in acetone) dropwise to the solution to induce hydrolysis without the need of esterification reactions (which consume some carboxylic acid). Heating the solution at 60°C overnight ensures full conversion to the product **1**.

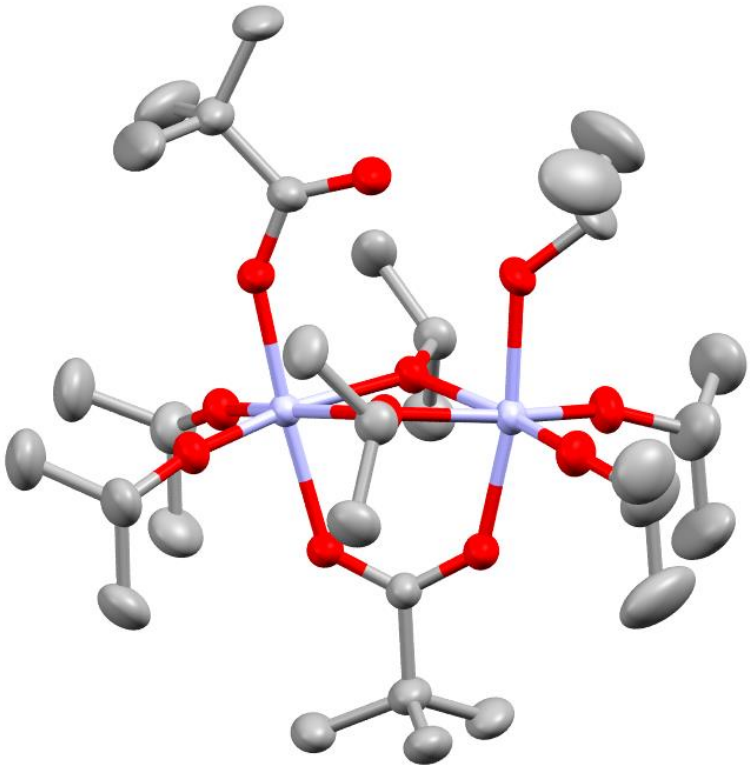

Fig S1. X-ray crystal structure of $Ti_2(OR)_6(O_2CCMe_3)_2(HOR)$. Ellipsoids displayed at 50%.

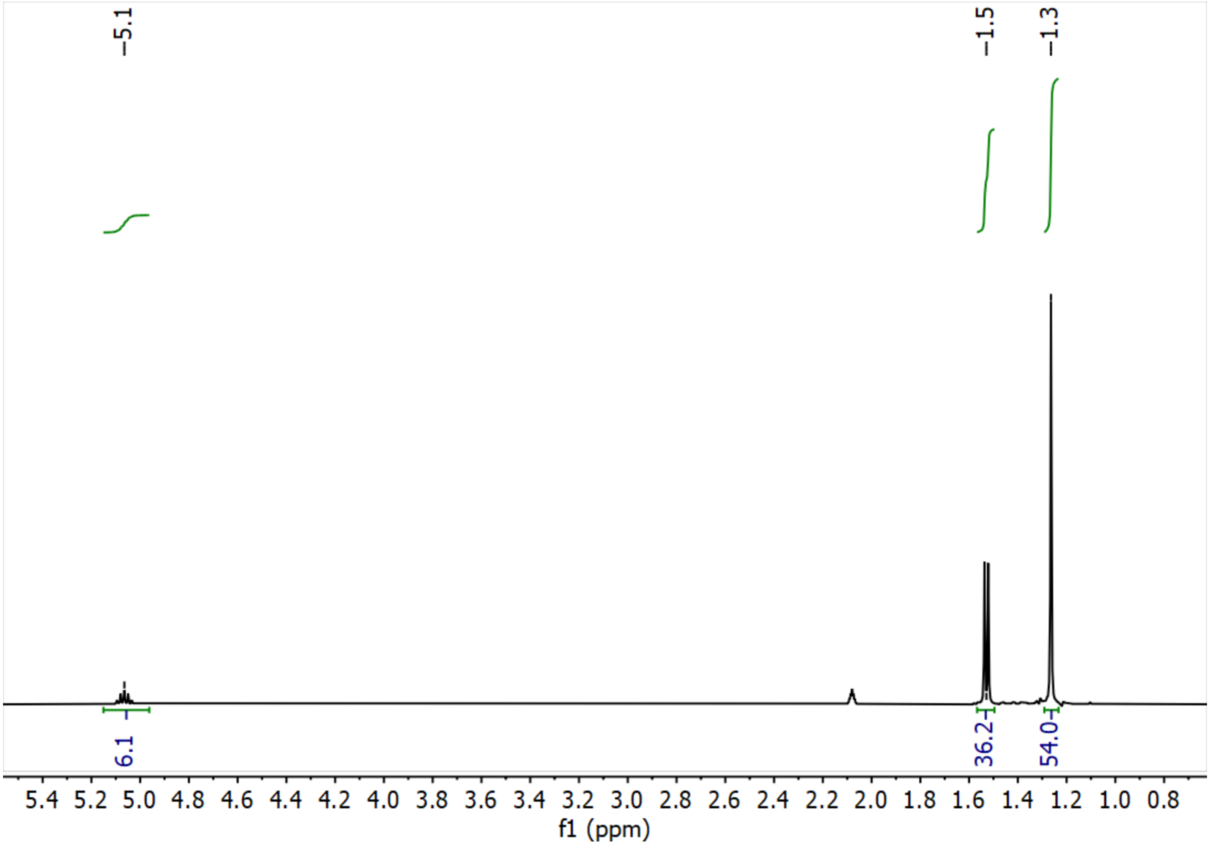

Fig S2. ^{1}H NMR spectrum of **1** in d^{8}-toluene

Crystallography of 1.

Single crystals of **1** grown from toluene were typically found to adopt a trigonal space group (R–3) as had been previously reported.[15] However, an alternative crystalline structure was observed upon crystallising from a toluene/pyridine mixture, this triclinic polymorph of **1** did not contain any solvent molecules. The powder diffraction pattern of bulk **1** indicates both polymorphs are present (see Figure S5). The trigonal crystals were particularly delicate and sensitive to cracking at low temperature, therefore, for these crystals data was collected at 150 K (rather than 100 K), data quality was lower than that of the triclinic crystals. Bond length and angle data were taken from the triclinic data set.

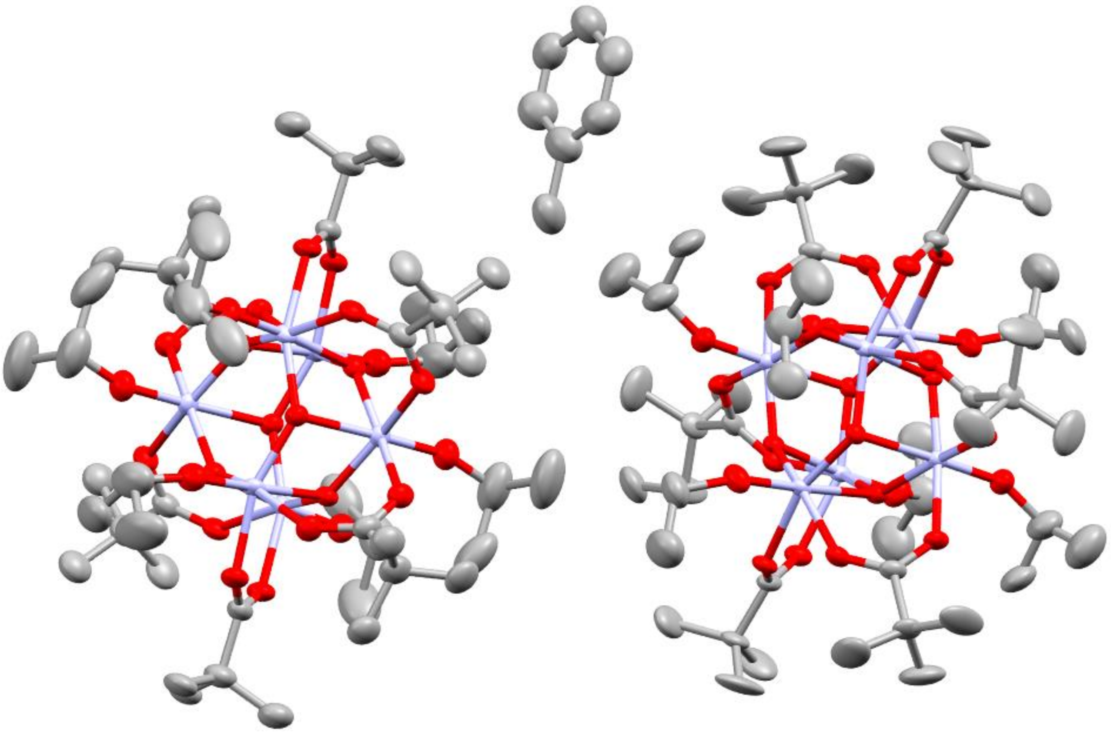

Figure S3. Single crystal structure of **1**, crystallised with a molecule of toluene per cluster in a trigonal space group. Ellipsoids shown at 50% probability and hydrogen atoms omitted for clarity.

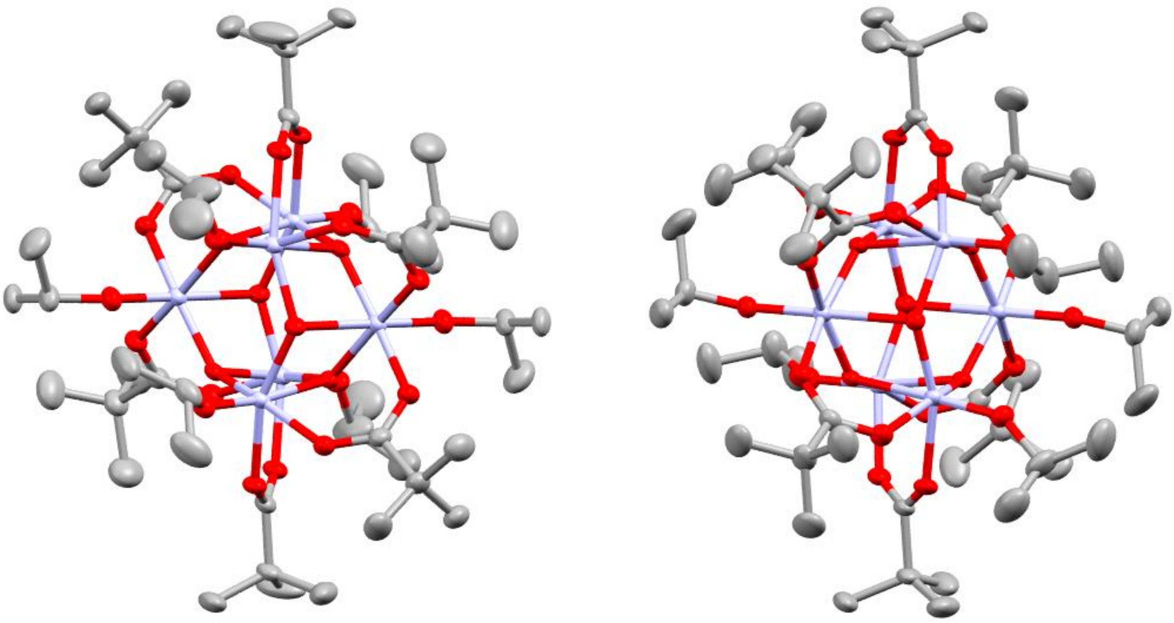

Figure S4. Single crystal structure of **1**, crystallised without solvent molecules in a triclinic space group. Ellipsoids shown at 50% probability and hydrogen atoms omitted for clarity.

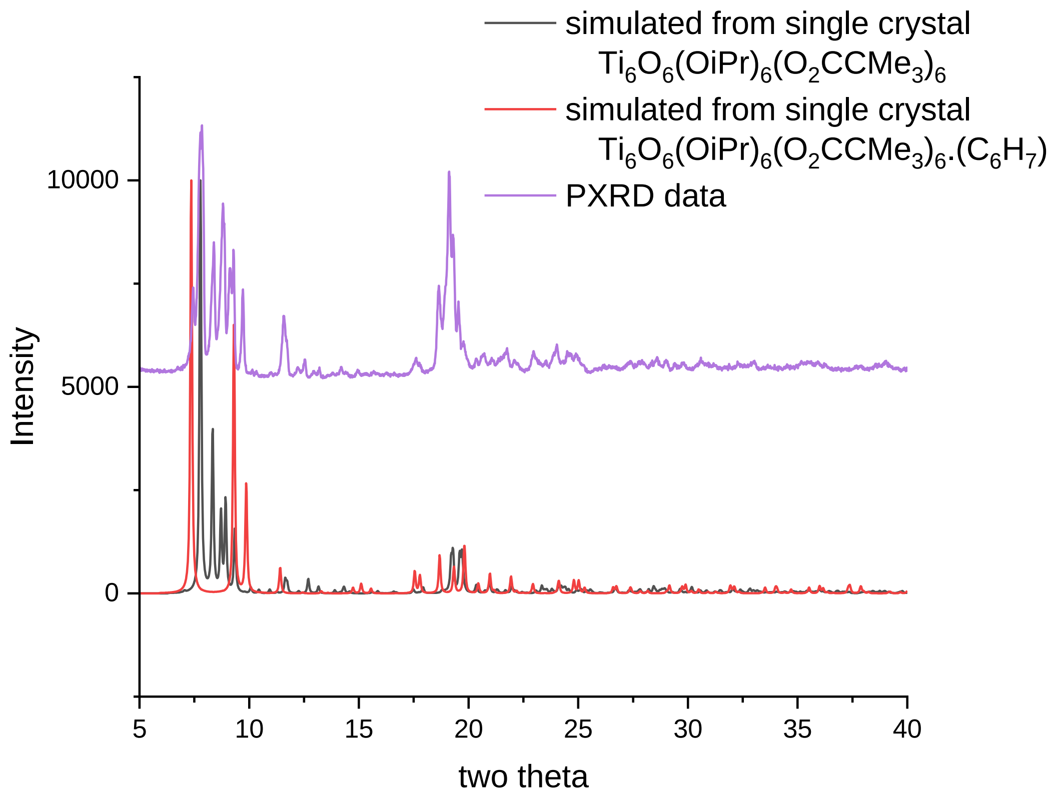

Fig S5. Powder X-ray diffraction data of a bulk batch of **1** compared to simulated data from single crystal diffraction data of two polymorphs of **1** (with and without toluene solvent of crystallisation).

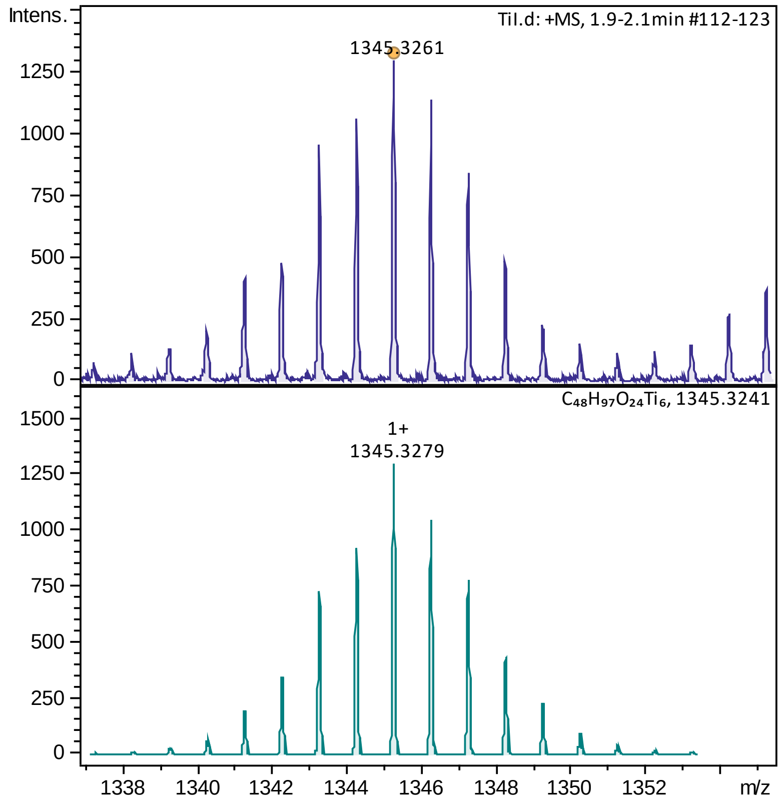

Fig S6. ESI mass spectrum of [**1**+H]$^+$.

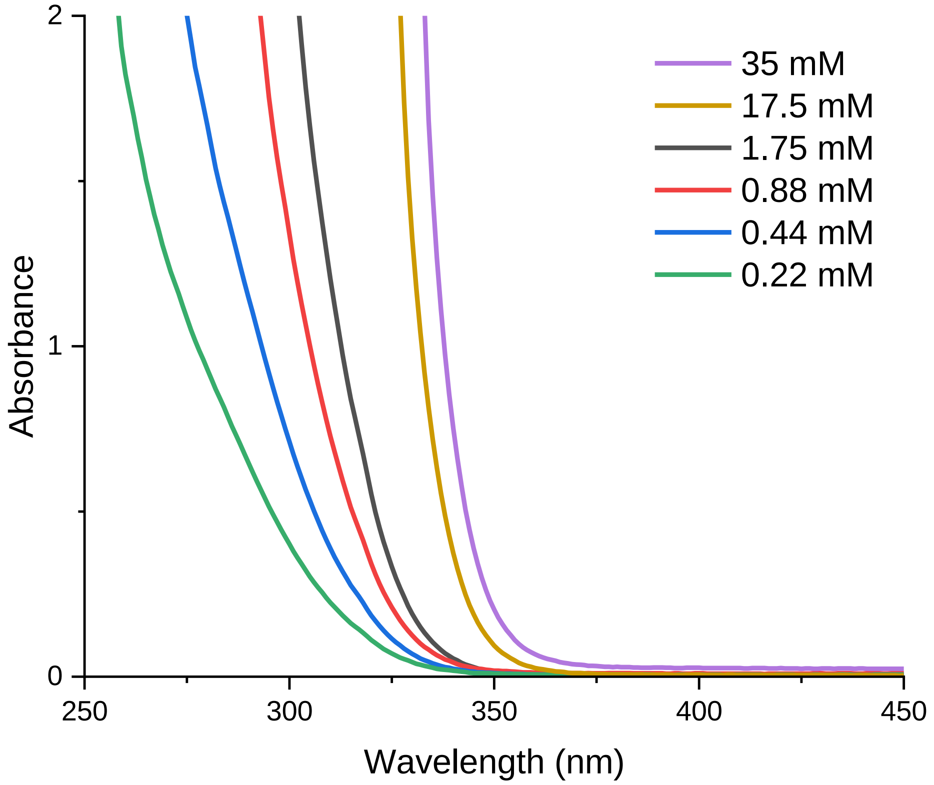

Fig S7. UV spectrum of **1** at varying concentrations. Note that higher concentrations show the absorption onset more accurately.

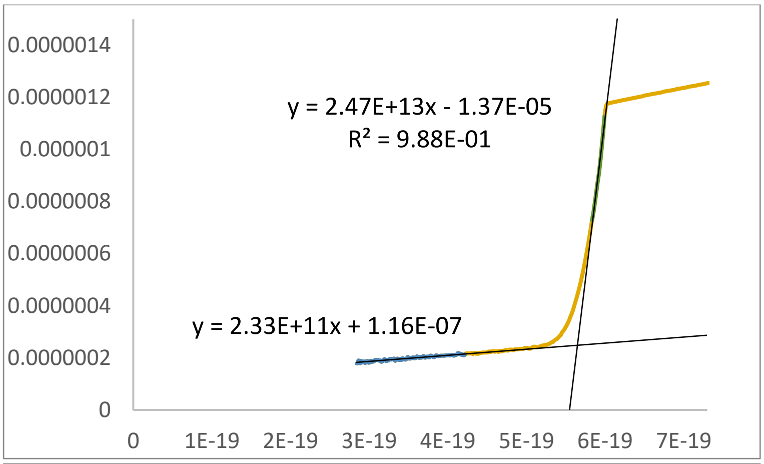

0.0000014
0.0000012
0.000001
0.0000008
0.0000006
0.0000004
0.0000002
0
0
1E-19
2E-19
3E-19
4E-19
5E-19
6E-19
7E-19
y = 2.47E+13x - 1.37E-05
R² = 9.88E-01
y = 2.33E+11x + 1.16E-07

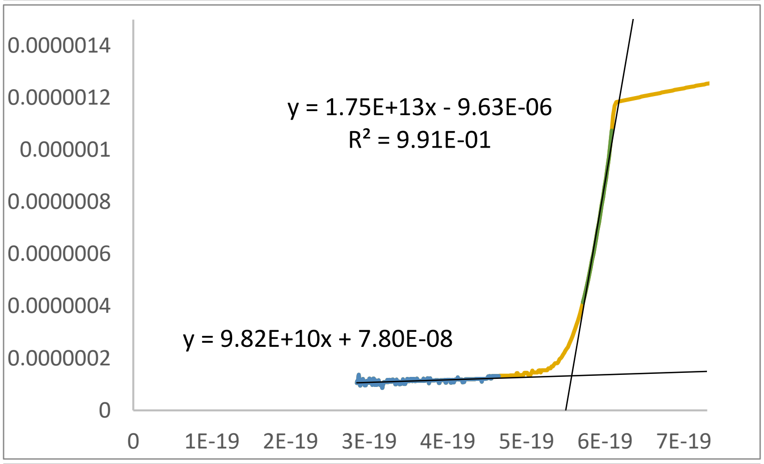

0.0000014
0.0000012
0.000001
0.0000008
0.0000006
0.0000004
0.0000002
0
0
1E-19
2E-19
3E-19
4E-19
5E-19
6E-19
7E-19
y = 1.75E+13x - 9.63E-06
R² = 9.91E-01
y = 9.82E+10x + 7.80E-08

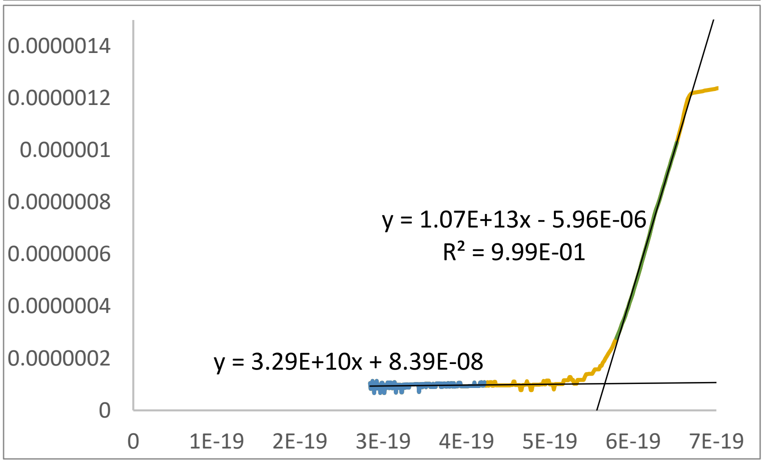

0.0000014
0.0000012
0.000001
0.0000008
0.0000006
0.0000004
0.0000002
0
0
1E-19
2E-19
3E-19
4E-19
5E-19
6E-19
7E-19
y = 1.07E+13x - 5.96E-06
R² = 9.99E-01
y = 3.29E+10x + 8.39E-08

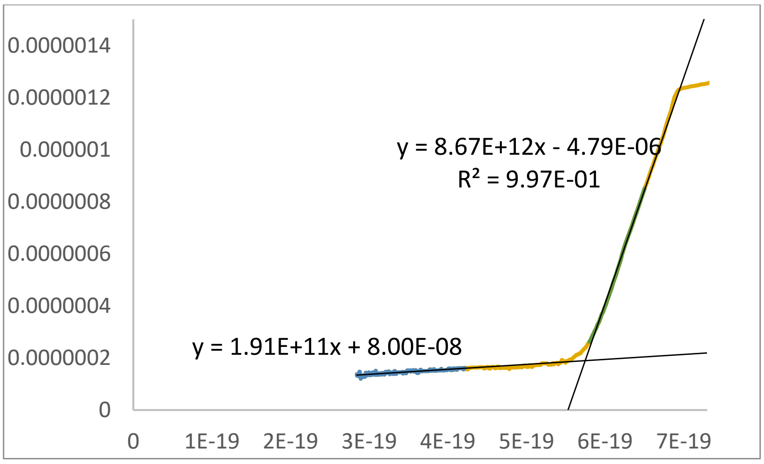

0.0000014
0.0000012
0.000001
0.0000008
0.0000006
0.0000004
0.0000002
0
0
1E-19
2E-19
3E-19
4E-19
5E-19
6E-19
7E-19
y = 8.67E+12x - 4.79E-06
R² = 9.97E-01
y = 1.91E+11x + 8.00E-08

Concentration	Absorption onset estimated by Tauc plot method
35 mM	3.51
17.5 mM	3.50
1.75 mM	3.54
0.88 mM	3.55

Fig S8. Tauc plot data (on previous page) and summary table for UV-vis spectra with [Ti] = 35 mM, 17.5 mM, 1.75 mM and 0.88 mM, using n = 3.

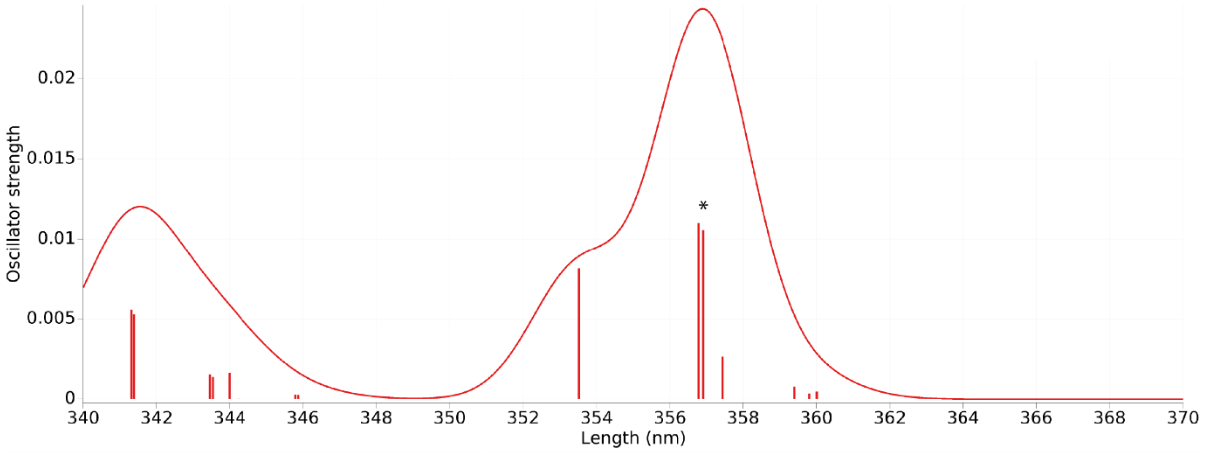

Fig S9. Calculated UV/Vis spectrum for **1** (Gaussian width 3.0 nm). Indicated with an asterisk (*) are excited states 11 and 12, which have a considerable oscillator strength.

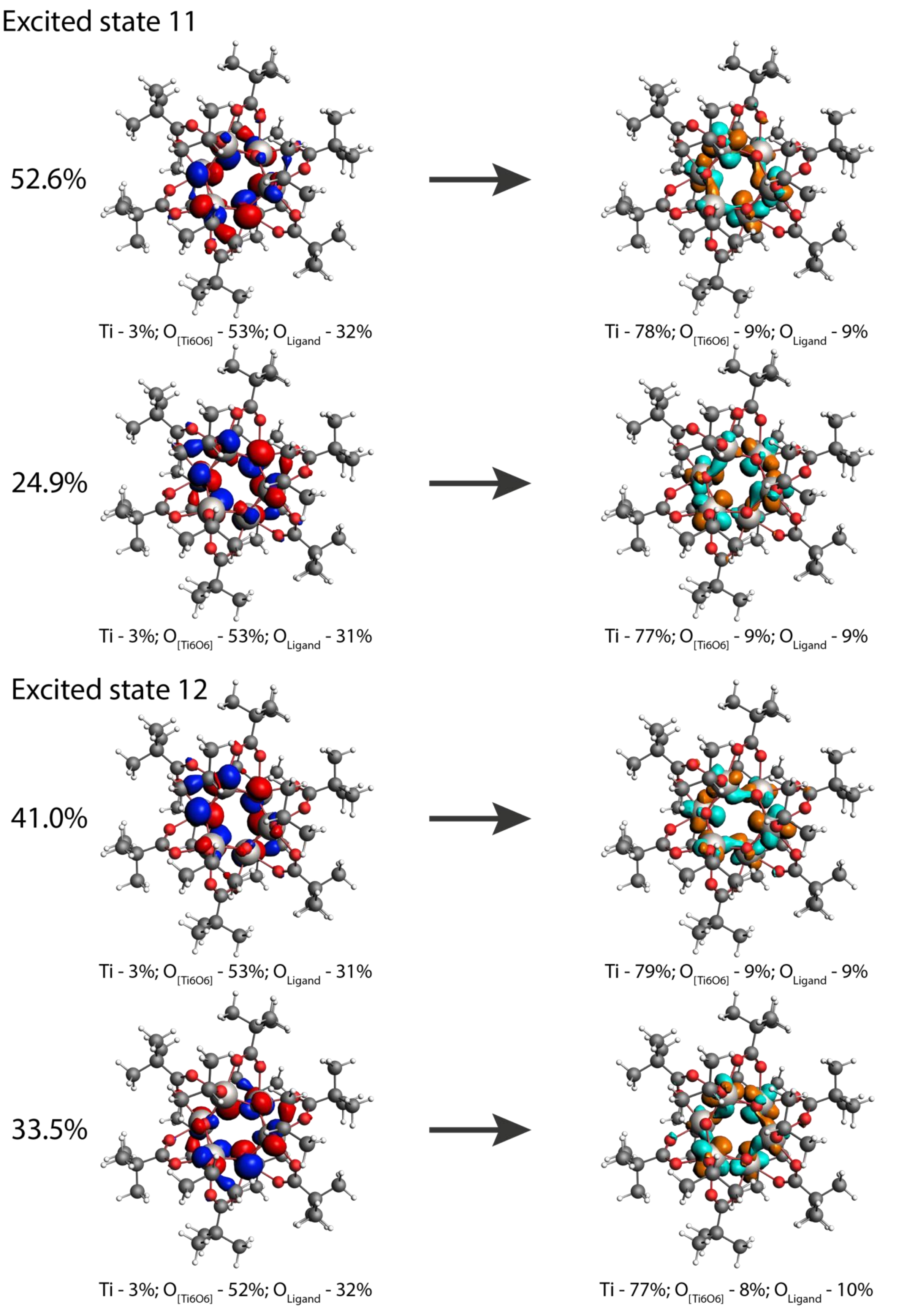

Fig S10. Natural transition orbitals for the excited states 11 and 12 with significant oscillator strength and their percentage in the transition (red/blue donor orbital, cyan/orange acceptor orbital).

In-situ NMR spectroscopy analysis of photoredox reaction of 1.

NMR spectra were referenced to the methyl signal of d^8-toluene at 2.08 ppm, this signal was integrated to a value of 1 as an internal standard. Integrals of other signals were normalized to account for no. of protons in the environment and converted to a percentage based on the value of the starting material (**1**) at time = 0. For species with multiple proton environments the average of clearly defined normalized signals is used. The reaction was assumed to proceed by the reaction **1** → photoproduct + acetone + ^{i}PrOH. Overlapping peaks in the 1-2 ppm area reduce accuracy of integrals, often resulting in an overestimate of the acetone (1.57 ppm) signal. Rates of photoreaction were found to be slightly variable depending on flask position relative to the lamp and accelerated by adding reflective foil behind the sample flask.

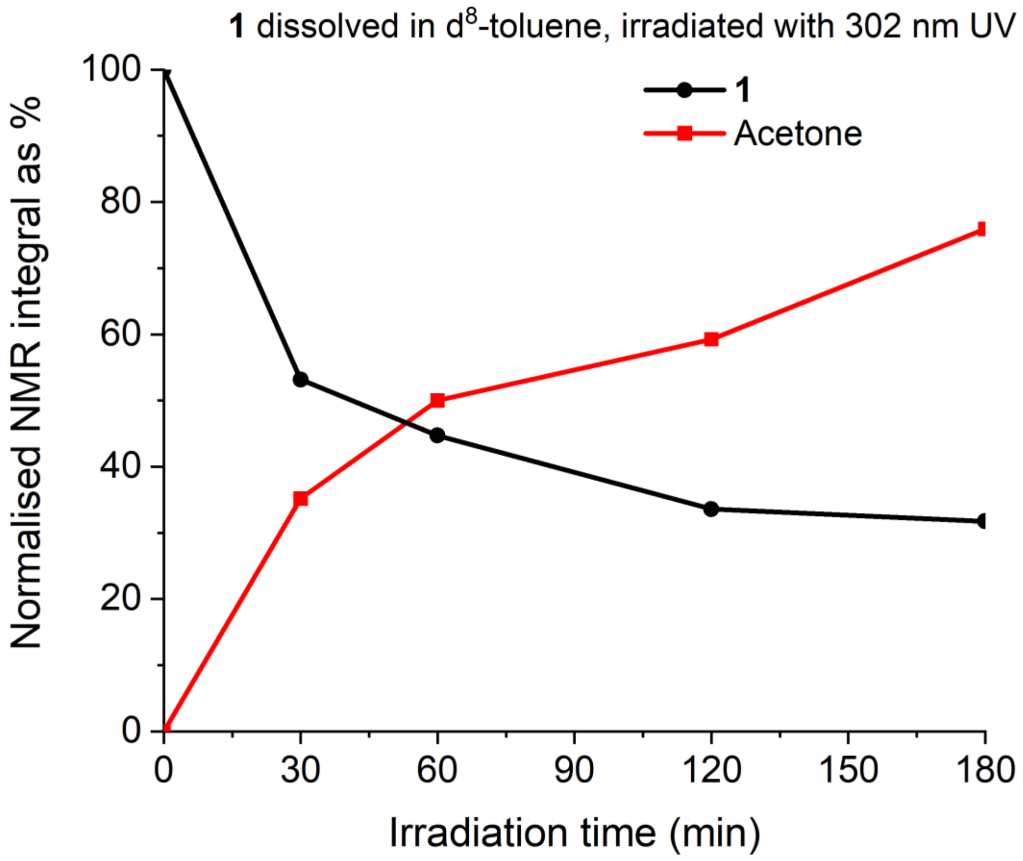

Fig S11. Normalised ^{1}H NMR integrals of **1** and acetone during the Irradiation of a solution of **1** in toluene with 302 nm UV light.

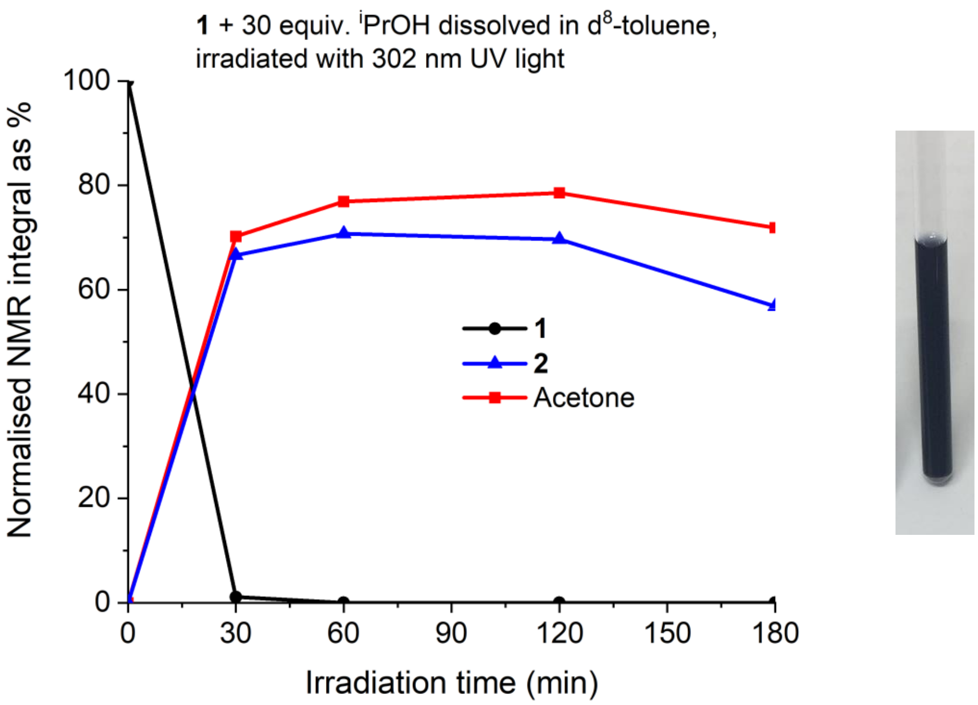

Fig S12. Normalised ^{1}H NMR integrals of **1**, **2** and acetone during the Irradiation of a solution of **1** in toluene with 30 equiv. ^{i}PrOH with 302 nm UV light. Note that **2** begins to visibly precipitate over time, reducing the signal observed by solution NMR spectroscopy. Photograph of solution in NMR tube after irradiation.

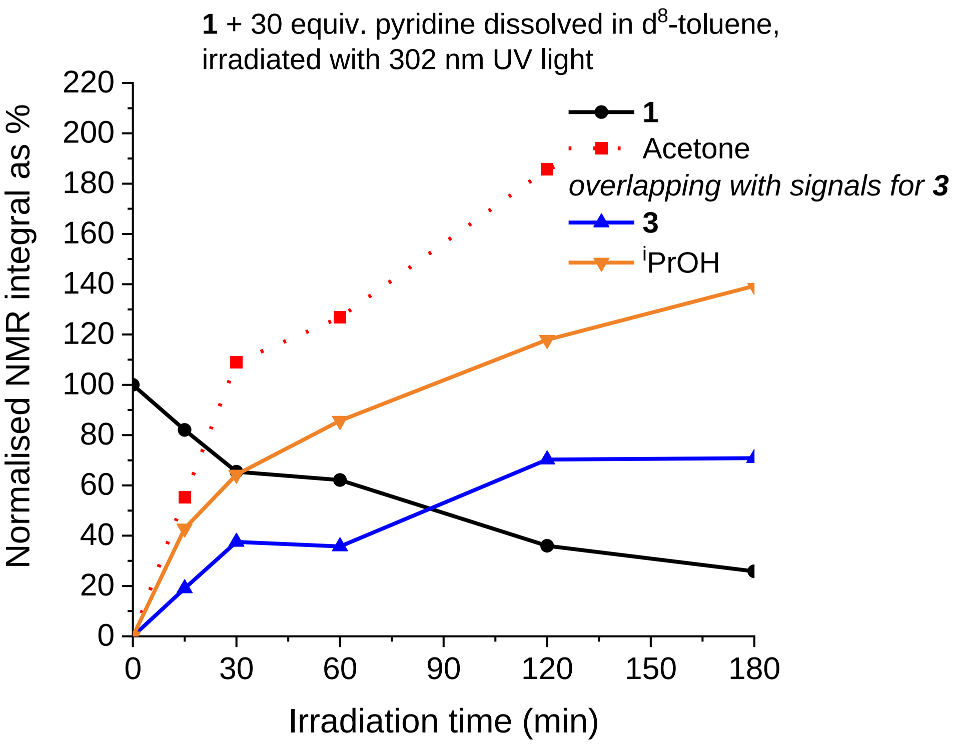

Fig S13. Normalised ^{1}H NMR integrals of **1**, **3**, ^{i}PrOH and acetone during the Irradiation of a solution of **1** in toluene with 30 equiv. pyridine with 302 nm UV light. Note that acetone integrals overlap with new O^{i}Pr environment of **3**, hence give an overestimate.

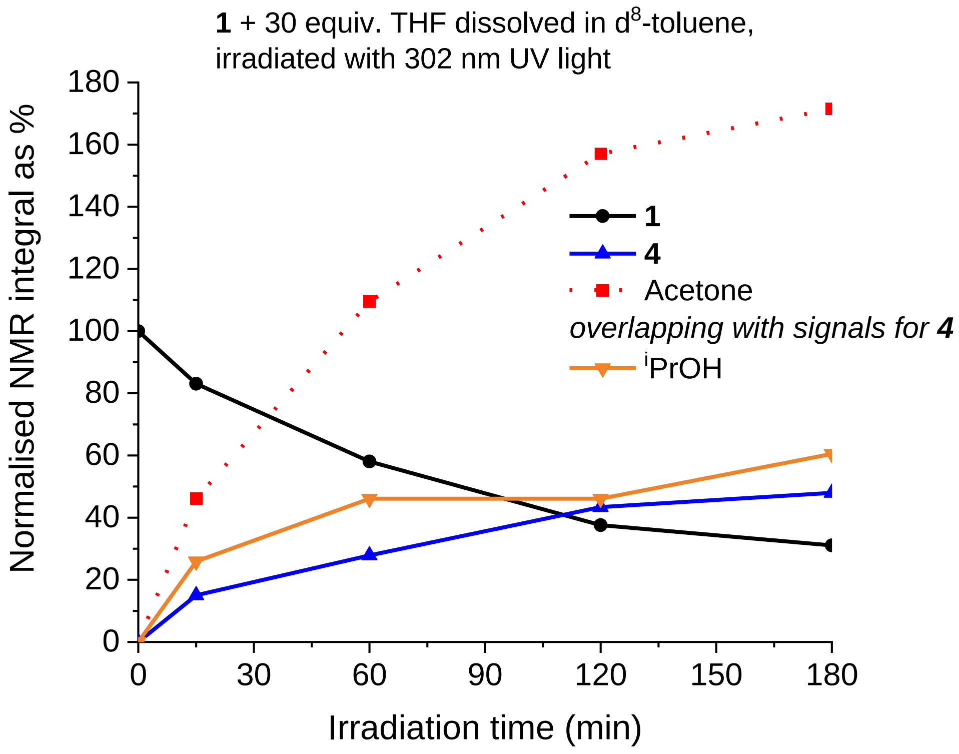

Fig S14. Normalised ^{1}H NMR integrals of **1**, **4**, iPrOH and acetone during the Irradiation of a solution of **1** in toluene with 30 equiv. THF with 302 nm UV light. Note that acetone integrals overlap with new OiPr environment of **4**, and therefore give an overestimate.

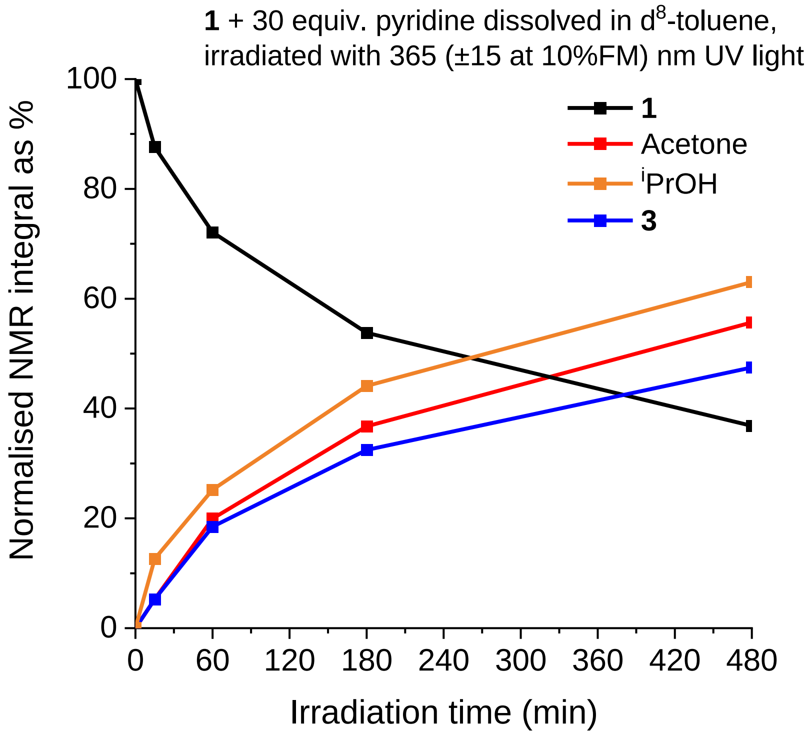

Fig S15. Normalised ^{1}H NMR integrals of **1**, **3**, iPrOH and acetone during the Irradiation of a solution of **1** in toluene with 30 equiv. pyridine with 365 nm UV light.

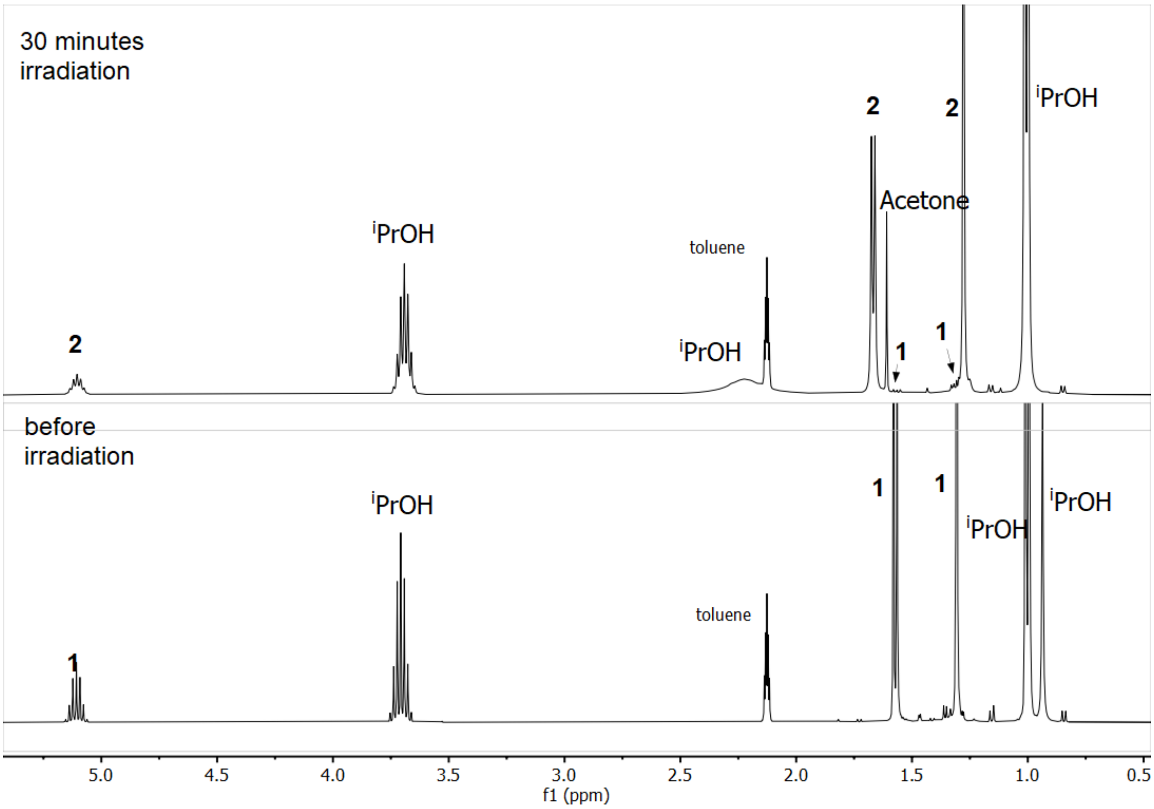

Fig S16. ^{1}H NMR spectrum of a solution of **1** in toluene with 30 equiv. ^{i}PrOH before and after irradiated with 302 nm light for 30 minutes.

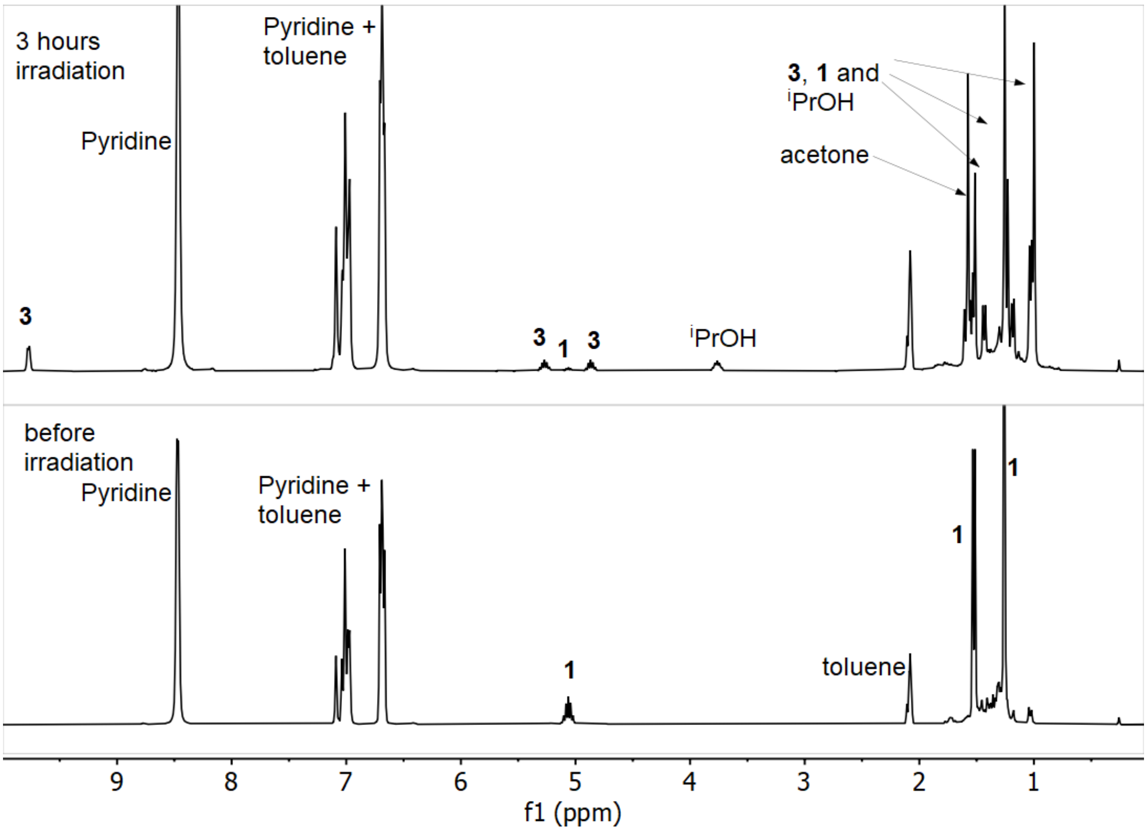

Fig S17. ^{1}H NMR spectrum of a solution of **1** in toluene with 30 equiv. pyridine before and after irradiated with 302 nm light for 3 hours.

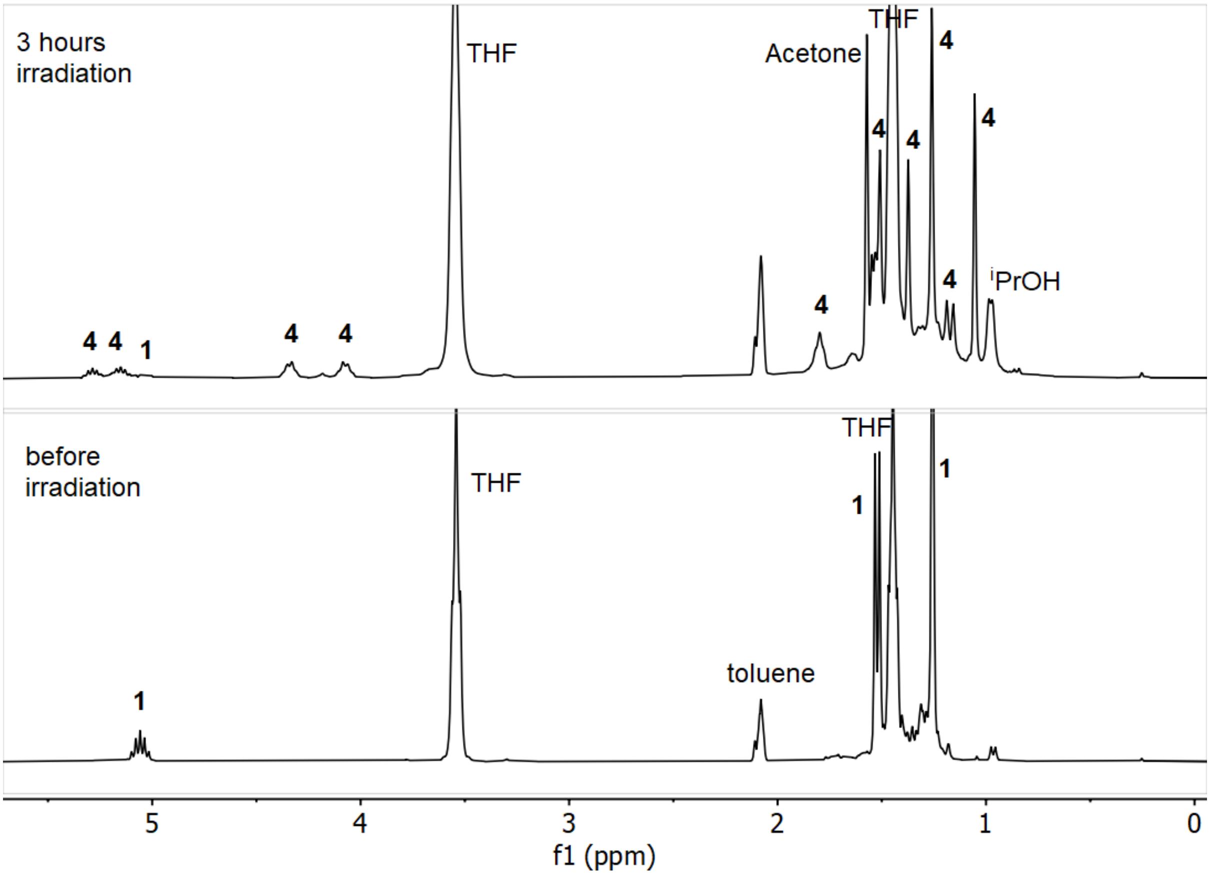

Fig S18. ^{1}H NMR spectrum of a solution of **1** in toluene with 30 equiv. THF before and after irradiated with 302 nm light for 3 hours.

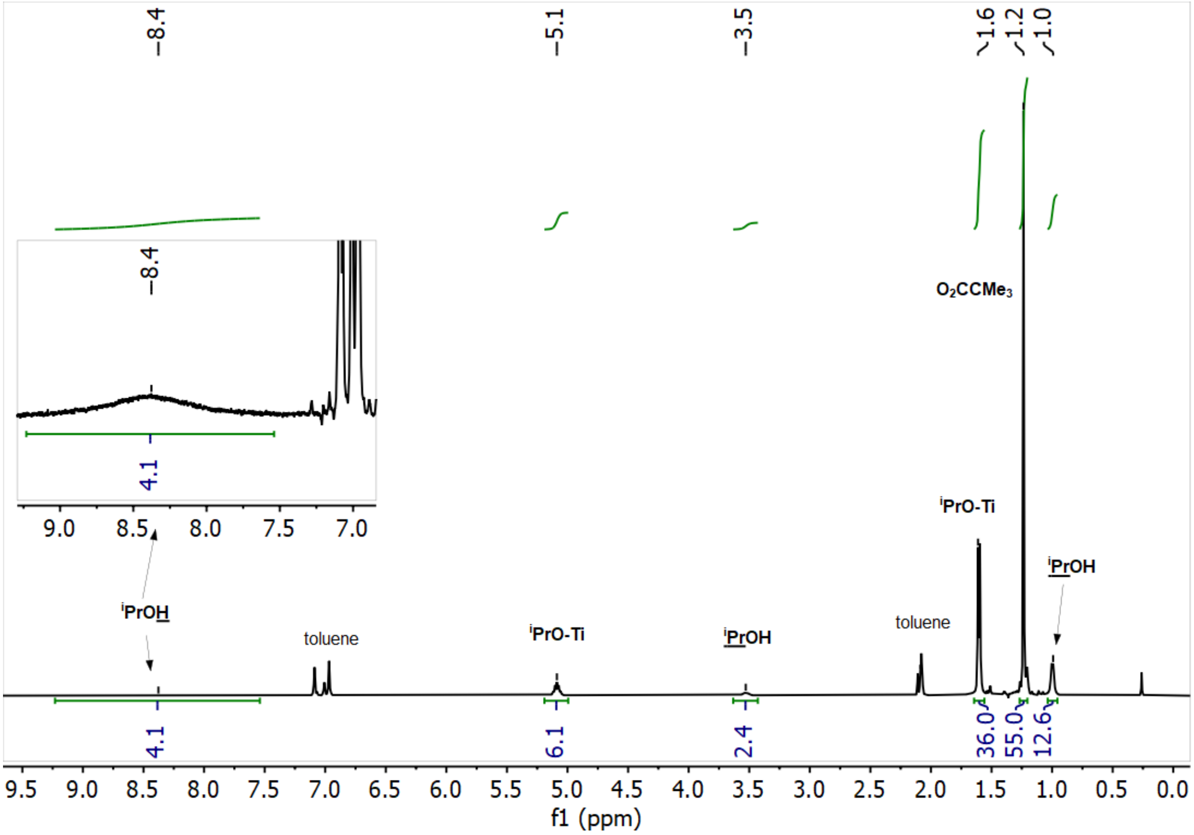

Fig S19. ^{1}H NMR spectra of **2** in d^{8}-toluene.

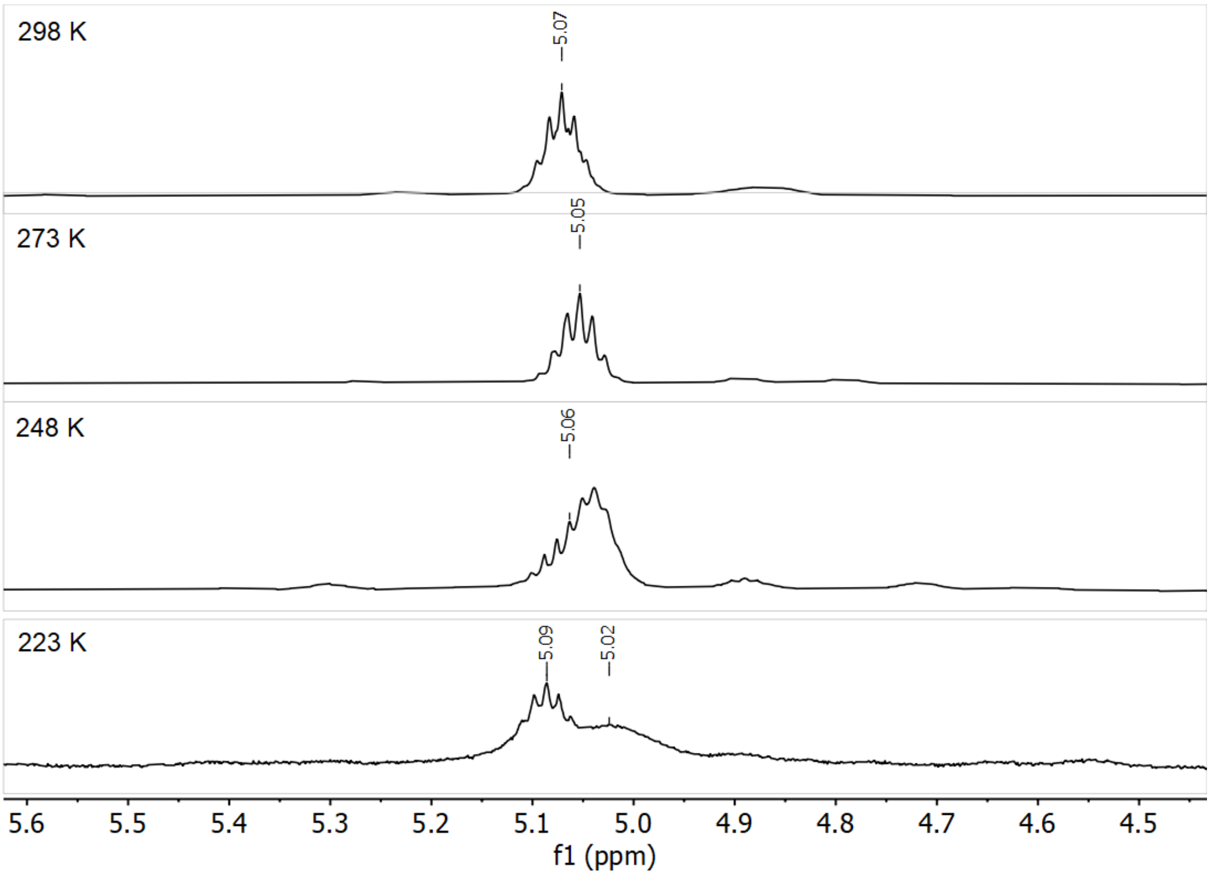

Fig S20. ^{1}H NMR spectra of **2** in d^{8}-toluene (Ti-O^{i}Pr region) at various temperatures.

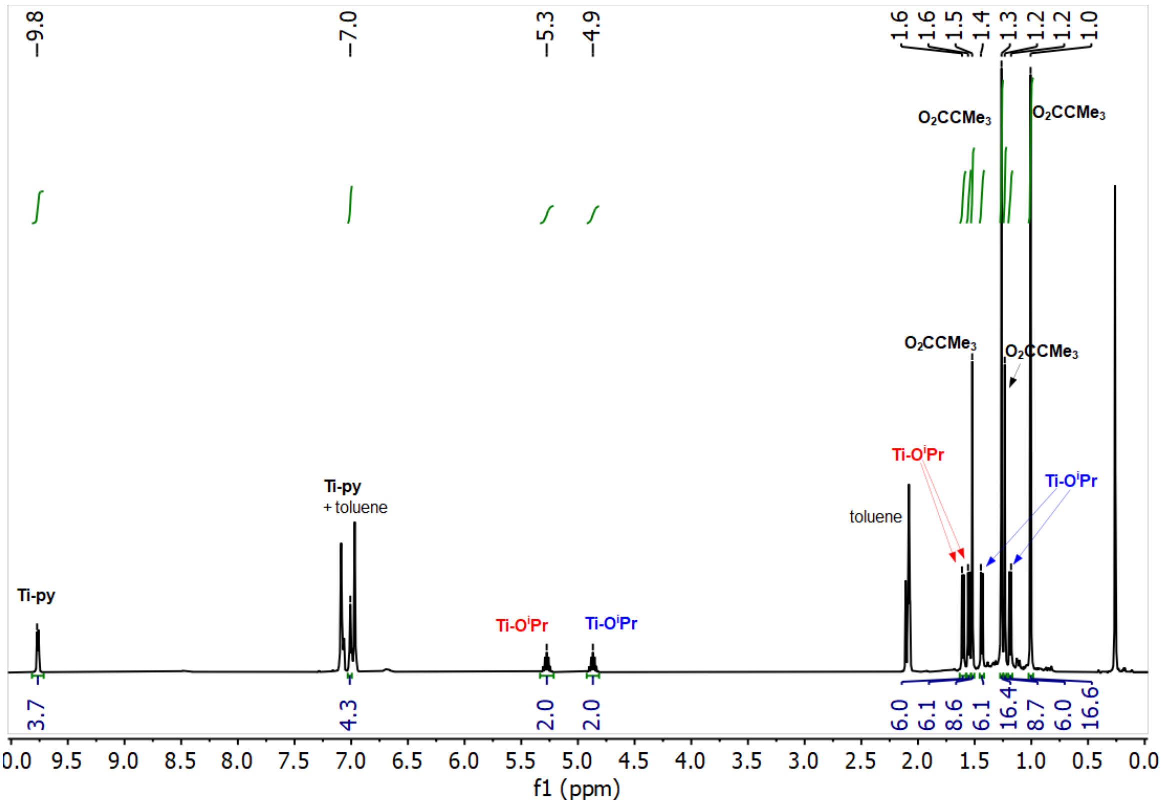

Fig S21. ^{1}H NMR spectra of **3** in d^8-toluene.

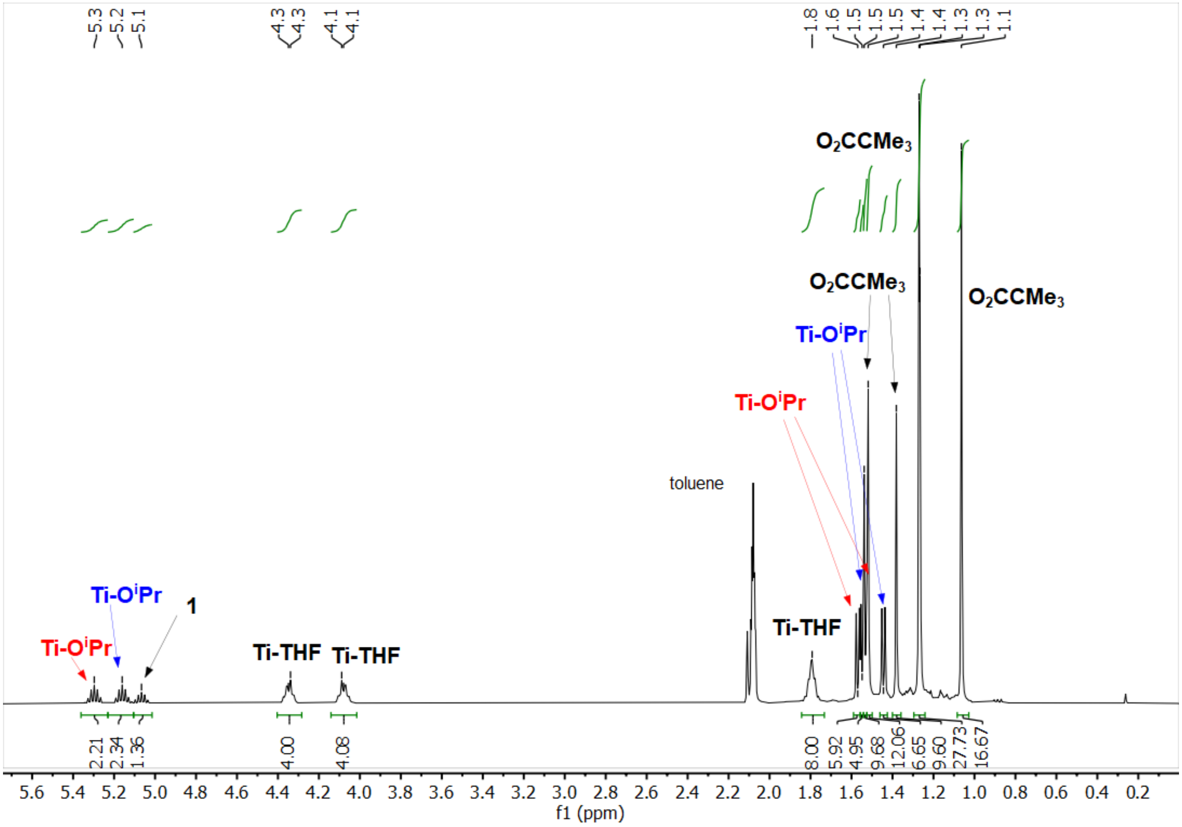

Fig S22. ^{1}H NMR spectra of **4** in d^8-toluene, along with **1** as minor component (note contribution from **1** at peaks at 5.1, 1.5 and 1.3 ppm).

**1**											
Bond valence for Ti(IV)					Bond valence for Ti(III)						
Ti1											
r	r0-r	(r0-r)/B	e((ro-r)/B)		r	r0-r	(r0-r)/B	e((ro-r)/B)			
1.77	0.045	0.121622	1.129327		1.77	0.021	0.056757	1.058398331			
2.07	-0.255	-0.68919	0.501983		2.07	-0.279	-0.75405	0.47045543			
1.89	-0.075	-0.2027	0.816521		1.89	-0.099	-0.26757	0.765238624			
1.9	-0.085	-0.22973	0.794748		1.9	-0.109	-0.29459	0.744833486			
2.15	-0.335	-0.90541	0.404378		2.15	-0.359	-0.97027	0.378980597			
2.06	-0.245	-0.66216	0.515735		2.06	-0.269	-0.72703	0.483343824			
			**4.162692**					**3.901250291**		**good fit for Ti(IV)**	
Ti2											
r	r0-r	(r0-r)/B	e((ro-r)/B)		r	r0-r	(r0-r)/B	e((ro-r)/B)			
1.78	0.035	0.094595	1.099213		1.78	0.011	0.02973	1.03017607			
2.05	-0.235	-0.63514	0.529864		2.05	-0.259	-0.7	0.496585304			
1.88	-0.065	-0.17568	0.83889		1.88	-0.089	-0.24054	0.786202772			
1.9	-0.085	-0.22973	0.794748		1.9	-0.109	-0.29459	0.744833486			
2.15	-0.335	-0.90541	0.404378		2.15	-0.359	-0.97027	0.378980597			
2.08	-0.265	-0.71622	0.488598		2.08	-0.289	-0.78108	0.457910705			
			**4.155691**					**3.894688934**		**good fit for Ti(IV)**	
Ti3											
r	r0-r	(r0-r)/B	e((ro-r)/B)		r	r0-r	(r0-r)/B	e((ro-r)/B)			
2.07	-0.255	-0.68919	0.501983		2.07	-0.279	-0.75405	0.47045543			
2.04	-0.225	-0.60811	0.54438		2.04	-0.249	-0.67297	0.510189541			
1.78	0.035	0.094595	1.099213		1.78	0.011	0.02973	1.03017607			
1.93	-0.115	-0.31081	0.732853		1.93	-0.139	-0.37568	0.686825051			
2.16	-0.345	-0.93243	0.393595		2.16	-0.369	-0.9973	0.368875055			
1.89	-0.075	-0.2027	0.816521		1.89	-0.099	-0.26757	0.765238624			
			**4.088544**					**3.831759771**		**good fit for Ti(IV)**	

Table S1. Bond valence sum calculation for **1**. For Ti(iv)–O, r_o = 1.815; for Ti(iii)–O r_o = 1.791 and B = 0.37.[19]

Bond valence for Ti(IV)					Bond valence for Ti(III)				
Ti1									
r	r0-r	(r0-r)/B	e((ro-r)/B)		r	r0-r	(r0-r)/B	e((ro-r)/B)	
1.8	0.015	0.040541	1.041374		1.8	-0.009	-0.02432	0.975969	
2.169	-0.354	-0.95676	0.384137		2.169	-0.378	-1.02162	0.360011	
2.068	-0.253	-0.68378	0.504704		2.068	-0.277	-0.74865	0.473005	
2.081	-0.266	-0.71892	0.487279		2.081	-0.29	-0.78378	0.456675	
1.88	-0.065	-0.17568	0.83889		1.88	-0.089	-0.24054	0.786203	
1.879	-0.064	-0.17297	0.84116		1.879	-0.088	-0.23784	0.788331	
			**4.097543**					**3.840193**	**good fit for Ti(IV)**
Ti2									
r	r0-r	(r0-r)/B	e((ro-r)/B)		r	r0-r	(r0-r)/B	e((ro-r)/B)	
1.956	-0.141	-0.38108	0.683122		1.956	-0.165	-0.44595	0.640218	
2.083	-0.268	-0.72432	0.484652		2.083	-0.292	-0.78919	0.454213	
2.076	-0.261	-0.70541	0.493908		2.076	-0.285	-0.77027	0.462888	
1.93	-0.115	-0.31081	0.732853		1.93	-0.139	-0.37568	0.686825	
1.922	-0.107	-0.28919	0.748871		1.922	-0.131	-0.35405	0.701837	
2.073	-0.258	-0.6973	0.497929		2.073	-0.282	-0.76216	0.466656	
			**3.641335**					**3.412638**	**good fit for part valence Ti(III)/Ti(IV)**
Ti3									
r	r0-r	(r0-r)/B	e((ro-r)/B)		r	r0-r	(r0-r)/B	e((ro-r)/B)	
1.943	-0.128	-0.34595	0.707551		1.943	-0.152	-0.41081	0.663112	
2.081	-0.266	-0.71892	0.487279		2.081	-0.29	-0.78378	0.456675	
2.071	-0.256	-0.69189	0.500628		2.071	-0.28	-0.75676	0.469186	
1.91	-0.095	-0.25676	0.773556		1.91	-0.119	-0.32162	0.724972	
1.929	-0.114	-0.30811	0.734836		1.929	-0.138	-0.37297	0.688684	
2.08	-0.265	-0.71622	0.488598		2.08	-0.289	-0.78108	0.457911	
			**3.692447**					**3.46054**	**good fit for part valence Ti(III)/Ti(IV)**

Table S2. Bond valence sum calculation for **2**. For Ti(iv)–O, r_o = 1.815; for Ti(iii)–O, r_o = 1.791 and B = 0.37.[19]

**3**											
Bond valence for Ti(IV)					Bond valence for Ti(III)						
Ti1											
r	r0-r	(r0-r)/B	e((ro-r)/B)		r	r0-r	(r0-r)/B	e((ro-r)/B)			
2.04	-0.225	-0.60811	0.54438		2.04	-0.249	-0.67297	0.51019			
2.073	-0.258	-0.6973	0.497929		2.073	-0.282	-0.76216	0.466656			
2.068	-0.253	-0.68378	0.504704		2.068	-0.277	-0.74865	0.473005			
1.991	-0.176	-0.47568	0.621465		1.991	-0.2	-0.54054	0.582433			
1.958	-0.143	-0.38649	0.67944		1.958	-0.167	-0.45135	0.636767			
2.213	-0.283	-0.76486	0.465397	N	2.213	-0.283	-0.76486	0.465397			
			**3.313314**					**3.134448**		**good fit for Ti(III)**	
Ti2											
r	r0-r	(r0-r)/B	e((ro-r)/B)		r	r0-r	(r0-r)/B	e((ro-r)/B)			
1.869	-0.054	-0.14595	0.864204		1.869	-0.078	-0.21081	0.809927			
2.168	-0.353	-0.95405	0.385176		2.168	-0.377	-1.01892	0.360985			
2.076	-0.261	-0.70541	0.493908		2.076	-0.285	-0.77027	0.462888			
1.885	-0.07	-0.18919	0.82763		1.885	-0.094	-0.25405	0.77565			
2.081	-0.266	-0.71892	0.487279		2.081	-0.29	-0.78378	0.456675			
1.799	0.016	0.043243	1.044192		1.799	-0.008	-0.02162	0.97861			
			**4.10239**					**3.844735**		**good fit for Ti(IV)**	
Ti3											
r	r0-r	(r0-r)/B	e((ro-r)/B)		r	r0-r	(r0-r)/B	e((ro-r)/B)			
1.857	-0.042	-0.11351	0.892692		1.943	-0.152	-0.41081	0.663112			
2.125	-0.31	-0.83784	0.432645		2.081	-0.29	-0.78378	0.456675			
2.074	-0.259	-0.7	0.496585		2.071	-0.28	-0.75676	0.469186			
2.077	-0.262	-0.70811	0.492575		1.91	-0.119	-0.32162	0.724972			
1.92	-0.105	-0.28378	0.752929		1.929	-0.138	-0.37297	0.688684			
1.792	0.023	0.062162	1.064135		2.08	-0.289	-0.78108	0.457911			
			**4.131562**					**3.46054**		**good fit for Ti(IV)**	

Table S3. Bond valence sum calculation for **3**. For Ti(iv)–O, r_o = 1.815; for Ti(iii)–O, r_o = 1.791; For Ti–N, r_o = 1.93 (same value used for both oxidation states, based on available data) and B = 0.37.[19] N.B. Ti1–N = 2.213 Å.

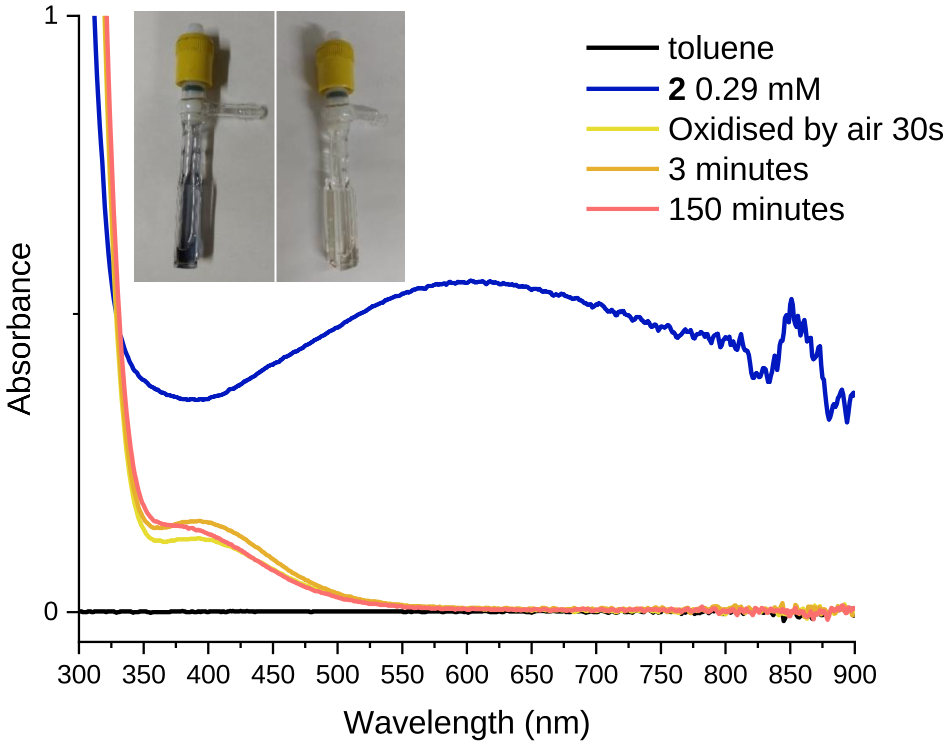

Fig S23. UV/vis spectra of **2** (0.29 mM, or 1.74 mM [Ti]) in toluene and subsequent spectrum after oxidation with air. Note that oxidized signal grows to a maximum after ~3 minutes after air addition, then slowly decreases as onward reactivity (back to **1**) occurs. Photos show colour before and after oxidation.

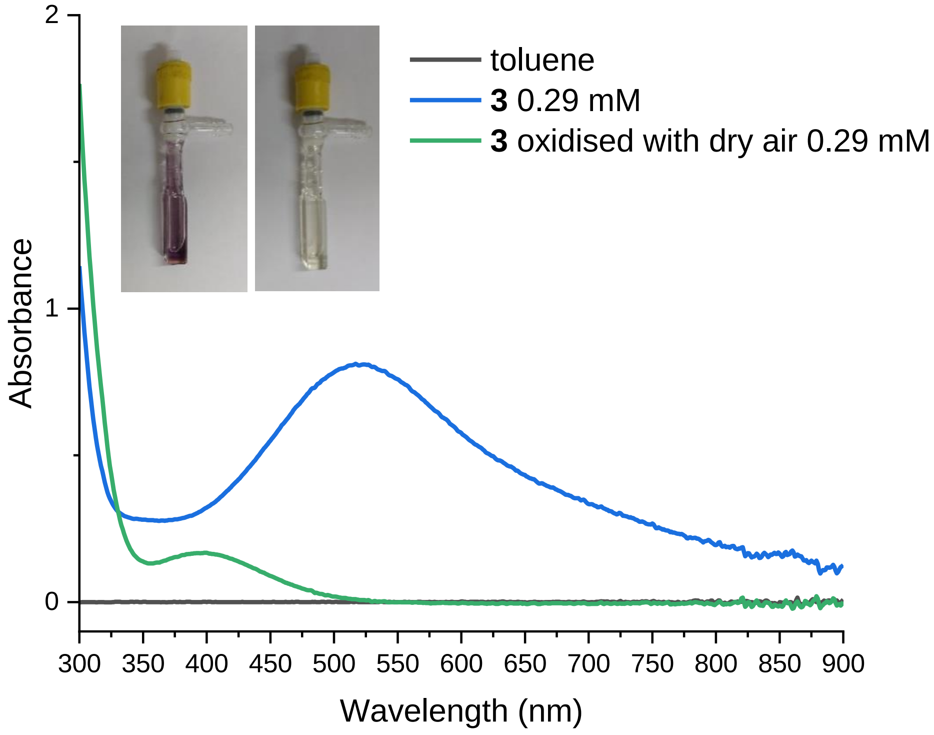

Fig S24. UV/vis spectra of **3** (0.29 mM, or 1.74 mM [Ti]) in toluene and subsequent spectrum after oxidation with dry air. Photos show colour before and after oxidation.

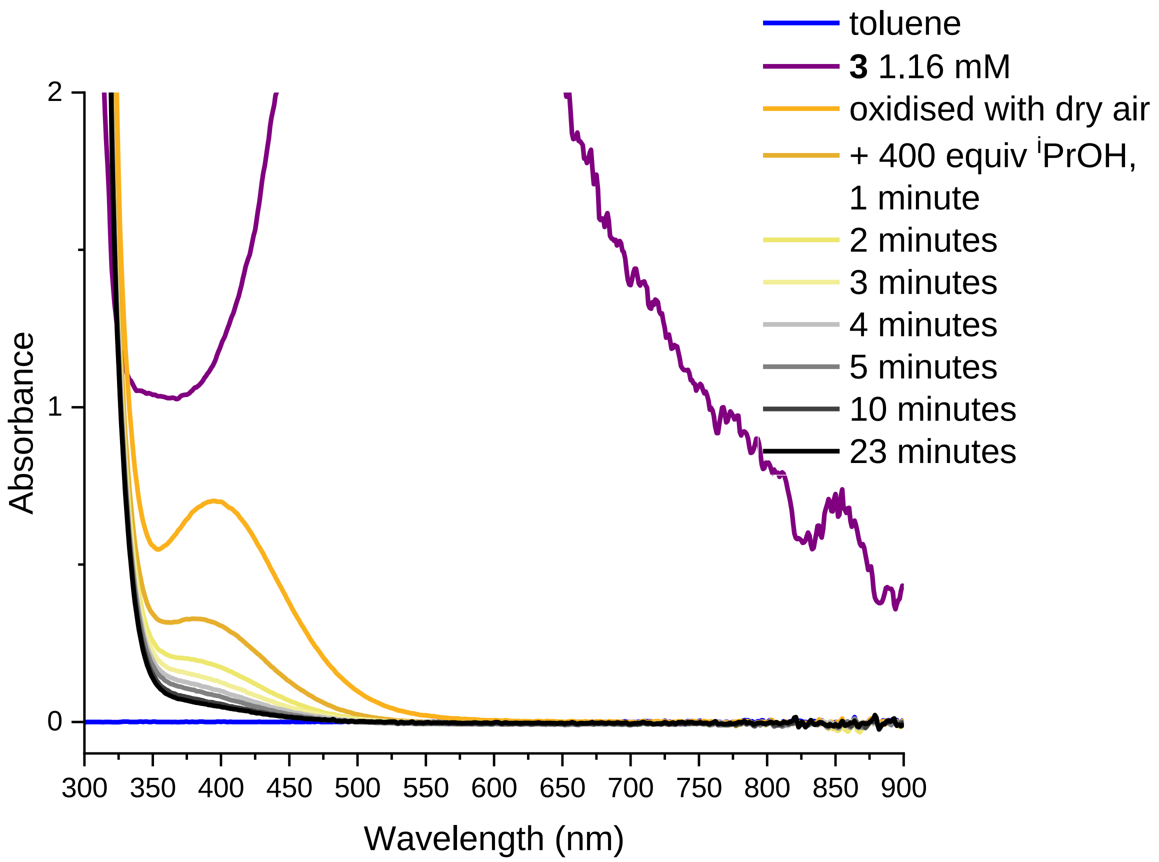

Fig S25. UV/vis spectra of **3** (1.16 mM, or 6.96 mM [Ti]) in toluene and subsequent spectrum after oxidation with dry air and then addition of 400 equiv iPrOH (concentration drops to 1.13 mM). Peak maximum after oxidation is 395 nm. Approximate first order decay of 395 nm signal is observed, although a longer lived shoulder of the major OMCT band of **1** remains after the reoxidation process.

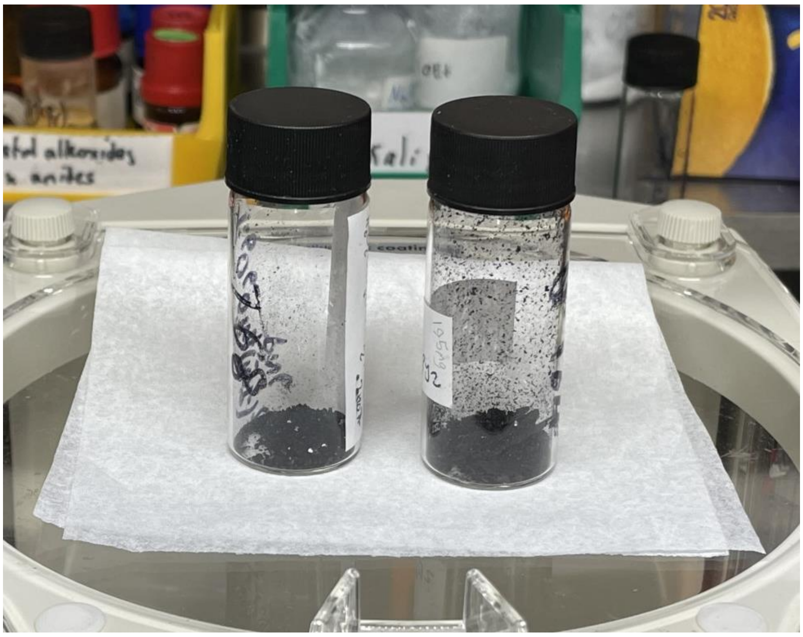

Fig S26. Photograph of **2** (left) and **3** (right) as isolated crystalline material in vials within a N_2 filled glovebox.

a)

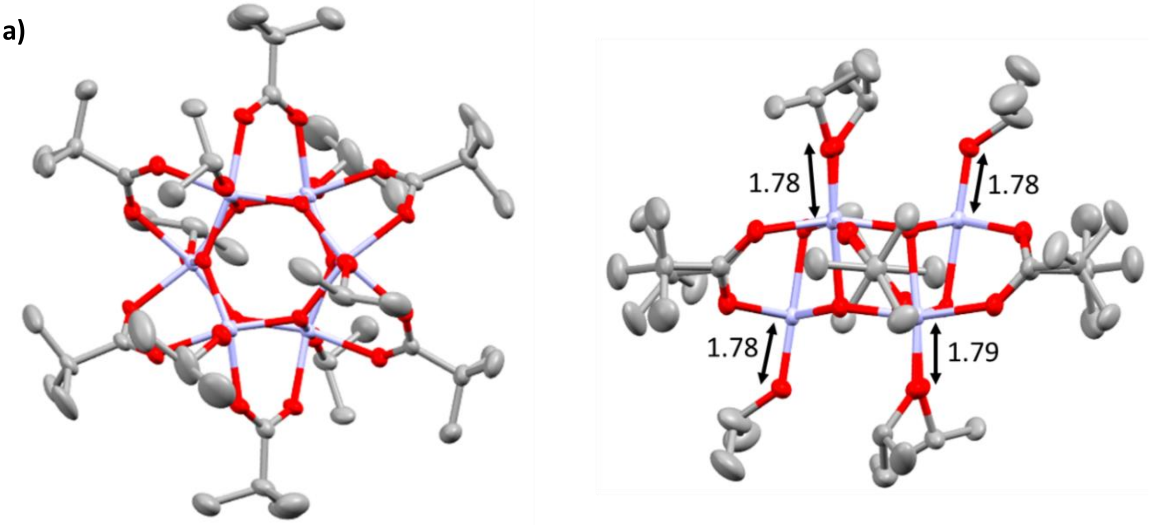

b)

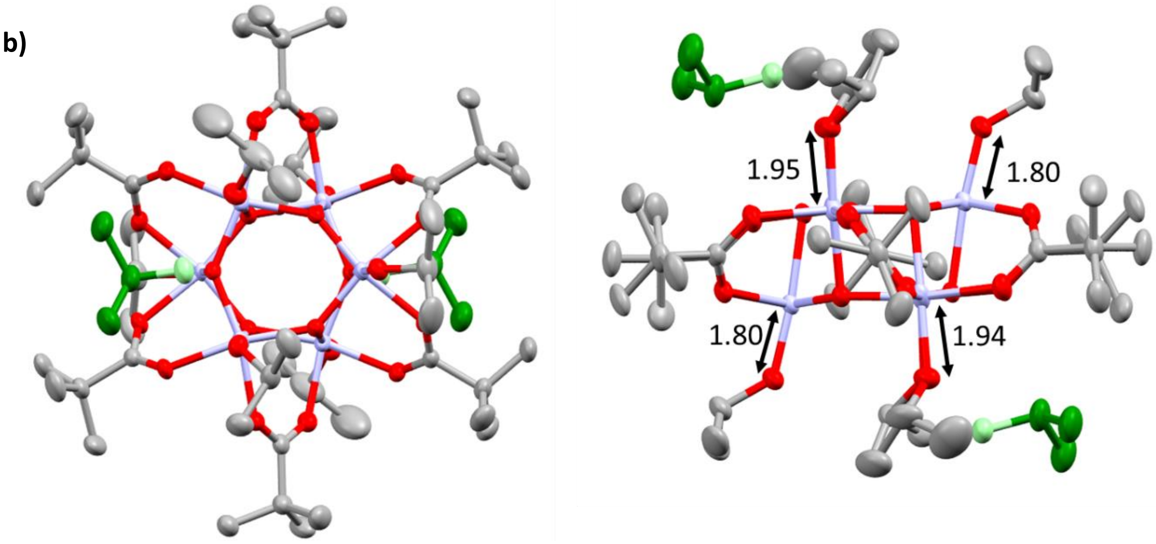

c)

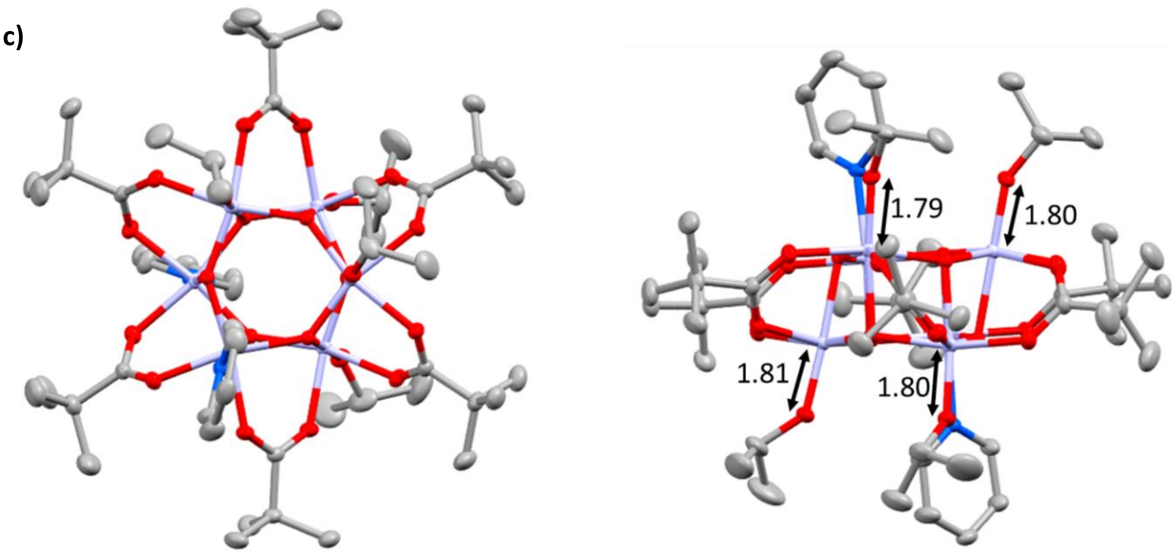

Fig S27. Structures of **1-3** shown from two different angles, ellipsoids at 50% probability and hydrogen atoms omitted for clarity. a) One of two molecules in asymmetric unit of **1** shown. b) Toluene solvent molecule not shown, isopropanol solvent molecules shown in green. c) Toluene solvent molecule not shown.

Table S4. Bond lengths (in Å) and angles (in °). Largest and shortest across series highlighted in bold. **2** has two shorter cross hexagon Ti-O lengths and two larger 'in hexagon' angles, and greater variation in Ti-O-Ti 'between hexagon' angles. **3** has a bigger range of Ti-O lengths and wide range of Ti-O-Ti angles. **5** is the most distorted with a wide range of lengths and angles.

	**1**	**2**	**3**	**5**
Ti–O^{i}Pr<br>*Ti–(H)O^{i}Pr*	1.773(2)-1.785(2)	1.801(3),<br>*1.941(3), 1.953(3)*	1.792(2)-**1.806(2)**	**1.764(6)**-1.782(6)
Ti–O$_2$CCMe$_3$	2.037(2)-2.080(2)	2.069(2)-2.081(2)	2.062(2)-**2.098(2)**	**2.0275(5)**-2.092(5)
Ti–O (hexagonal plane)	1.879(2)-1.928(2)	1.879(2)-1.929(2)	1.839(2)-1.995(2)	**1.817(5)-2.031(5)**
Ti–O (between hexagons)	2.140(2)-2.161(2)	2.082(3)-2.170(3)	2.040(2)-2.169(2)	**1.926(6)-2.551(6)**
Ti–O–Ti (hexagonal plane)	133.8(1)-135.5(1)	135.9(1)-139.0(1)	133.4(1)-**143.2(1)**	**133.3(2)**-138.5(3)
Ti–O–Ti (between hexagons)	99.37(9)-101.3(1)	93.6(1)-100.1(1)	**85.09(8)**-<br>100.87(9)	90.6(2)-**107.8(2)**
Ti–N			2.212(2)-**2.213(2)**	**2.166(5)**

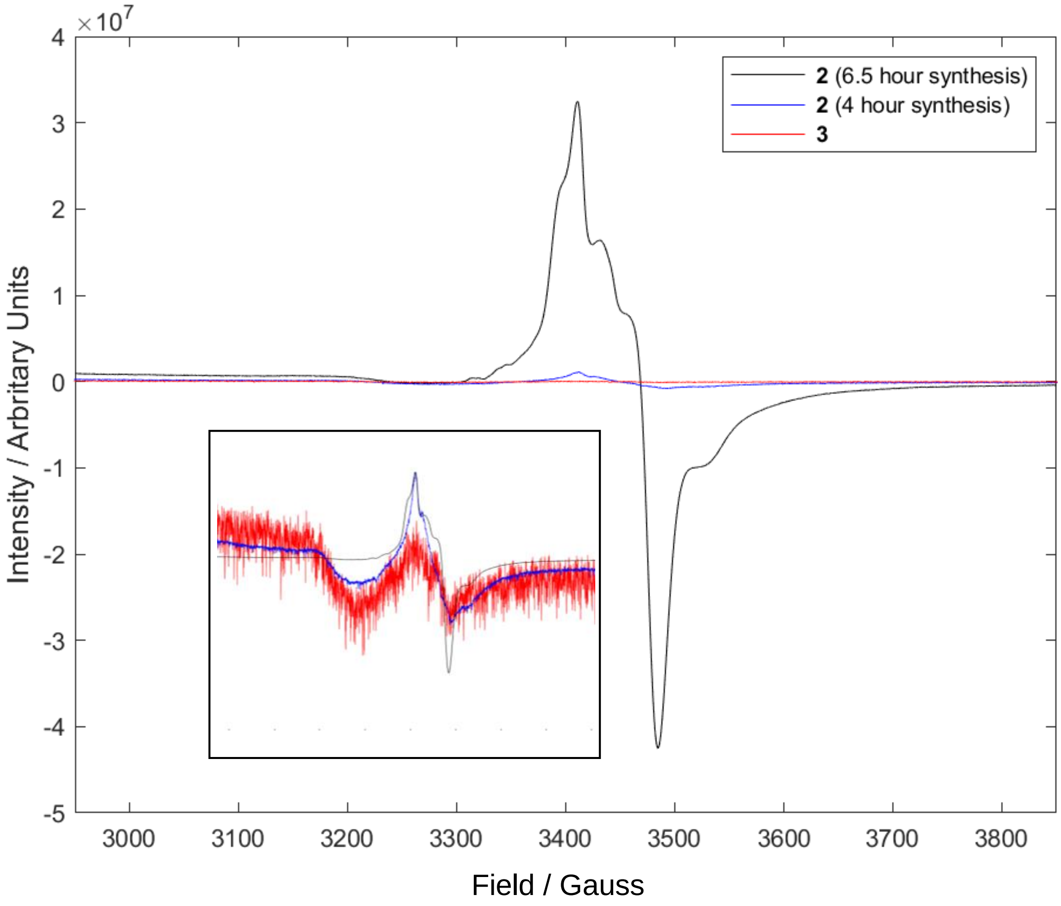

Figure S28. Low-temperature (100 K) EPR spectra of isolated **2** or **3** (0.25 mM, frozen toluene solution). Two batches of **2** are displayed, one synthesised by 4 hours UV irradiation, the other by 6.5 hours irradiation. The longer irradiation leads to an increased quantity of a minor paramagnetic by-product from the onward photoreaction of **2**. The EPR signal from the paramagnetic by-product is indicative of a Ti(III) species. The signal is consistent with other reported Ti(III) systems.[20] The inset shows the peak region scaled with arbitrary weights to approximate signal intensity, showing a similar paramagnetic signal is present in all three spectra, but with different intensity. Solid-state EPR spectra (120 K) of **2** gave similar spectra.

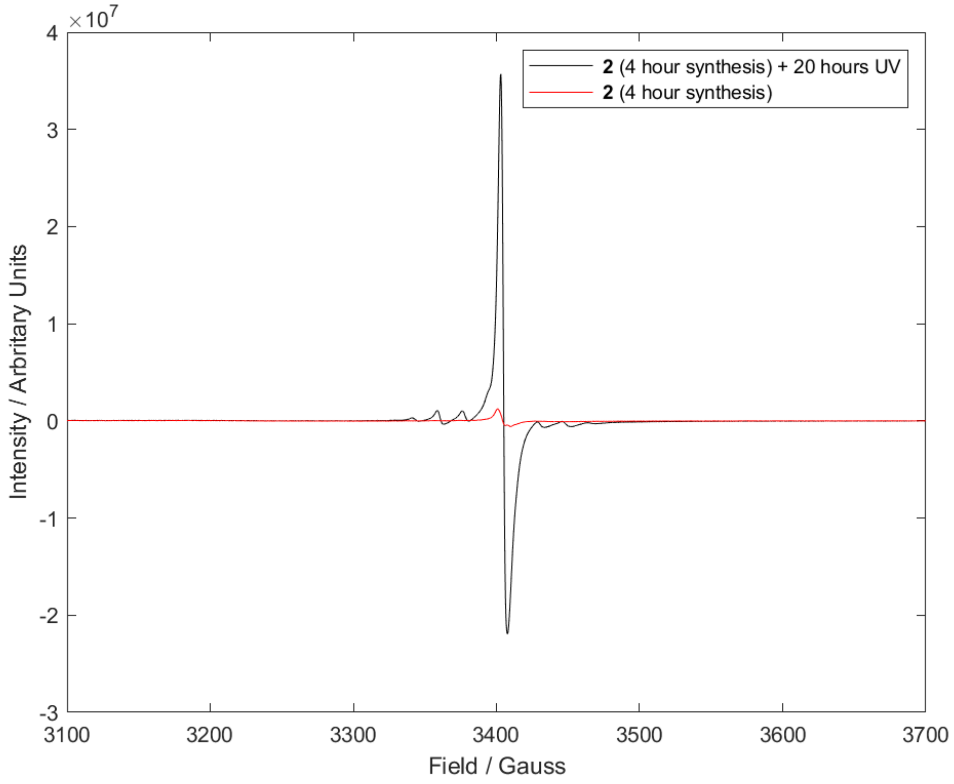

Fig S29. X-band spectra of isolated **2** (synthesized by 4 hours irradiation, 2.5 mM, toluene solution – which gives **2** with negligible paramagnetic by-product) at room-temperature (291 K) before (red) and after (black) irradiating the quartz tube with 302 nm UV light for a further 20 hours. The paramagnetic signal is approximately 30 times stronger after the extra 20 hours irradiation. Comparisons were conducted using exactly the same spectrometer settings (mw power, time constant, conversion time, modulation frequency, modulation amplitude).

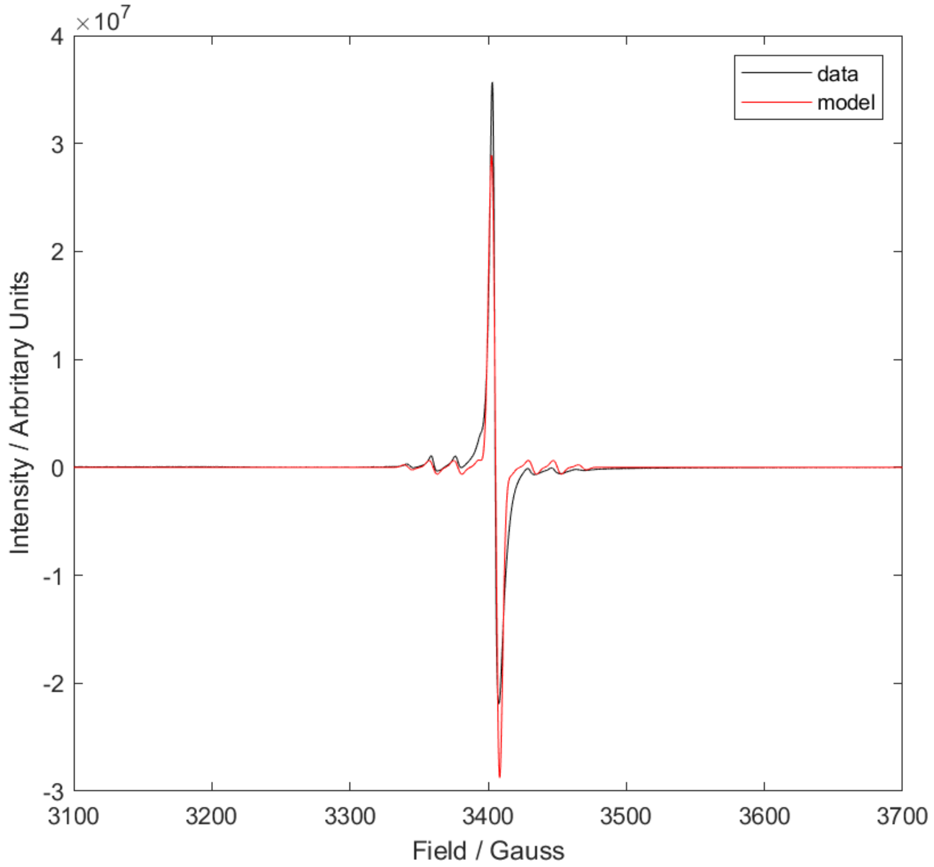

Fig S30. Experimental (black) and simulated (red) X-band spectra of a 2.5 mM, toluene solution of **2** which has been further irradiated for an extra 20 hours to generate a significant signal from the paramagnetic by-product. Modelled with g = 1.96, A = 0.00164 cm^{-1} and a Gaussian line broadening of 5.9 G. The spectrum shows clear hyperfine coupling to Ti(III) suggesting the unpaired electrons in the minor paramagnetic impurity are localised on Ti(III).

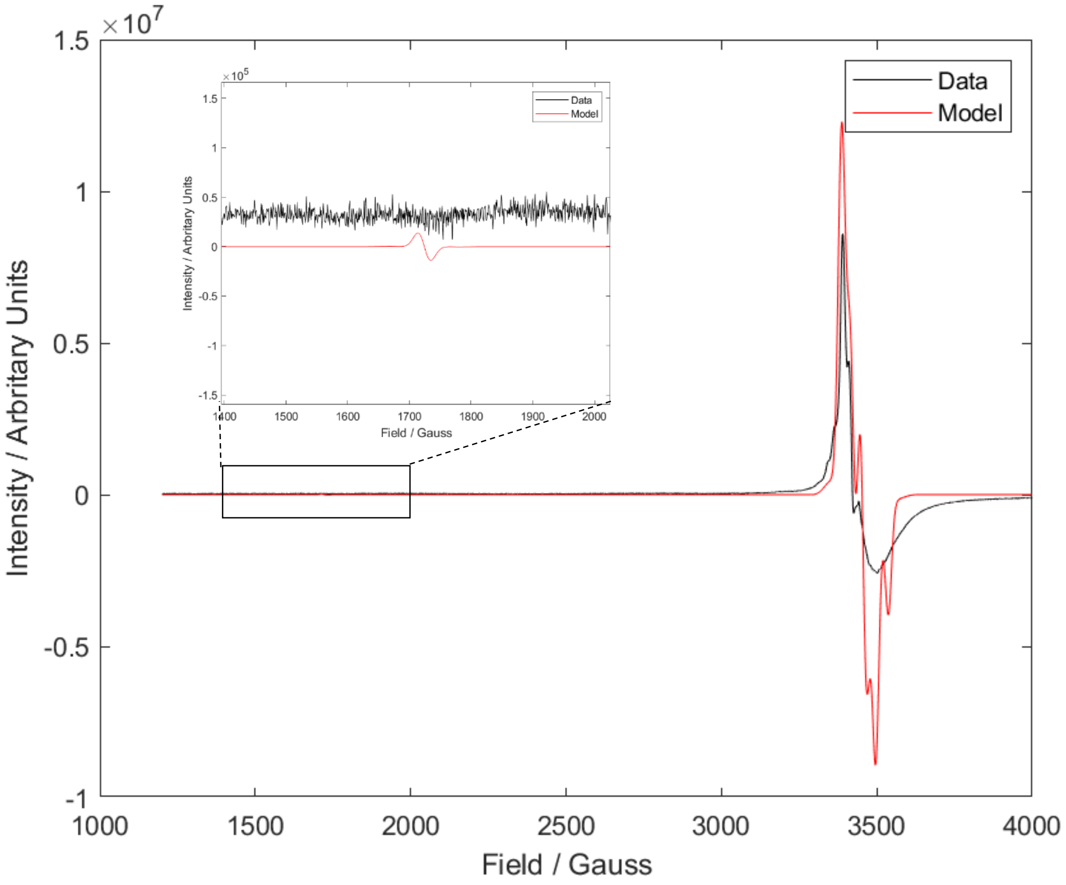

Fig S31. Experimental (black) and simulated (red) X-band spectrum of **2** (2.5 mM frozen toluene solution) which has been irradiated with 302 nm light for an extra 20 hours at 100K to give a measurable quantity of paramagnetic by-product. A Gaussian peak broadening model was used with a FWHM of 20 G. The simulation gives values of $g_{\parallel}$ = 1.93, $g_{\perp}$ = 1.92, D = 0.0066 cm^{-1}, and E = 0.0012 cm^{-1} for the paramagnetic by-product. Inset shows zoomed section displaying the region in which a very weak half-field transition is predicted for the formally forbidden Δm_s = ± 2 transition in a S=1 species, however, identification of this signal difficult as it is very weak.

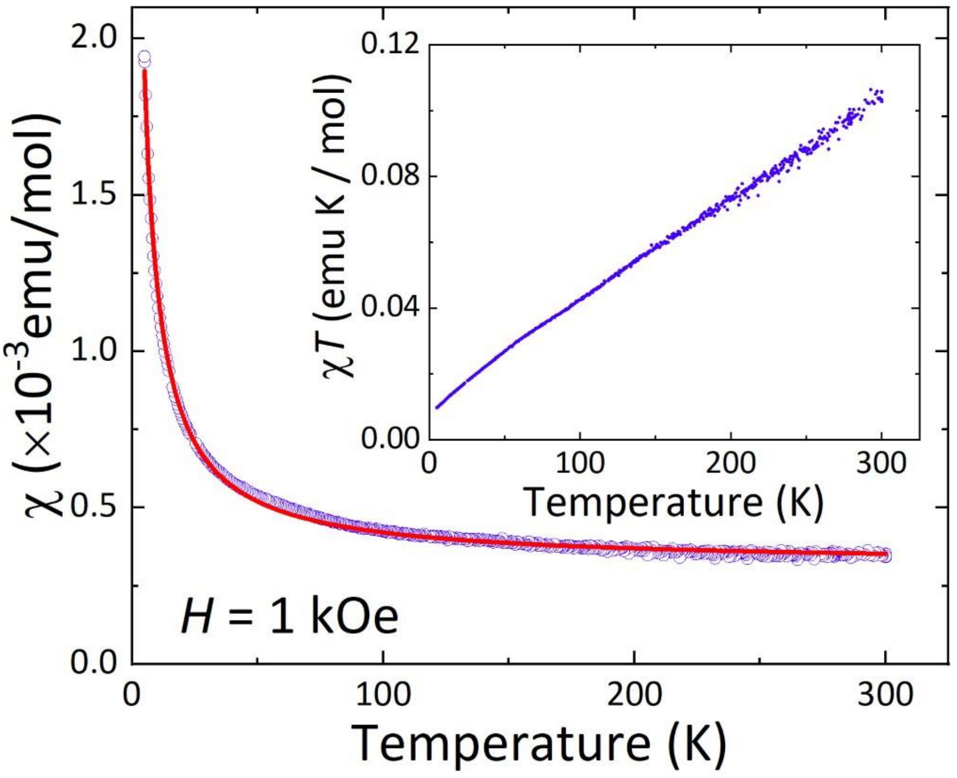

Figure S32. Temperature dependence of the magnetic susceptibility of **2** (batch prepared by 6 hours irradiation – which yields a small amount of paramagnetic by-product as observed by EPR spectroscopy), measured from 5 to 300 K in an applied field of 1 kOe. The signal is weakly paramagnetic. χT for **2** at 300 K is ~0.1 emu K/mol, well below the expected value (0.75 emu K/mol) for two uncorrelated spins or (1 emu K/mol) for an S=1 state.[21] Fitting to a Curie-Weiss law (shown with red line) [χ(T) = C/(T - θW) + χ0] gives χ0 = +3.2(1) × 10-4 emu/mol and θW = -1.6(1) K suggesting weak antiferromagnetic interactions. Using g = 2 suggests ~1% of the molecules are in a triplet (S = 1) Ti(III) state, consistent with the signal arising from a minor by-product. The inset shows the temperature dependence of χT, this may be explained by a temperature independent paramagnetic (TIP) contribution which dominates the small temperature dependent paramagnetism at high temperatures.

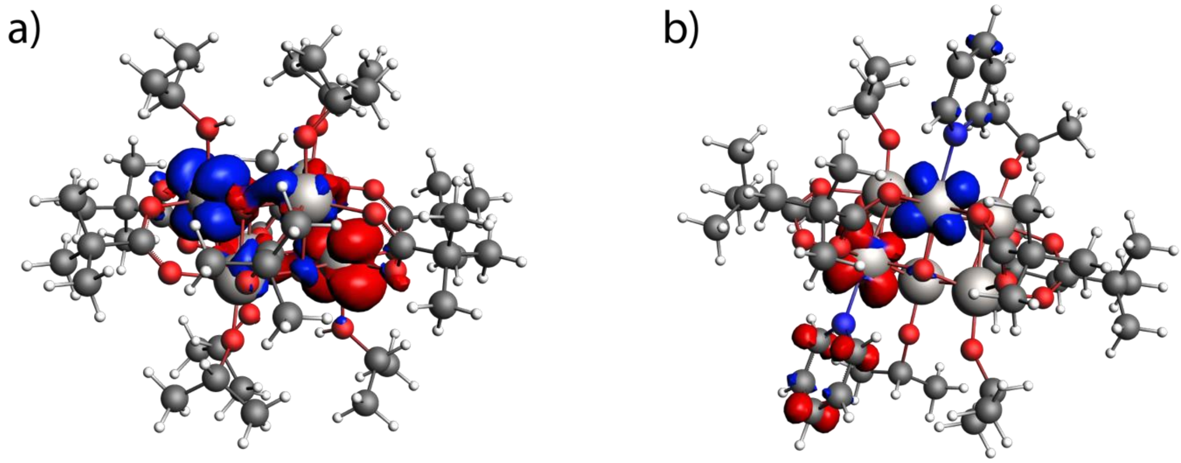

Fig S33. Spin density for broken symmetry a) **2** and b) **3**.

Supporting note 1. Examination of any metal-metal bonding interactions

The broken-symmetry solutions for **2** and **3** were further analysed for signs of bonding interactions between the two Ti(III) centres. For both compounds the expectation value of S^2 is very close to 1.0 (0.983 for **2**, and 1.000 for **3**), indicating that the overlap between the singly occupied orbitals is very close to 0.0, suggesting the absence of bonding interactions. This is further corroborated with a quantum theory of atoms in molecules (QTAIM) analysis,[22, 23] which showed that no bond critical point could be located between the two Ti atoms. Hence, no sign of bonding interactions between the two singly occupied Ti d-orbitals was found.

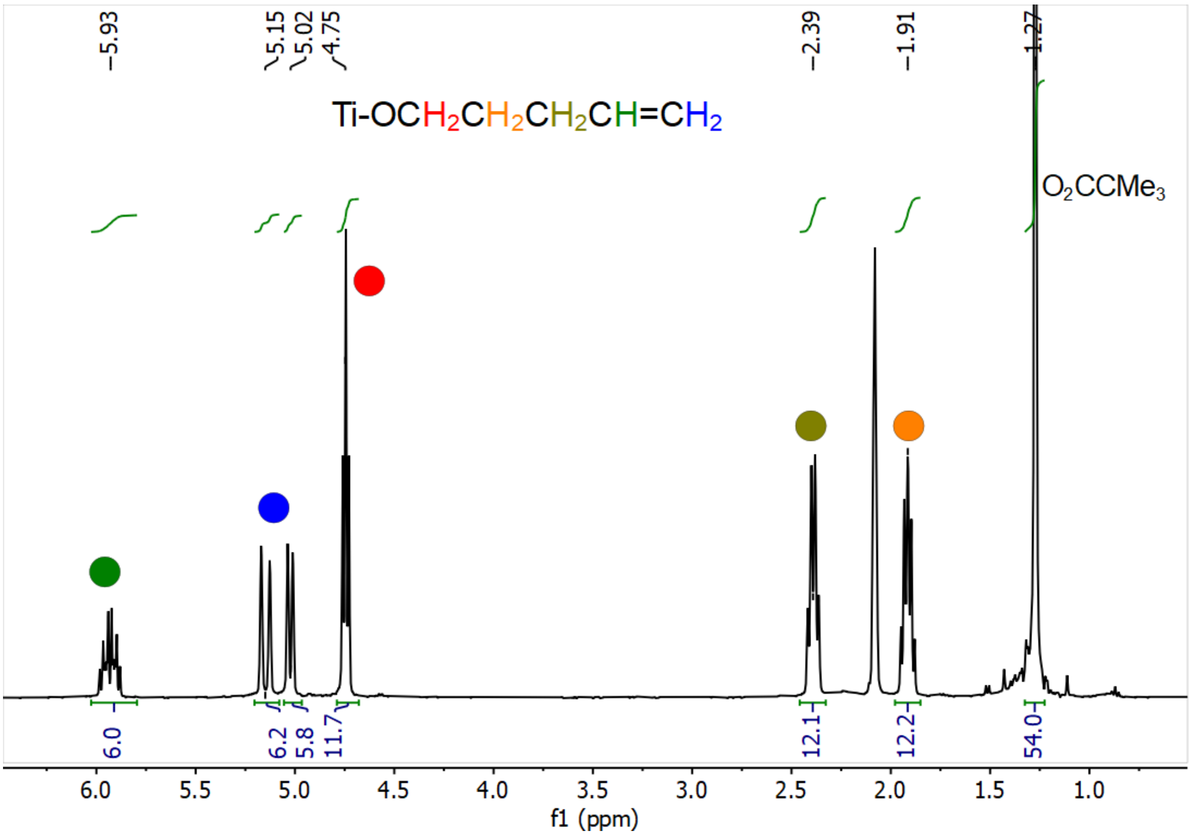

Fig S34. ^{1}H NMR spectrum of **1*** (d8-toluene)

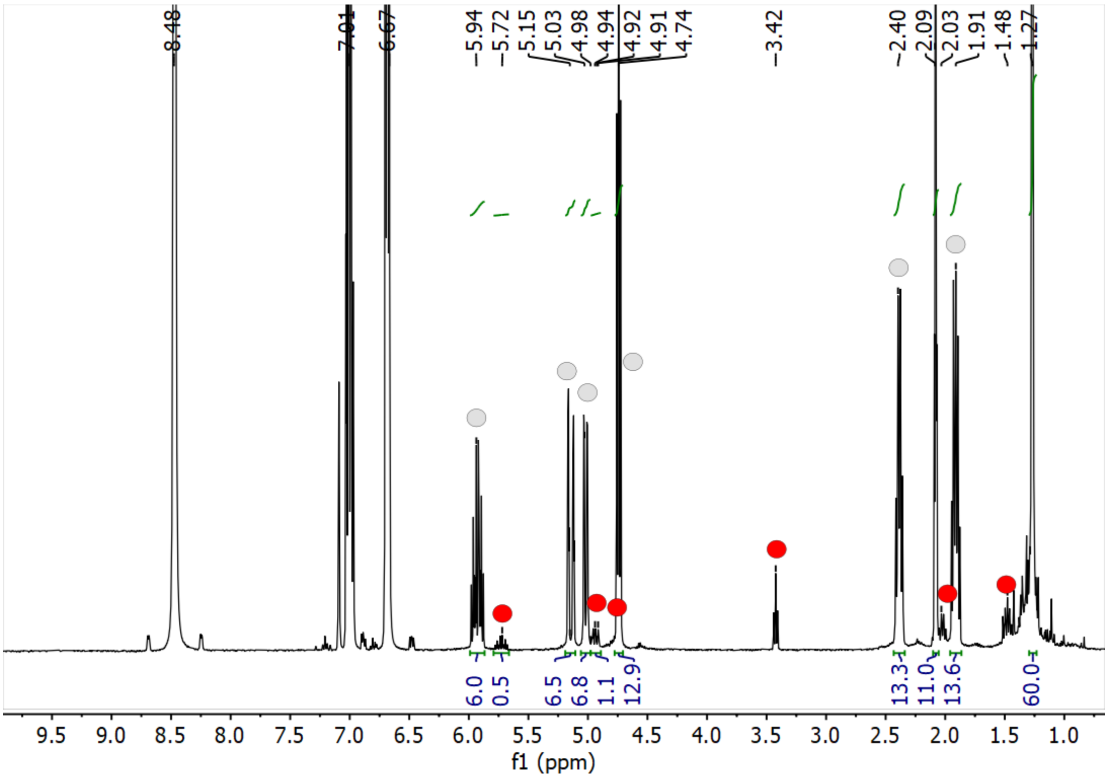

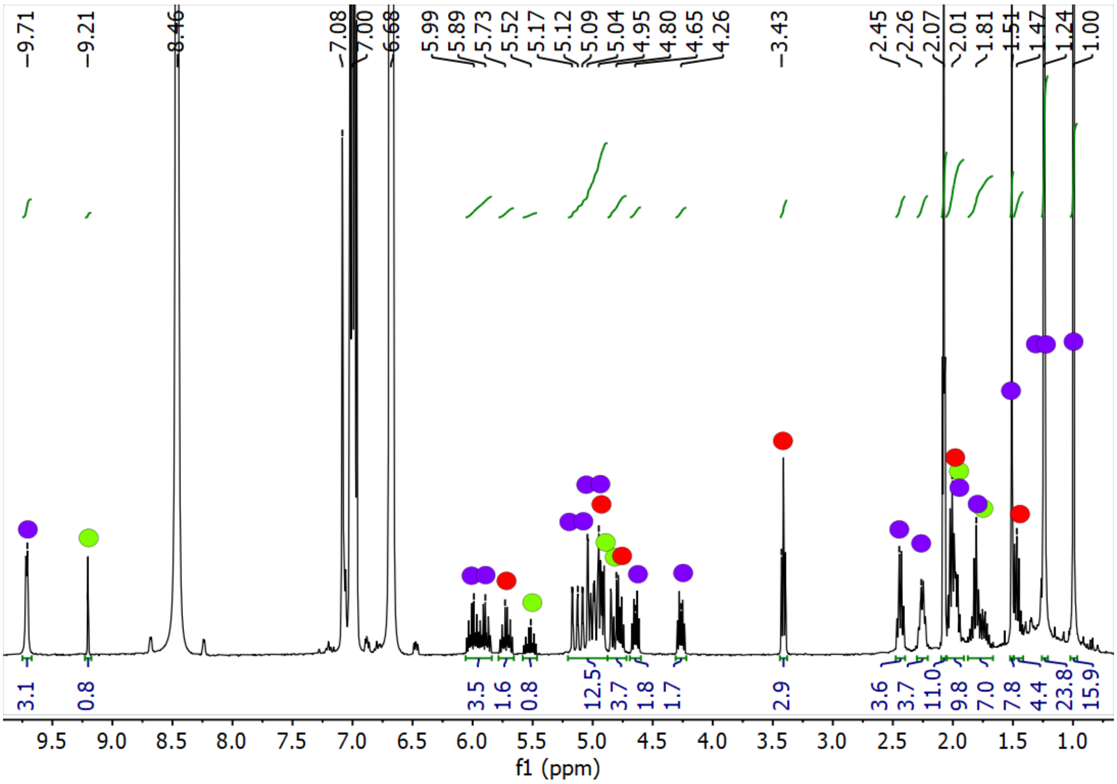

Fig S35. ^{1}H NMR spectra of **1*** + 30 equiv. pyridine before and after irradiation with UV light for 2 hours. Grey = **1***, purple = **3***, red = 4-penten-1-ol, green = pentenal (peak identification aided by using the corresponding ^{1}H-COSY 2D-NMR spectrum). N.B. some trace 4-penten-1-ol was found in the

sample of **1*** after its preparation (see red spots on 'before' spectrum). After irradiation for two hours all **1*** was consumed and replaced by an approximately 1:1:1 ratio of **3***, 4-penten-1-ol and pentenal (see below). No unidentified signals identified in the 3-4.2 ppm region where cyclized products would be expected.

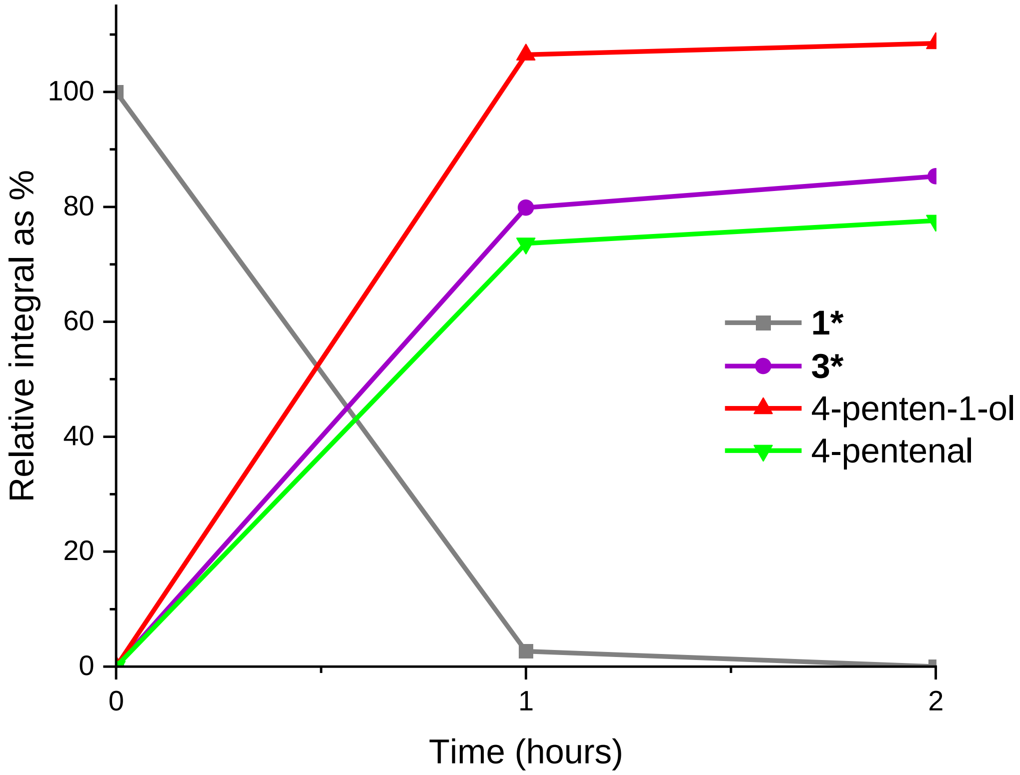

Fig S36. Products formed during photoirradiation of **1***

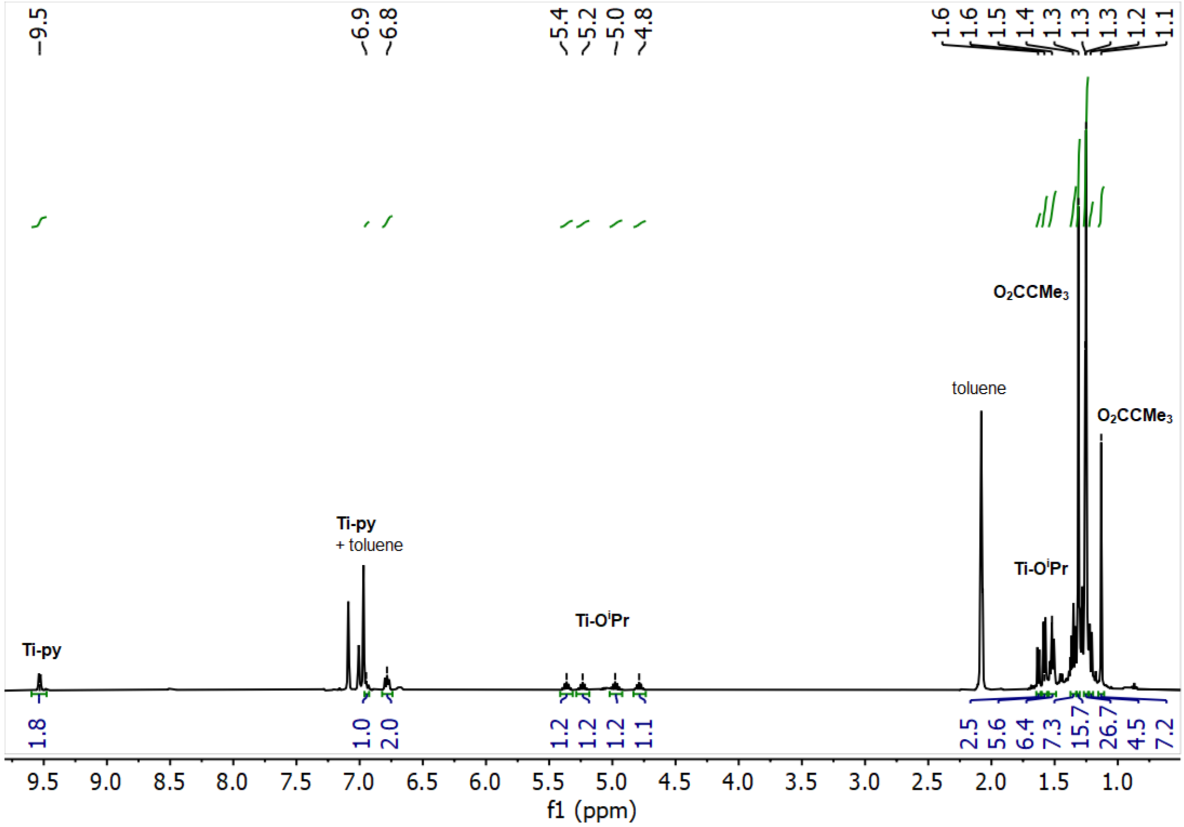

Fig S37. ^{1}H NMR spectra of **5** in d^8-toluene.

Table S5. Bond valence sum calculation for **5**. For Ti(iv)–O, r_o = 1.815; for Ti(iii)–O, r_o = 1.791; For Ti–N, r_o = 1.93 (same value used for both oxidation states, based on available data) and B = 0.37.[19] N.B. Ti1–N = 2.166 Å.

**5**										
Bond valence for Ti(IV)					Bond valence for Ti(III)					
Ti2 (pyridine)										
r	r0-r	(r0-r)/B	e((ro-r)/B)		r	r0-r	(r0-r)/B	e((ro-r)/B)		
1.926	-0.111	-0.3	0.740818		1.926	-0.135	-0.36486	0.69429		
1.817	-0.002	-0.00541	0.994609		1.817	-0.026	-0.07027	0.932142		
2.05	-0.235	-0.63514	0.529864		2.05	-0.259	-0.7	0.496585		
2.075	-0.26	-0.7027	0.495245		2.075	-0.284	-0.76757	0.464141		
1.848	-0.033	-0.08919	0.914673		1.848	-0.057	-0.15405	0.857226		
2.166	-0.236	-0.63784	0.528434	N	2.166	-0.236	-0.63784	0.528434	N	
			**4.203643**					**3.972818**		**good fit for Ti(III)**
Ti1 (O2)										
r	r0-r	(r0-r)/B	e((ro-r)/B)		r	r0-r	(r0-r)/B	e((ro-r)/B)		
2.551	-0.736	-1.98919	0.136806		2.551	-0.76	-2.05405	0.128214		
2.031	-0.216	-0.58378	0.557784		2.031	-0.24	-0.64865	0.522752		
2.054	-0.239	-0.64595	0.524166		2.054	-0.263	-0.71081	0.491246		
1.931	-0.116	-0.31351	0.730875		1.931	-0.14	-0.37838	0.684971		
2.092	-0.277	-0.74865	0.473005		2.092	-0.301	-0.81351	0.443298		
1.83	-0.015	-0.04054	0.96027		1.83	-0.039	-0.10541	0.89996		
1.84	-0.025	-0.06757	0.934665		1.84	-0.049	-0.13243	0.875962		
			**4.317571**					**4.046402**		**good fit for Ti(IV)**
Ti3										
r	r0-r	(r0-r)/B	e((ro-r)/B)		r	r0-r	(r0-r)/B	e((ro-r)/B)		
2.188	-0.373	-1.00811	0.364909		2.188	-0.397	-1.07297	0.34199		
1.927	-0.112	-0.3027	0.738819		1.927	-0.136	-0.36757	0.692417		
2.07	-0.255	-0.68919	0.501983		2.07	-0.279	-0.75405	0.470455		
1.771	0.044	0.118919	1.126279		1.771	0.02	0.054054	1.055542		
2.034	-0.219	-0.59189	0.55328		2.034	-0.243	-0.65676	0.51853		
1.867	-0.052	-0.14054	0.868888		1.867	-0.076	-0.20541	0.814317		
			**4.154157**					**3.893251**		**good fit for Ti(IV)**
Ti4										
r	r0-r	(r0-r)/B	e((ro-r)/B)		r	r0-r	(r0-r)/B	e((ro-r)/B)		
1.764	0.051	0.137838	1.147789		1.764	0.027	0.072973	1.075701		
2.147	-0.332	-0.8973	0.40767		2.147	-0.356	-0.96216	0.382066		
2.036	-0.221	-0.5973	0.550297		2.036	-0.245	-0.66216	0.515735		
1.883	-0.068	-0.18378	0.832116		1.883	-0.092	-0.24865	0.779854		
1.923	-0.108	-0.29189	0.746849		1.923	-0.132	-0.35676	0.699943		
2.063	-0.248	-0.67027	0.51157		2.063	-0.272	-0.73514	0.479441		
			**4.196292**					**3.93274**		**good fit for Ti(IV)**
Ti5										
r	r0-r	(r0-r)/B	e((ro-r)/B)		r	r0-r	(r0-r)/B	e((ro-r)/B)		
2.132	-0.317	-0.85676	0.424537		2.132	-0.341	-0.92162	0.397873		
1.775	0.04	0.108108	1.114168		1.775	0.016	0.043243	1.044192		
1.918	-0.103	-0.27838	0.75701		1.918	-0.127	-0.34324	0.709466		
2.077	-0.262	-0.70811	0.492575		2.077	-0.286	-0.77297	0.461639		
2.034	-0.219	-0.59189	0.55328		2.034	-0.243	-0.65676	0.51853		
1.878	-0.063	-0.17027	0.843437		1.878	-0.087	-0.23514	0.790464		
			**4.185007**					**3.922164**		**good fit for Ti(IV)**
Ti6										
r	r0-r	(r0-r)/B	e((ro-r)/B)		r	r0-r	(r0-r)/B	e((ro-r)/B)		
1.782	0.033	0.089189	1.093287		1.782	0.009	0.024324	1.024623		
2.119	-0.304	-0.82162	0.439718		2.119	-0.328	-0.88649	0.412101		
2.027	-0.212	-0.57297	0.563847		2.027	-0.236	-0.63784	0.528434		
1.88	-0.065	-0.17568	0.83889		1.88	-0.089	-0.24054	0.786203		
1.905	-0.09	-0.24324	0.784081		1.905	-0.114	-0.30811	0.734836		
2.063	-0.248	-0.67027	0.51157		2.063	-0.272	-0.73514	0.479441		
			**4.231393**					**3.965637**		**good fit for Ti(IV)**

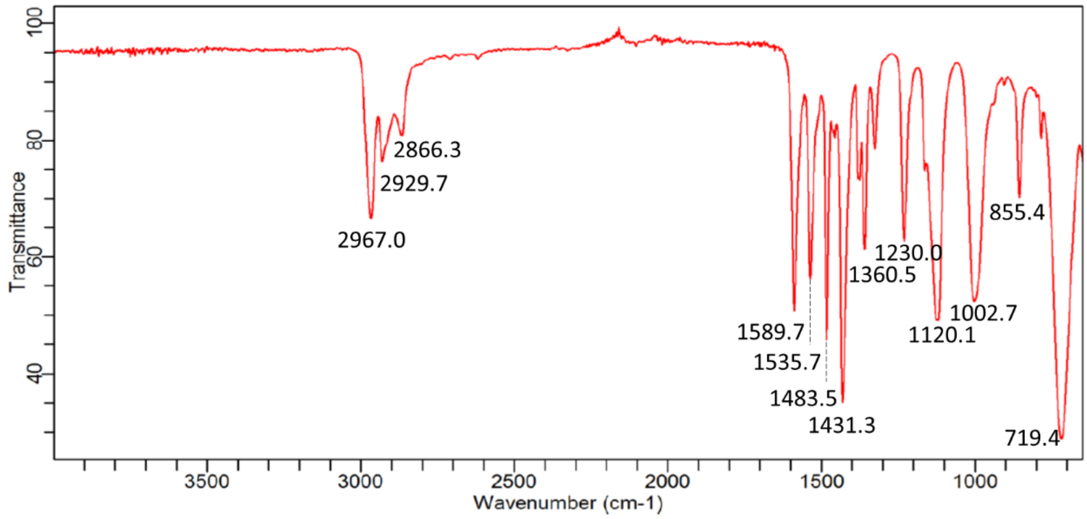

Fig S38. ATR-FTIR spectrum of **1**. N.B. Previous analysis by Piszczek et al.[15] identifies the stretches as the following:

- 2866-2967 = C–H stretches
- 1590 & 1535 = asymmetric COO stretch
- 1483.5 & 1431.3 = symmetric COO stretch
- *1360 & 1230 not assigned*
- 1120 = C–O stretching vibrations of alkoxide groups & $\rho_r(CH_3)$
- 1002 = coupled ν(CO), ν(Ti–O) vibration
- *855 not assigned*
- 719 = Ti–(μ-O) stretching modes

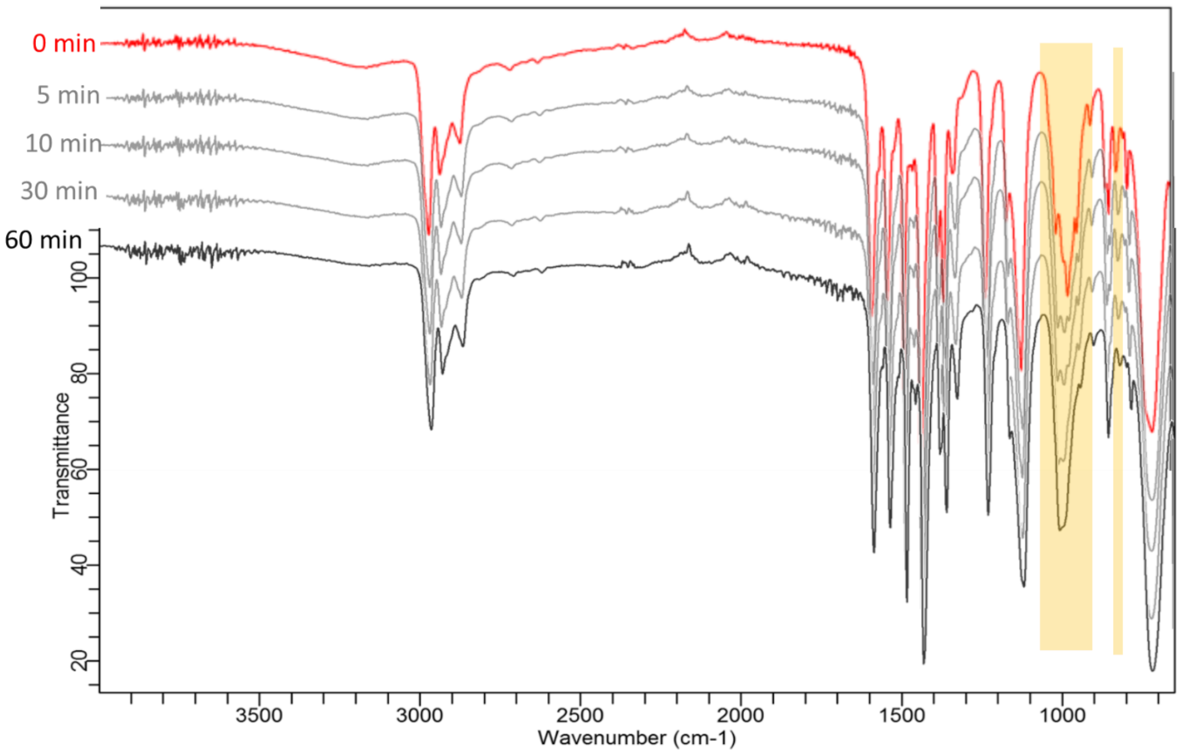

Fig S39. ATR-FTIR spectrum of **2** under air over time (exposed to air ~0.5 minutes before t=0 collection, some immediate oxidation may occur before data collection). Highlighted sections show some change in spectra over air exposure. Notable differences to the spectra of **1** include a broad signal at ~3200 cm^{-1} from the ^{i}PrO−H stretches in **2**; shifted and more defined signals ~1000 cm^{-1} (from coupled ν(CO), ν(Ti–O) vibration) which become similar to **1** after oxidation; and a minor unassigned signal at 875 cm^{-1} which weakens on oxidation.

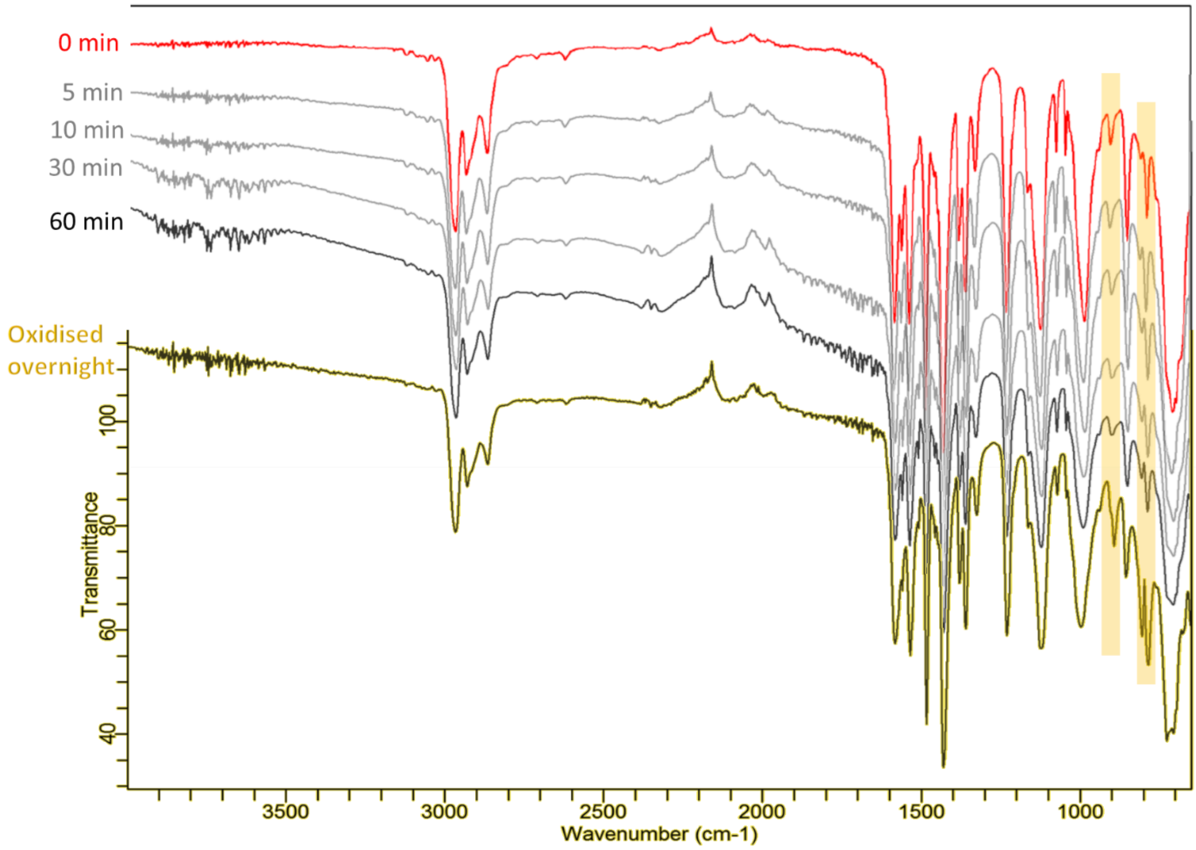

Fig S40. ATR-FTIR spectrum of **3** under air over time (exposed to air ~0.5 minutes before t=0 collection, some immediate oxidation may occur before data collection). Highlighted sections show some change in spectra over air exposure. Notable differences to the spectra of **1** include a minor unassigned signal at 1060 cm^{-1} which slightly weakens on oxidation; new peaks at 892 cm^{-1} and 805 cm^{-1} which appear only after complete oxidation of **3** (to **5**).

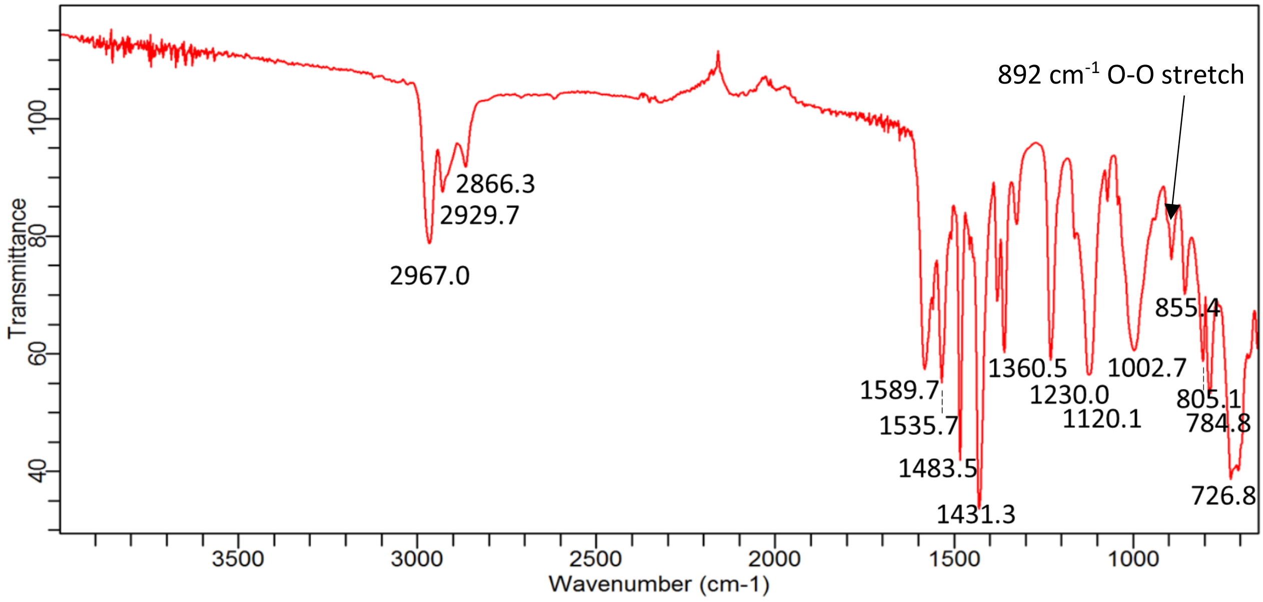

Fig S41. ATR-FTIR spectrum of fully oxidized **3** (i.e. compound **5).**

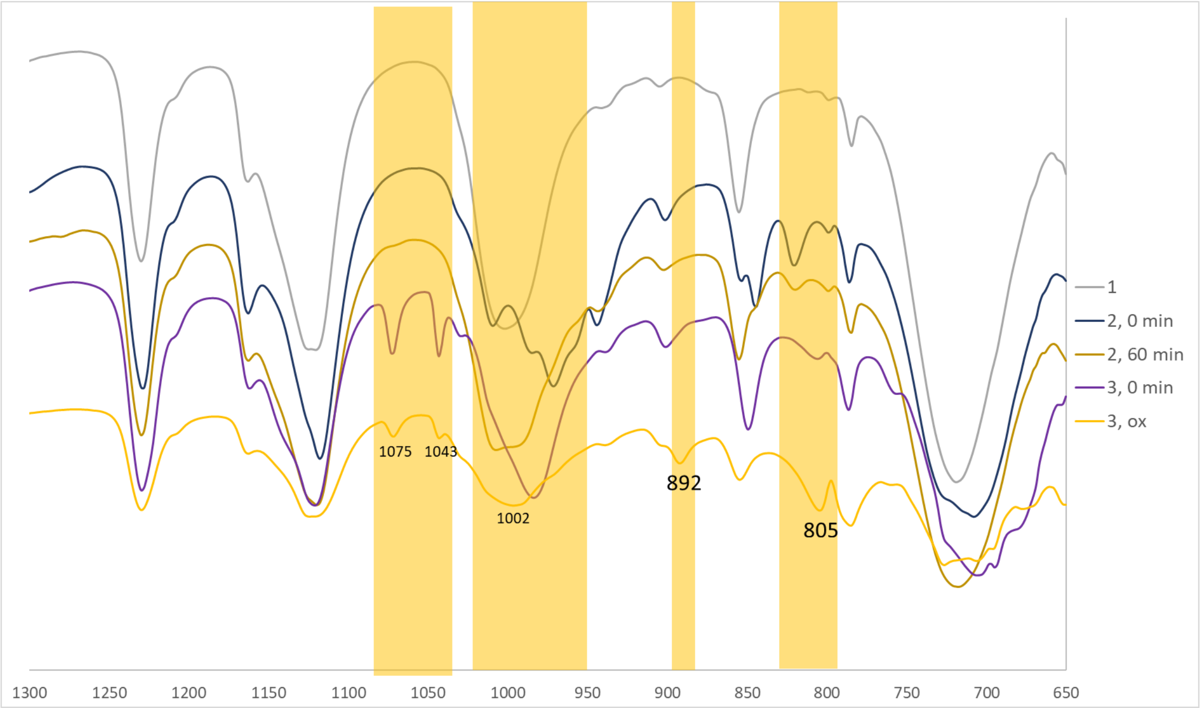

Fig S42. Zoomed section of ATR-FTIR spectrum of **1**, **2**, **2 oxidised (60 minutes)**, **3** and **3 oxidised (overnight, = 5)** highlighting differences between the spectra. Signal at 892 cm^{-1} is only clearly observed in the fully oxidized **3** (= **5**). The signal at 805 cm^{-1} is also much stronger in fully oxidized **3** but also present in **3.** Signals at 1075 and 1043 are only present in **3** and oxidized **3** (= **5**), which have bound pyridine ligands.

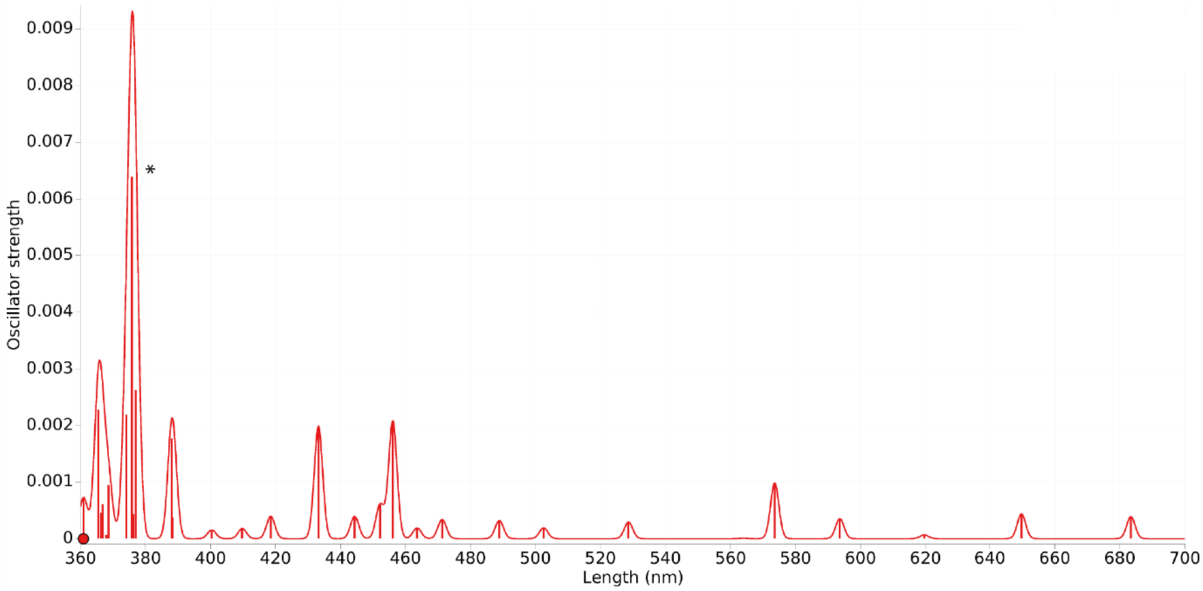

Fig S43. Calculated UV/Vis spectrum for **5** (Gaussian width 3.0 nm). Indicated with an asterisk (*) is excited state 21, which has a considerable oscillator strength

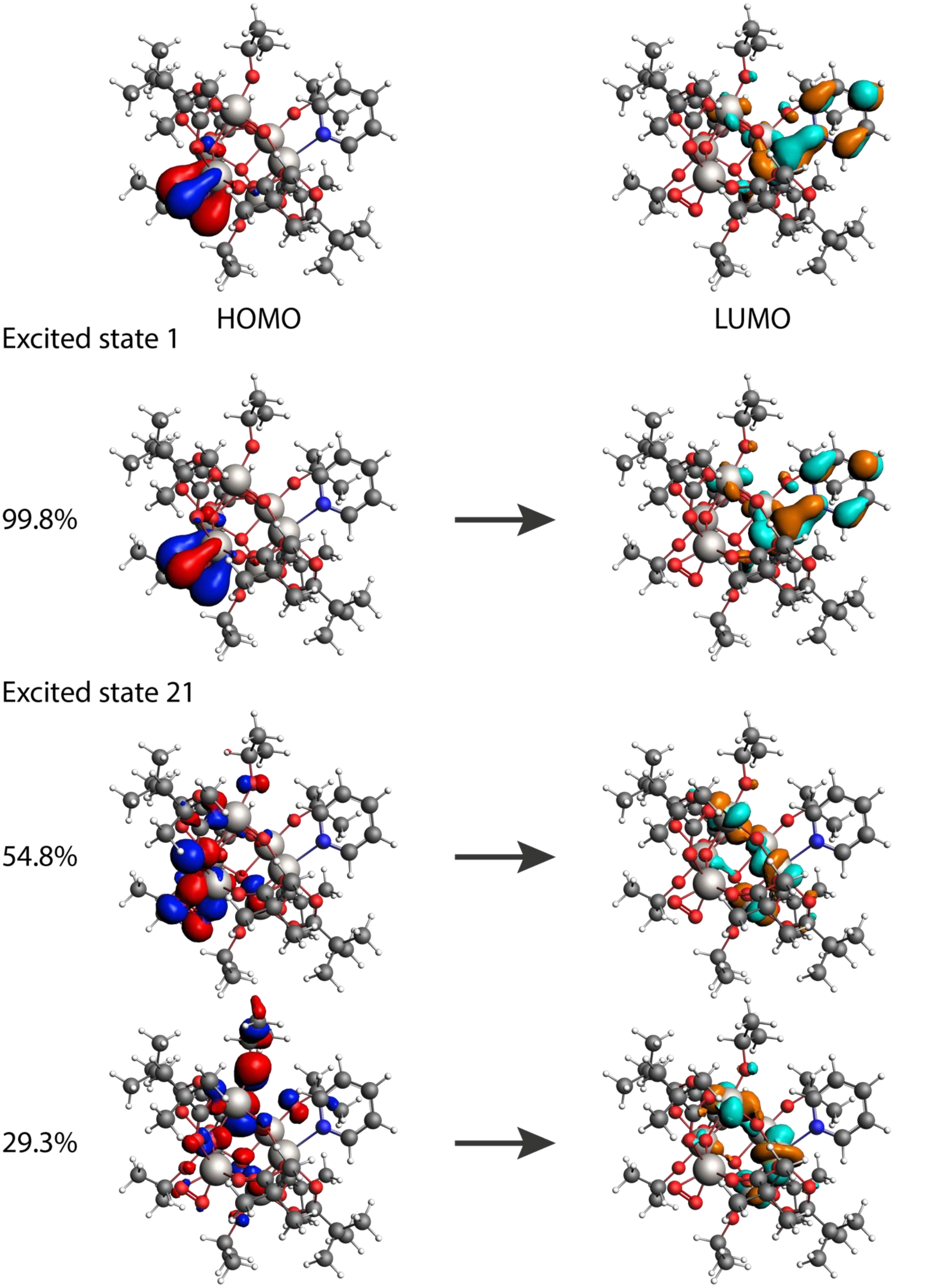

Fig S44. HOMO, LUMO and natural transition orbitals for the excited states 1 and 21 with significant oscillator strength and their percentage in the transition for **5** (red/blue donor orbital, cyan/orange acceptor orbital).

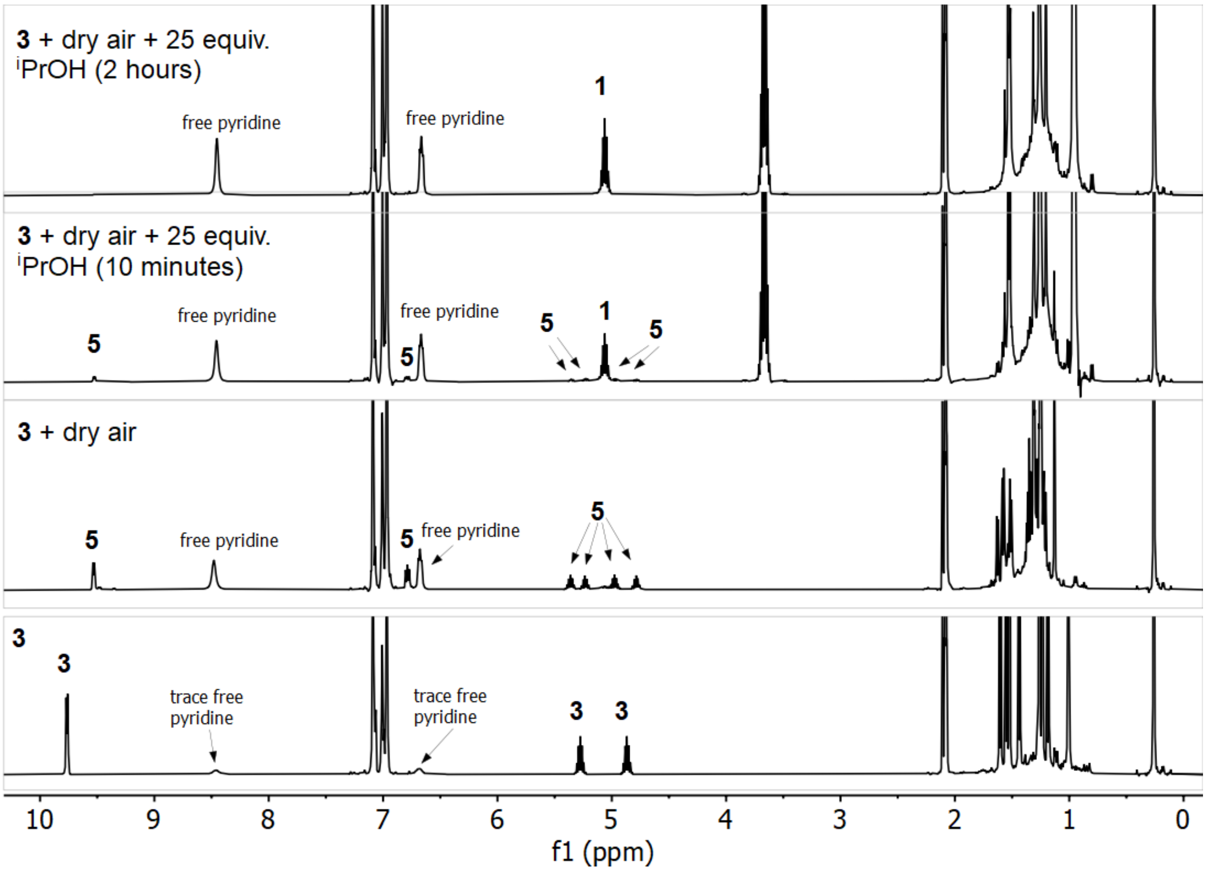

Fig S45. ^{1}H NMR spectra showing reaction of **3** with dry air and then subsequent addition of excess ^{i}PrOH.

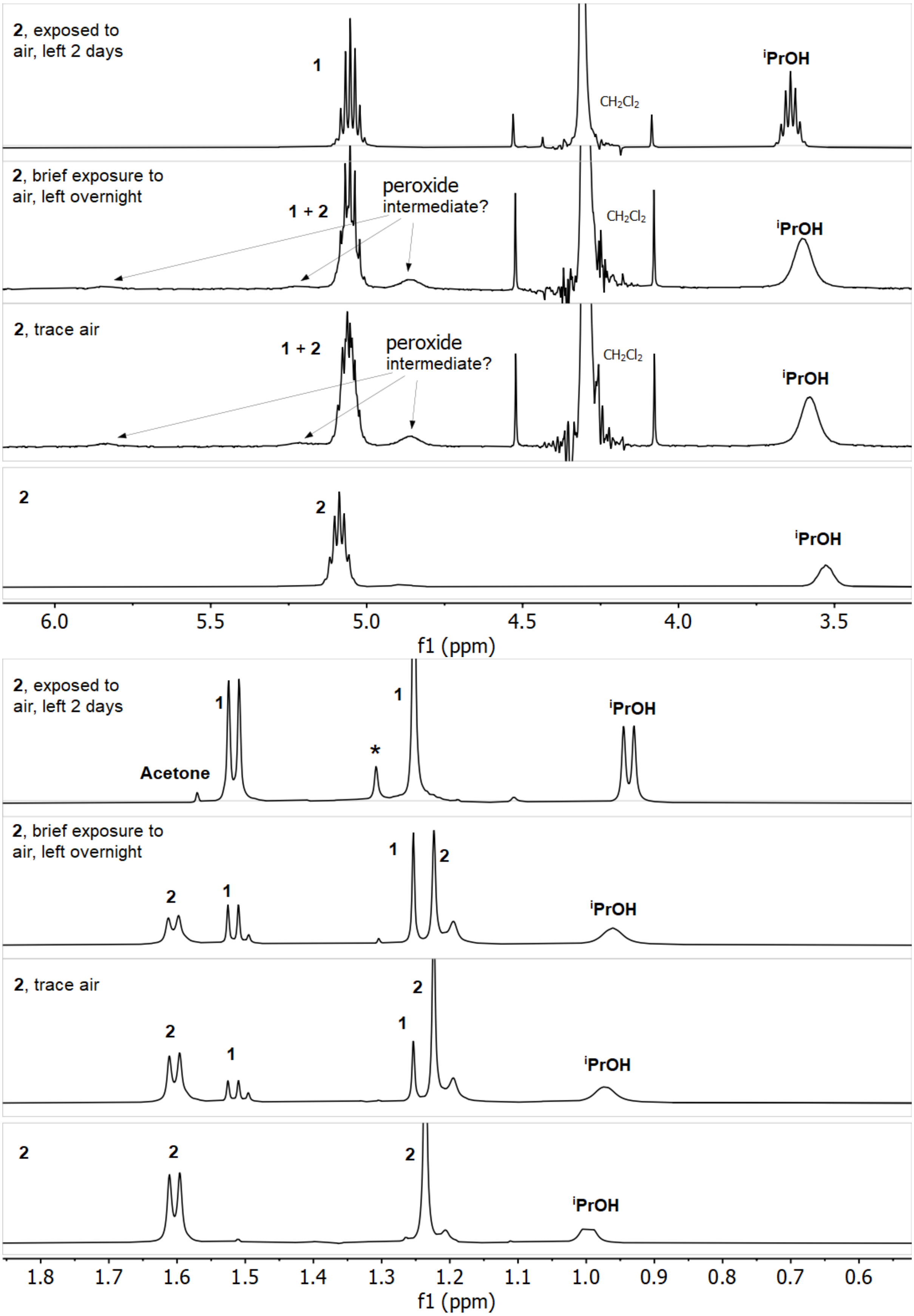

Fig S46. ^{1}H NMR spectra showing reaction of **2** with air. Top) OC**H**Me region, bottom) OCH**Me** and pivalate region.

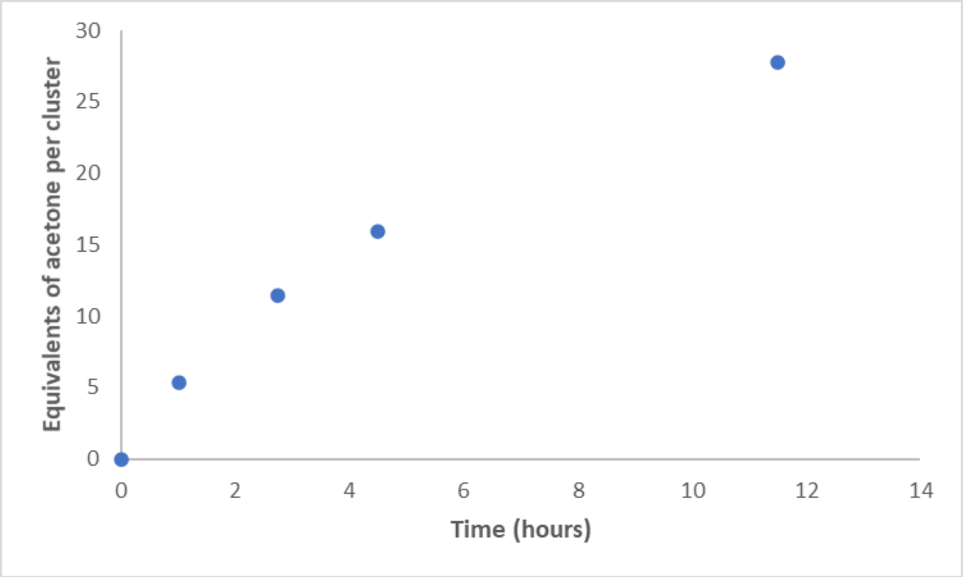

Figure S47. Equivalents of acetone per cluster identified during a photocatalytic experiment with 0.1 mol% catalyst in toluene solution.

Supporting note 2. Crystallography of 5

It proved very difficult to grow crystals of **5**, as **5** is very sensitive when in the solution phase. Note that any trace moisture can catalyse a chain reaction in which any hydrolysed isopropanol can be oxidised by **5**, generating further water to continue the decomposition process. This makes solutions of **5** prone to decomposition especially over the timescale required for crystallisation. Crystals of **5** could be grown by dissolving powdered **5** in the minimal quantity of dry dichloromethane within a glovebox and storing the solution at –30 °C in the glovebox freezer. The crystal structure of **5** solved with a unit cell similar to related structure **3**. The peroxide O atoms show slightly larger displacement ellipsoids to the rest of the structure indicating some minor disorder. It is perhaps possible that due to the highly sensitive nature of **5** and the similar shape and size to other clusters (e.g. **1**, **3**) that some minor co-crystallisation of similar clusters occurs such that the peroxide fragment is not resolved perfectly (i.e.. may not be 100% **5** in the crystal). This may account for the slight differences in bond-metrics compare to calculated structure of **5**.

Supporting note 3. Testing for hydrogen peroxide

Compound **5** reacts with ^{i}PrOH to give **1**, acetone, water and pyridine. The reaction mixture (after 1 minute and 1 day) and a separate solution of **5** was tested for the presence of H_2O_2 using Dosatest® peroxide test strips 25, and no H_2O_2 was detected. A control experiment confirmed the test strips clearly detected H_2O_2 at equivalent concentrations.

Supporting note 4. Photocatalytic experiments

Photocatalytic experiments were conducted with either 5 or 0.1 mol% catalyst, with stirring under a 302 nm light source. Different glassware were tested (open or closed flasks, glass or quartz) to identify best conditions. It is probable that the UV light irradiance was crucial for reactivity, which will vary

between shape of flask. It was challenging to allow access to air whilst stopping loss of volatile reagents and products, hence results are approximate and will give an underestimate of product formation.

Control experiments were conducted with no catalyst, or with TiO_2 powder (anatase 325 mesh (<44 µm) powder) at an equivalent Ti content to 5 or 0.1 mol% **1**. With no catalyst negligible acetone was observed, with TiO_2 a small amount of acetone was observed, at both concentrations the amount of acetone produced was 0.15 times that produced using compound **1** under the same conditions.

Photocatalyst	Reagent	Catalyst loading	Time under UV lamp	Equivalents of ketone/adehyde per cluster
**1** (closed flask, Schlenk, *thick glass*)	isopropanol	5 mol%	6	2.4
**1** (open flask, test tube, *glass*)	isopropanol	5 mol%	4	4.6
**1** (open flask, cuvette, *quartz*)	isopropanol	5 mol%	4	7.3
**1** (open flask. test tube, *glass*)	isopropanol	0.1 mol%	4.5 (11.5)	15.9 (27.8)
**1** (open flask. test tube, *glass*)	n-butanol	0.1 mol%	4.5	16
**1** (open flask. test tube, *glass*)	n-octanol	0.2 mol%	4.5	13

Table S6. Photocatalytic experimental data

Using **1** as catalyst, the reaction solution appears pale yellow during irradiation indicating that oxidation by air is occurring faster than generation of the dark-blue-coloured mixed valent intermediates (e.g. **2**). Considering that the photoactivation is considered to be the slowest step, this reaction should be accelerated by a brighter light source. The best turnovers were achieved with a high concentration of isopropanol (lower catalyst loading), and, as expected, quartz flasks allow a greater proportion of high energy photons to reach the solution. Addition of pyridine to the reaction solution resulted in a slower rate or acetone production and was not beneficial.

Table S7. Crystallographic data.

Compound	**$Ti_2O^iPr_6(O_2CtBu)_2(HO^iPr)$**	**1** ***(triclinic)***	**1·(toluene)** ***(trigonal)***
Previously reported CCDC reference	*GOFCAF*	*EBEHAV (N.B. same structure but with a different space group, R–3)*	
CCDC No.	2183082	2183083	2183078
X-ray source	Cu Kα	Cu Kα	Cu Kα
Formula	$Ti_2\,O_{11}\,C_{31}\,H_{68}$	$Ti_6\,O_{24}\,C_{48}\,H_{96}$	$Ti_6\,O_{24}\,C_{55}\,H_{104}$
M	712.67	1344.67	1436.82
Crystal System	Orthorhombic	Triclinic	Trigonal
Space group	P n m a	P -1	R -3
T [K]	100	100	150
a [Å]	21.4239(2)	11.4703(2)	15.49600(10)
b [Å]	15.2022(2)	12.9048(2)	15.49600(10)
c [Å]	25.1197(3)	22.7941(4)	53.7557(7)
α [deg]	90	84.7647(14)	90
β [deg]	90	87.1313(13)	90
γ [deg]	90	76.8670(15)	120
V [Å^3]	8181.25(16)	3270.58(10)	11178.78(19)
Z	8	2	6

θ range [deg]	3.398 – 79.967	3.529 – 77.430	3.394 – 79.786
Reflns collected	34781	13254	49859
R int	0.037	0.085	0.208
No. of data/restr/param	8782/114/100	13198/432/815	4571/444/346
R_1 [I>2σ(I)]	0.0789	0.0556	0.1217
wR_2 [all data]	0.2302	0.1559	0.3993
GoF	0.987	0.9973	1.9014
Largest diff. pk and hole [eÅ^3]	-0.84 – 1.67	-0.82 – 1.13	-1.46 – 2.98

Compound	**2**·(toluene)	**3**	**5**·$(CH_2Cl_2)_{0.57}$
CCDC No.	2183079	2183080	2183081
X-ray source	Cu Kα	Cu Kα	Cu Kα
Formula	$Ti_6\ O_{26}\ C_{61}\ H_{122}$	$Ti_6\ O_{22}\ C_{59}\ N_2\ H_{100}$	$Ti_6\ O_{24}\ C_{47.57}\ N_1\ H_{88.14}\ Cl_{1.14}$
M	1559.02	1476.84	1436.82
Crystal System	Triclinic	Monoclinic	Monoclinic
Space group	P -1	P 21/c	P 21/n
T [K]	100	100	100
a [Å]	13.2495(3)	14.56680(10)	14.4104(3)
b [Å]	13.4140(4)	24.34990(10)	20.6054(7)
c [Å]	14.7584(4)	21.77240(10)	24.3253(6)
α [deg]	63.032(3)	90	90
β [deg]	64.038(2)	106.6604(5)	97.146(2)
γ [deg]	65.591(3)	90	90
V [Å^3]	2024.99(11)	7398.48(7)	7166.9(3)
Z	2	4	4
θ range [deg]	3.532 – 79.937	2.789 – 80.033	3.391 – 77.573
Reflns collected	29287	184417	74891
R int	0.077	0.064	0.102
No. of data/restr/param	8575/228/442	15965/0/802	14537/672/871
R_1 [I>2σ(I)]	0.0631	0.0627	0.1089
wR_2 [all data]	0.1734	0.1690	0.2815
GoF	1.0070	1.0227	1.0047
Largest diff. pk and hole [eÅ^3]	-1.21 – 0.86	-0.69 – 2.35	-0.94 – 1.18

Computational data

Table S8. Cartesian coordinates of optimized **1** (PBE/TZ2P) in Å (n_{imag} = 0).

Atom	X	Y	Z
C	10.534079	2.895851	13.649396
C	8.996878	0.942226	13.104832
C	12.137441	-0.161352	19.318179
C	12.165840	-0.391934	16.808785
C	13.565470	1.379614	17.929331

H	8.486877	3.034105	12.983384
H	10.567041	3.909028	14.068953
H	11.164137	2.241973	14.267958
H	10.944090	2.917520	12.629495
H	9.381114	0.884623	12.076525
C	1.958991	7.563395	17.902967
H	7.033144	5.904158	22.836271
H	4.952546	5.034079	21.747609
H	7.953451	0.601373	13.107072
H	9.585677	0.265789	13.739711
H	12.971524	-0.872610	19.404758
H	11.198393	-0.729498	19.381717
H	12.179744	0.534982	20.164997
H	12.998913	-1.106382	16.876721
H	12.235077	0.125365	15.842655
H	11.223762	-0.954424	16.819590
H	14.411255	0.681728	18.010109
H	4.358072	6.702354	21.550911
H	4.466065	2.326032	22.672782
H	4.575852	6.023775	23.188409
H	5.542084	-2.854027	18.934024
H	6.141196	8.053460	23.745893
H	7.570286	8.336223	22.717212
H	5.939217	8.675369	22.083549
H	13.668210	1.927572	16.981443
H	13.623493	2.101274	18.753332
O	8.331548	2.854096	18.862215
O	11.568141	5.085408	18.803066
O	11.113797	6.360373	17.008945
O	9.907341	4.121458	20.897141
Ti	9.745615	4.075487	19.118112
C	11.899589	5.912268	17.904068
C	13.352496	6.426718	17.865130
C	10.246199	4.575123	22.193510
C	10.498948	6.081438	22.176128
C	11.445066	3.780206	22.708915
C	13.953640	6.036275	16.499547
C	14.184896	5.819213	18.999509
C	13.319210	7.962219	17.993420
H	9.373951	4.365567	22.842421
H	9.631853	6.610325	21.761580
H	11.375759	6.309626	21.554544
H	10.686479	6.445743	23.196361

H	11.689329	4.083361	23.736920
H	11.228220	2.704126	22.704533
H	12.320578	3.961554	22.070240
H	14.982327	6.417722	16.426250
H	13.987026	4.944049	16.378818
H	13.362488	6.457824	15.677122
H	15.216321	6.196059	18.942700
H	13.775374	6.084407	19.983127
H	14.211331	4.724076	18.933350
H	14.341316	8.361950	17.926328
H	12.898039	8.270532	18.961387
H	12.713227	8.407752	17.194866
O	7.191660	6.087701	16.958247
O	3.954891	3.857125	17.017809
O	4.409370	2.582058	18.811838
O	5.615807	4.820272	14.923219
Ti	5.777451	4.866517	16.702230
C	3.623323	3.030745	17.917224
C	2.170284	2.516766	17.956774
C	5.276535	4.366392	13.627021
C	5.023639	2.860096	13.644720
C	4.077594	5.161357	13.111873
C	1.568083	2.913140	19.320293
C	1.339179	3.119996	16.819127
C	2.202878	0.980870	17.834904
H	6.148615	4.575796	12.977844
H	5.890731	2.331176	14.059222
H	4.146906	2.632121	14.266493
H	4.835890	2.495617	12.624586
H	3.833037	4.858110	12.083963
H	4.294500	6.237424	13.116095
H	3.202249	4.980103	13.750802
H	0.539358	2.531945	19.394471
H	1.534549	4.005860	19.436325
H	2.158603	2.495043	20.144943
H	0.307475	2.744071	16.876737
H	1.749297	2.850227	15.836975
H	1.313521	4.215401	16.880553
H	1.180546	0.581905	17.903121
H	2.624451	0.668303	16.868507
H	2.808194	0.538387	18.635649
O	9.449064	4.158470	16.961131
O	9.134814	8.077078	17.026321

O	7.802201	8.316336	18.820100
O	9.147912	6.159219	14.926615
Ti	9.100224	5.994402	16.704729
C	8.586560	8.774654	17.928966
C	8.884034	10.286618	17.984644
C	8.938748	6.679533	13.627839
C	7.749929	7.638500	13.629598
C	10.227485	7.335536	13.134104
C	9.600065	10.567711	19.322138
C	9.772075	10.718988	16.812764
C	7.545288	11.047896	17.947424
H	8.703383	5.817913	12.973606
H	6.858044	7.141993	14.031508
H	7.972326	8.514035	14.255073
H	7.538496	7.981473	12.606663
H	10.099454	7.699681	12.104851
H	11.058599	6.618625	13.149951
H	10.488667	8.185792	13.779204
H	9.803667	11.644489	19.412611
H	10.559869	10.034572	19.377788
H	8.979391	10.254759	20.171082
H	9.978341	11.796650	16.884576
H	9.283708	10.526388	15.848262
H	10.728499	10.181158	16.816075
H	7.730869	12.128178	18.034006
H	7.014097	10.870554	17.001047
H	6.894376	10.734278	18.772676
H	2.543610	9.806430	16.410146
H	4.316834	9.663692	16.423192
H	6.211340	-1.572392	19.973260
H	3.331057	8.394199	15.653955
H	4.782699	-1.242005	18.976828
H	7.814204	-3.181108	17.830964
H	8.503923	-1.909692	18.865477
H	8.655133	-1.788550	17.095080
H	2.532061	10.056404	18.936434
H	3.301205	8.830747	19.973839
O	8.875198	5.776019	18.862721
H	4.307445	9.903991	18.984566
O	5.327089	7.462923	18.794483
O	4.453255	6.428498	17.001024
O	6.986057	6.503957	20.895472
Ti	7.110011	6.389513	19.116679

H	1.112691	8.260723	17.822860
H	1.861939	7.021338	18.854832
C	4.447970	7.336331	17.892561
H	1.896210	6.836730	17.083699
C	3.284333	8.346658	17.841509
C	6.422896	6.566060	22.191817
C	4.986687	6.046503	22.168862
C	6.526340	7.996953	22.717863
C	6.648526	-1.346487	17.842559
Ti	6.422884	2.947929	19.115868
C	5.296994	1.608900	22.688221
O	6.647831	3.166067	16.957942
O	10.197085	1.479773	17.024707
O	11.070131	2.513863	18.818771
C	5.958911	-1.642778	16.494896
H	7.986341	0.963102	23.214454
H	5.034990	0.758433	22.043723
C	6.938850	0.167091	17.893616
C	6.585486	2.264283	22.193070
C	7.774155	1.305105	22.191355
O	6.073869	4.783759	18.859383
O	8.536479	2.437580	14.925130
C	3.378301	9.095817	16.496105
Ti	8.413080	2.552605	16.703876
C	3.365188	9.341993	19.004164
C	11.075740	1.606135	17.927088
C	12.239780	0.596242	17.978004
C	9.098617	2.374149	13.628420
H	5.763642	-2.721498	16.409321
H	6.592583	-1.332415	15.654678
H	4.996897	-1.116310	16.417512
H	5.425811	1.245176	23.717525
C	7.991116	-2.098847	17.911228
C	5.741603	-1.774865	19.001674
H	8.665775	1.801133	21.788275
H	6.821463	3.126396	22.846426
H	7.551162	0.428985	21.566908
O	6.388774	0.864814	18.794971
O	7.722090	0.625789	17.001650
O	6.375530	2.783566	20.894012

Table S9. Cartesian coordinates of optimized closed-shell **2** (PBE/TZ2P) in Å

Atom	X	Y	Z
C	6.758367	0.742314	2.205553
C	6.073598	1.973998	1.616481
C	5.797472	-0.433277	2.373251
C	14.277945	4.192414	4.873196
C	14.648007	4.636875	6.279312
C	15.446474	3.622531	4.083051
C	10.487354	-1.261657	3.543580
C	10.763097	-2.419493	2.565821
C	9.419711	-3.066806	2.177663
C	11.425388	-1.814385	1.310581
C	11.689985	-3.463890	3.198305
C	10.293131	6.321180	4.752683
C	10.533615	7.803346	4.408443
C	11.037869	7.913592	2.958586
C	11.624078	8.313686	5.375646
C	9.253017	8.626776	4.587935
C	10.509350	2.898877	1.891584
C	10.803987	3.178756	0.403992
C	12.266022	2.776278	0.125699
C	9.859307	2.385572	-0.505561
C	10.629501	4.690499	0.158255
O	9.084928	3.216734	4.697070
O	10.855183	3.831693	6.648125
O	11.065958	1.537677	4.823779
O	6.865997	4.274968	6.055467
O	7.341954	1.064192	3.461866
O	13.194668	3.216045	4.905341
O	9.660930	-0.370911	3.164836
O	11.092539	-1.246593	4.656992
O	9.195732	5.990663	5.298710
O	11.232060	5.500060	4.487467
O	11.251528	3.501121	2.742375
O	9.573053	2.112499	2.200135
Ti	9.006059	1.334810	4.150663
Ti	11.008608	3.431603	4.800844
Ti	8.690269	4.223986	6.352766
C	6.058380	4.645882	4.941573
C	4.820595	3.752865	4.907328
C	5.694040	6.126516	5.049625

H	7.578389	0.439597	1.529317
H	6.789709	2.800732	1.529777
H	5.239423	2.296396	2.255037
H	5.676973	1.747681	0.616168
H	6.318449	-1.296901	2.806768
H	4.963074	-0.160697	3.035069
H	5.383283	-0.730716	1.399018
H	13.831767	5.034309	4.325801
H	13.756620	4.956116	6.832455
H	15.357377	5.475404	6.233895
H	15.126062	3.818992	6.837530
H	15.126288	3.315390	3.079375
H	16.239590	4.376228	3.981944
H	15.877275	2.750105	4.597908
H	9.594269	-3.872849	1.450598
H	8.742873	-2.329546	1.728731
H	8.921441	-3.504892	3.054647
H	11.610502	-2.607301	0.571650
H	10.777800	-1.054119	0.855624
H	12.389438	-1.347451	1.557682
H	11.880277	-4.272323	2.477735
H	11.241242	-3.903104	4.099044
H	12.651197	-3.021908	3.488888
H	11.259531	8.964371	2.722560
H	11.948382	7.320182	2.811675
H	10.279451	7.558708	2.246470
H	11.842693	9.369493	5.160147
H	12.550495	7.735577	5.264448
H	11.292987	8.240896	6.421368
H	9.458972	9.682885	4.361518
H	8.872954	8.557174	5.614736
H	8.458706	8.281023	3.912985
H	12.517592	2.995340	-0.922168
H	12.954254	3.330940	0.775678
H	12.420586	1.701028	0.294862
H	10.095222	2.597811	-1.558465
H	9.959126	1.305064	-0.339720
H	8.810486	2.654914	-0.326026
H	10.872955	4.927172	-0.887730
H	11.288754	5.272272	0.814104
H	9.592097	5.004660	0.343537
H	6.640468	4.482660	4.014627
H	4.171859	4.016159	4.059535

H	4.240492	3.876583	5.834504
H	5.113959	2.700552	4.799930
H	5.093243	6.438784	4.182985
H	6.601164	6.741707	5.090425
H	5.109157	6.307105	5.963216
H	13.478014	2.361437	5.289632
C	12.956772	3.673421	10.306755
C	13.642292	2.441550	10.894841
C	13.917750	4.848787	10.137899
C	5.433729	0.238687	7.637017
C	5.070675	-0.215407	6.231997
C	4.261474	0.816170	8.416312
C	9.226887	5.678875	8.969520
C	8.945315	6.833341	9.949770
C	10.285825	7.482917	10.343535
C	8.281259	6.222597	11.201383
C	8.017335	7.877045	9.317987
C	9.412334	-1.904522	7.756440
C	9.167445	-3.386939	8.095672
C	8.663848	-3.500364	9.545687
C	8.074992	-3.891321	7.127424
C	10.445917	-4.213180	7.912841
C	9.204609	1.517559	10.620912
C	8.908942	1.239182	12.108503
C	7.447094	1.643492	12.385590
C	9.853973	2.031954	13.018077
C	9.081457	-0.272531	12.355646
O	10.629440	1.199936	7.815663
O	8.859381	0.586139	5.864596
O	8.651030	2.880047	7.692714
O	12.846016	0.130862	6.464667
O	12.371763	3.352085	9.051242
O	6.519729	1.212044	7.606750
O	10.054939	4.789669	9.347843
O	8.621946	5.663320	7.855877
O	10.511384	-1.575082	7.213089
O	8.475107	-1.081667	8.022798
O	8.464223	0.913018	9.770119
O	10.140029	2.305003	10.312350
Ti	10.707604	3.082744	8.362131
Ti	8.705515	0.986079	7.712185
Ti	11.024474	0.191374	6.161653
C	13.654381	-0.231950	7.580307

C	14.890171	0.664112	7.609870
C	14.021626	-1.712376	7.479836
H	12.137901	3.976177	10.984403
H	12.925941	1.615135	10.983165
H	14.475064	2.118566	10.254681
H	14.040874	2.667815	11.894378
H	13.396395	5.712680	9.705383
H	14.751162	4.576094	9.474852
H	14.333363	5.145813	11.111658
H	5.874315	-0.600674	8.192830
H	5.964360	-0.540456	5.685937
H	4.359130	-1.052045	6.279146
H	4.597816	0.599619	5.665387
H	4.575972	1.129361	9.419908
H	3.466346	0.064646	8.517361
H	3.835682	1.686028	7.893058
H	10.107212	8.286363	11.072511
H	10.963325	6.746139	10.792218
H	10.785411	7.924854	9.469246
H	8.091820	7.012687	11.942241
H	8.929806	5.462853	11.655871
H	7.319250	5.753625	10.950247
H	7.822979	8.682820	10.040483
H	8.467280	8.320233	8.419797
H	7.058054	7.433477	9.023524
H	8.439950	-4.551329	9.778653
H	7.754919	-2.905144	9.695057
H	9.423725	-3.149344	10.258212
H	7.853453	-4.947077	7.340049
H	7.150433	-3.310619	7.240661
H	8.405938	-3.816511	6.081774
H	10.237194	-5.269653	8.135009
H	10.826291	-4.140382	6.886356
H	11.241052	-3.872247	8.589301
H	7.194758	1.425453	13.433474
H	6.758789	1.088906	11.735656
H	7.293756	2.718760	12.215469
H	9.617202	1.820600	14.070983
H	9.755516	3.112443	12.851458
H	10.902584	1.761208	12.839239
H	8.836965	-0.508158	13.401636
H	8.422047	-0.854205	11.699801
H	10.118626	-0.588062	12.171415

H	13.071586	-0.064759	8.505995
H	15.539629	0.406439	8.458845
H	15.470185	0.537480	6.683109
H	14.594476	1.716329	7.712762
H	14.621688	-2.019319	8.348894
H	13.115815	-2.329492	7.440262
H	14.608364	-1.896056	6.568068
H	6.241727	2.064482	7.212878

Table S10. Cartesian coordinates (Å) and hyperfine couplings (MHz) of optimized triplet **2** (PBE/TZ2P).

Atom	X	y	Z	A
C	6.822125	0.797946	2.206230	0.528
C	6.244761	1.942921	1.376061	0.322
C	5.758798	-0.214138	2.628958	0.044
C	14.423403	4.022767	4.677172	2.293
C	15.316979	4.524987	5.801804	0.120
C	15.148300	3.152675	3.662878	-0.012
C	10.489333	-1.215297	3.515094	-0.140
C	10.718915	-2.393210	2.545861	0.507
C	9.381364	-3.145242	2.391617	-0.028
C	11.144070	-1.815158	1.182114	-0.029
C	11.798263	-3.343719	3.075835	-0.030
C	10.261702	6.354289	4.862727	2.620
C	10.452125	7.838054	4.485868	-0.114
C	10.908234	7.927445	3.018178	0.003
C	11.557927	8.400696	5.404305	0.005
C	9.156198	8.632143	4.684887	0.005
C	10.808207	2.966284	1.986026	0.370
C	11.127000	3.289046	0.511567	0.119
C	12.650410	3.310634	0.302342	-0.024
C	10.479770	2.265374	-0.428858	-0.001
C	10.550233	4.694943	0.234372	0.010
O	9.160827	3.308696	4.735164	2.996
O	10.769489	3.850787	6.747187	2.089
O	11.115747	1.554414	4.811007	3.400
O	6.807071	4.192770	5.992502	-0.472
O	7.465330	1.318997	3.358230	-0.766
O	13.287387	3.293364	5.236406	-0.659
O	9.637000	-0.341083	3.168641	-0.892
O	11.149882	-1.189392	4.599526	0.043
O	9.162412	5.998982	5.389229	0.115

O	11.241041	5.570625	4.639809	-0.700
O	11.495044	3.569157	2.877188	0.317
O	9.863426	2.164909	2.238942	-1.097
Ti	9.109239	1.470796	4.115364	2.783
Ti	11.137698	3.482887	4.937809	10.397
Ti	8.606319	4.207274	6.379138	4.555
C	6.007236	4.545013	4.863754	2.118
C	4.794722	3.619949	4.804251	-0.041
C	5.609661	6.016627	4.963037	-0.261
H	7.591136	0.275531	1.607506	-0.175
H	7.036288	2.654457	1.108540	0.002
H	5.469823	2.478225	1.942841	0.004
H	5.793762	1.556612	0.450592	0.033
H	6.210342	-1.013154	3.231304	0.005
H	4.977067	0.275307	3.227642	0.003
H	5.285865	-0.666968	1.745585	0.071
H	13.950467	4.874998	4.169014	-0.132
H	14.734309	5.104843	6.528128	0.050
H	16.109119	5.168881	5.394322	0.233
H	15.796054	3.686264	6.327337	-0.001
H	14.459709	2.816524	2.878360	0.051
H	15.965196	3.717689	3.193157	0.106
H	15.589834	2.270213	4.151301	-0.001
H	9.502980	-3.974509	1.679868	0.013
H	8.595518	-2.475936	2.019709	-0.003
H	9.050552	-3.568418	3.351100	0.014
H	11.271128	-2.631522	0.456511	-0.013
H	10.388208	-1.117815	0.800345	0.001
H	12.100912	-1.279295	1.260705	0.012
H	11.941481	-4.171795	2.366554	0.003
H	11.517280	-3.765350	4.049444	0.004
H	12.757996	-2.826722	3.203697	-0.007
H	11.102075	8.976771	2.752607	-0.008
H	11.825373	7.347872	2.856508	-0.005
H	10.133763	7.543832	2.338737	0.004
H	11.742278	9.456206	5.157139	-0.015
H	12.494054	7.842154	5.278065	0.017
H	11.261748	8.345696	6.461610	0.004
H	9.327842	9.688715	4.432844	-0.036
H	8.806384	8.573415	5.723179	-0.001
H	8.352690	8.252669	4.039692	0.005
H	12.877360	3.578277	-0.739705	-0.004
H	13.128339	4.043851	0.963116	-0.003

H	13.092936	2.323778	0.501037	0.004
H	10.705911	2.528238	-1.472427	-0.040
H	10.860947	1.253059	-0.238806	-0.001
H	9.390398	2.240666	-0.304427	0.003
H	10.763230	4.983605	-0.805217	0.027
H	10.998260	5.441134	0.903112	0.002
H	9.460251	4.710491	0.375654	0.001
H	6.614780	4.391278	3.952843	0.190
H	4.163808	3.862636	3.937065	-0.083
H	4.184214	3.735110	5.712908	-0.005
H	5.117538	2.574681	4.710830	0.008
H	5.022138	6.315495	4.082786	-0.043
H	6.502599	6.650961	5.022896	0.035
H	5.000937	6.186584	5.862977	0.014
H	13.528606	2.369958	5.461363	1.210
C	12.889401	3.627027	10.302150	0.529
C	13.462863	2.485189	11.139371	0.326
C	13.956343	4.632478	9.872614	0.040
C	5.287898	0.386365	7.832164	2.287
C	4.394143	-0.107117	6.703858	0.119
C	4.562739	1.245945	8.855250	-0.011
C	9.219631	5.634922	8.997681	-0.142
C	8.989334	6.812741	9.966790	0.508
C	10.327033	7.563787	10.123918	-0.028
C	8.561108	6.234344	11.329523	-0.029
C	7.911571	7.764205	9.435229	-0.030
C	9.447393	-1.935633	7.648605	2.618
C	9.256164	-3.419854	8.023465	-0.114
C	8.804105	-3.510568	9.492317	0.004
C	8.147217	-3.979984	7.107326	0.005
C	10.550662	-4.215090	7.819865	0.005
C	8.900937	1.452961	10.527207	0.362
C	8.586766	1.126991	12.002002	0.120
C	7.064713	1.060829	12.209988	-0.024
C	9.202814	2.172418	12.939786	0.000
C	9.204970	-0.260315	12.284371	0.009
O	10.548405	1.110612	7.777863	2.998
O	8.939322	0.568620	5.765752	2.091
O	8.592863	2.864680	7.701910	3.389
O	12.902256	0.227645	6.520157	-0.473
O	12.243565	3.101111	9.153961	-0.771
O	6.422101	1.122889	7.278766	-0.642
O	10.072088	4.760856	9.344356	-0.891

O	8.559252	5.608801	7.913167	0.043
O	10.546884	-1.580125	7.122600	0.115
O	8.468269	-1.151929	7.872129	-0.700
O	8.215422	0.848651	9.636096	0.318
O	9.844706	2.255579	10.274234	-1.100
Ti	10.599329	2.948664	8.397794	2.784
Ti	8.571871	0.936133	7.575429	10.388
Ti	11.102734	0.211941	6.133600	4.571
C	13.700412	-0.123845	7.650484	2.131
C	14.913703	0.800120	7.710577	-0.041
C	14.096964	-1.595912	7.553540	-0.262
H	12.122663	4.156162	10.897914	-0.172
H	12.668667	1.778719	11.412462	0.002
H	14.234951	1.942889	10.575345	0.004
H	13.916437	2.875756	12.061776	0.033
H	13.507547	5.429540	9.265685	0.005
H	14.735782	4.136376	9.276464	0.003
H	14.431531	5.088750	10.752987	0.071
H	5.763015	-0.469519	8.332028	-0.132
H	4.977198	-0.679365	5.971815	0.050
H	3.603380	-0.755950	7.106116	0.232
H	3.913224	0.735544	6.186325	-0.001
H	5.251967	1.575591	9.641987	0.051
H	3.747341	0.675311	9.320785	0.106
H	4.119214	2.132253	8.375682	-0.001
H	10.204508	8.392687	10.835952	0.012
H	11.111702	6.893791	10.496953	-0.003
H	10.659880	7.987277	9.165313	0.014
H	8.432592	7.050502	12.055101	-0.013
H	9.315826	5.536524	11.712763	0.001
H	7.604276	5.698751	11.248613	0.012
H	7.767411	8.591981	10.144666	0.003
H	8.194726	8.186176	8.462385	0.004
H	6.951786	7.247846	9.305189	-0.007
H	8.609398	-4.559961	9.757014	-0.008
H	7.888422	-2.929725	9.657603	-0.005
H	9.581316	-3.129284	10.169982	0.004
H	7.962137	-5.035573	7.353621	-0.015
H	7.212108	-3.420434	7.236721	0.017
H	8.440596	-3.924037	6.049281	0.004
H	10.378435	-5.271877	8.070608	-0.036
H	10.897950	-4.155044	6.780805	-0.001
H	11.356228	-3.837565	8.463612	0.005

H	6.844665	0.789503	13.252573	-0.004
H	6.608828	0.312398	11.550692	-0.004
H	6.593360	2.033661	12.008342	0.004
H	8.983457	1.906635	13.984058	-0.039
H	8.791909	3.172482	12.746153	-0.001
H	10.291006	2.228875	12.815902	0.003
H	8.999548	-0.551530	13.324768	0.025
H	8.779524	-1.021909	11.618160	0.002
H	10.294959	-0.243882	12.143804	0.001
H	13.091775	0.031540	8.560378	0.185
H	15.542787	0.558164	8.579284	-0.083
H	15.525751	0.682987	6.803221	-0.005
H	14.591842	1.845857	7.801881	0.008
H	14.682983	-1.894111	8.435017	-0.043
H	13.203663	-2.229671	7.493142	0.035
H	14.706876	-1.767419	6.654699	0.015
H	6.177254	2.045652	7.055618	1.213

Table S11. Cartesian coordinates of optimized closed-shell **3** (PBE/TZ2P) in Å

Atom	X	Y	Z
Ti	0.532099	18.933805	8.141291
Ti	3.571213	17.070378	7.469001
Ti	0.682863	15.439784	8.689699
Ti	-1.155926	16.946656	6.674018
Ti	1.845736	18.790683	5.781506
Ti	1.779869	15.220321	5.746297
O	2.374200	18.532925	7.753347
O	2.452447	16.975940	5.559907
O	2.350716	15.636227	7.783511
O	0.065336	15.529150	6.575856
O	-0.095770	17.165992	8.563659
O	-0.000321	18.440173	6.266740
O	-2.532592	15.688473	7.645874
C	-2.335928	14.875626	8.598592
O	-1.213721	14.678167	9.157614
C	-3.529685	14.017258	9.068115
C	-3.497573	12.736873	8.203041
C	-4.853655	14.760784	8.842748
C	-3.377063	13.641598	10.548899
O	-2.110583	16.821571	5.136917
C	-3.367316	17.126447	4.556638
C	-4.120915	15.832189	4.253228

C	-3.165114	17.993239	3.315131
O	-2.599407	18.319472	7.395546
C	-2.476484	19.299958	8.189880
O	-1.367470	19.689663	8.673602
C	-3.760555	20.073529	8.553798
C	-4.256960	20.766668	7.267215
C	-3.490670	21.123645	9.637466
C	-4.821960	19.070641	9.042248
O	1.204942	15.431299	10.404858
C	1.202913	15.221102	11.801122
C	1.362199	13.730406	12.097829
C	-0.064161	15.811860	12.419093
O	0.914553	13.366061	8.453485
C	1.237046	12.696700	7.427405
O	1.562444	13.202666	6.308266
C	1.245369	11.156107	7.530561
C	0.313784	10.592166	6.439933
C	0.774805	10.693857	8.914305
C	2.686547	10.672491	7.275487
O	3.682180	14.459849	5.363099
C	4.803644	14.752775	5.884876
O	4.963071	15.632977	6.782117
C	6.040986	14.007565	5.344130
C	6.268677	14.512976	3.902624
C	7.278876	14.297491	6.200757
C	5.755775	12.495939	5.316674
O	1.165622	14.817488	4.077312
C	0.450706	13.773170	3.429409
C	1.444045	12.784688	2.820454
C	-0.489937	14.367238	2.384003
O	4.983936	18.253179	6.476578
C	4.832285	19.162509	5.602500
O	3.704660	19.526387	5.147297
C	6.106797	19.858196	5.086849
C	6.821054	20.494204	6.296417
C	5.768996	20.934475	4.049227
C	7.016804	18.784178	4.458737
O	4.490935	17.137255	9.045623
C	5.579425	16.495562	9.694582
C	5.053200	15.533507	10.758120
C	6.513966	17.556750	10.272883
O	0.797219	21.010866	7.928734
C	1.179248	21.553596	6.844102

O	1.553698	20.891812	5.827260
C	1.186366	23.091636	6.786269
C	2.144032	23.597776	7.884463
C	-0.243078	23.589664	7.078236
C	1.648532	23.594328	5.413981
N	1.091652	19.399189	10.283847
C	0.372434	20.278591	11.015298
C	0.708168	20.623830	12.319713
C	1.831471	20.042859	12.908531
C	2.578735	19.138735	12.154956
C	2.184672	18.845935	10.852722
N	1.352506	19.017840	3.624698
C	1.643131	20.161756	2.961701
C	1.398324	20.326580	1.604152
C	0.832122	19.276088	0.881028
C	0.537241	18.093978	1.559480
C	0.808740	18.000504	2.919959
H	-4.329116	12.076466	8.489159
H	-3.600062	12.978304	7.136192
H	-2.556561	12.188728	8.347854
H	-5.693824	14.120652	9.148596
H	-4.895899	15.685754	9.434417
H	-4.984321	15.030910	7.788215
H	-4.212809	12.995704	10.854760
H	-3.388252	14.535503	11.188511
H	-2.436873	13.106662	10.728129
H	-3.941596	17.705071	5.304493
H	-5.124570	16.055388	3.863234
H	-4.224205	15.231477	5.165656
H	-3.581246	15.236833	3.502935
H	-4.136319	18.283557	2.889155
H	-2.607358	18.904128	3.568159
H	-2.600972	17.443394	2.548267
H	-5.189078	21.311433	7.476007
H	-3.516654	21.488817	6.894182
H	-4.451972	20.030939	6.476726
H	-4.418982	21.667667	9.864107
H	-2.735615	21.850476	9.311298
H	-3.135629	20.655726	10.565815
H	-5.763746	19.598193	9.251813
H	-4.498734	18.572729	9.967804
H	-5.009114	18.298548	8.286450
H	2.080933	15.758623	12.207797

H	1.408506	13.560149	13.182966
H	2.283385	13.344060	11.643057
H	0.512046	13.169797	11.686094
H	-0.040820	15.703742	13.512966
H	-0.151520	16.878150	12.174017
H	-0.950267	15.292496	12.030462
H	0.323511	9.493172	6.477637
H	-0.722778	10.927254	6.589148
H	0.639482	10.910783	5.442061
H	0.790447	9.595198	8.961115
H	-0.247453	11.034625	9.124032
H	1.423733	11.084489	9.708451
H	2.719465	9.573626	7.305051
H	3.373916	11.053267	8.044564
H	3.045175	11.009056	6.295186
H	7.132899	13.994212	3.462713
H	6.475097	15.592332	3.888485
H	5.387483	14.319409	3.277749
H	8.149274	13.773064	5.780517
H	7.500959	15.371408	6.232325
H	7.138372	13.953125	7.234359
H	6.614348	11.965391	4.880119
H	5.593071	12.102750	6.330094
H	4.864749	12.276988	4.716027
H	-0.153016	13.249669	4.193296
H	0.910700	11.946750	2.348299
H	2.056961	13.284073	2.056236
H	2.110065	12.388986	3.596927
H	-1.102010	13.576382	1.927120
H	-1.159595	15.103050	2.847517
H	0.087682	14.857525	1.586490
H	7.756623	20.968877	5.966916
H	6.195272	21.266175	6.766690
H	7.058348	19.735553	7.052561
H	6.695080	21.411195	3.696671
H	5.121858	21.711680	4.476650
H	5.249581	20.505181	3.182594
H	7.957157	19.244576	4.122686
H	7.250535	17.997678	5.187083
H	6.536138	18.318439	3.586403
H	6.127974	15.913143	8.932169
H	5.882790	14.973079	11.212747
H	4.531887	16.084634	11.555045

H	4.346296	14.821858	10.312975
H	7.391951	17.085750	10.738137
H	5.994476	18.154500	11.036398
H	6.859484	18.234155	9.481281
H	2.145906	24.697277	7.894483
H	1.831889	23.237244	8.872399
H	3.173211	23.257488	7.701802
H	-0.257450	24.689047	7.087879
H	-0.592682	23.224071	8.051651
H	-0.947761	23.247689	6.306660
H	1.653196	24.693843	5.406003
H	2.662489	23.243787	5.180902
H	0.978729	23.248548	4.615593
H	-0.499108	20.701840	10.517781
H	0.090486	21.340547	12.860853
H	2.120545	20.293928	13.929783
H	3.472030	18.663894	12.560640
H	2.770167	18.164927	10.234680
H	2.083840	20.955622	3.561519
H	1.654108	21.272066	1.125834
H	0.629148	19.375455	-0.185804
H	0.099195	17.238948	1.045710
H	0.604116	17.079345	3.464817

Table S12. Cartesian coordinates (Å) and hyperfine couplings (MHz) of optimized triplet **3** (PBE/TZ2P).

Atom	X	Y	Z	A
Ti	0.570706	18.895519	8.265076	12.106
Ti	3.516259	17.042521	7.560837	1.527
Ti	0.581105	15.433208	8.728853	1.242
Ti	-1.191424	16.979959	6.661759	0.900
Ti	1.798787	18.710563	5.683743	7.893
Ti	1.708254	15.168691	5.803847	1.279
O	2.392524	18.593595	7.607033	3.095
O	2.408986	16.906352	5.577328	1.659
O	2.280037	15.634710	7.837302	0.289
O	0.005546	15.512881	6.612582	0.587
O	-0.183523	17.152054	8.579844	1.897
O	0.004024	18.421949	6.283808	2.491
O	-2.599430	15.738779	7.612040	-0.987
C	-2.438330	14.915419	8.563043	-0.124
O	-1.333335	14.689017	9.145008	0.015
C	-3.670959	14.094819	9.000154	0.326

C	-3.863321	12.998087	7.929435	-0.019
C	-4.912778	15.000142	9.038653	-0.017
C	-3.444922	13.445728	10.371069	-0.018
O	-2.113954	16.897779	5.108526	-0.487
C	-3.296349	17.316238	4.449712	0.145
C	-4.175736	16.102773	4.151256	0.359
C	-2.932395	18.101137	3.190752	-0.057
O	-2.568423	18.396478	7.398540	-0.235
C	-2.422194	19.356006	8.219138	1.776
O	-1.316840	19.685220	8.749720	0.673
C	-3.681842	20.175692	8.568691	-0.089
C	-4.159860	20.865847	7.274169	0.014
C	-3.374610	21.230697	9.637667	0.074
C	-4.772963	19.212019	9.071115	0.032
O	1.087090	15.389398	10.443042	-0.278
C	1.277000	15.037620	11.797543	0.125
C	1.938103	13.662873	11.890888	0.236
C	-0.058867	15.085332	12.538978	-0.016
O	0.807731	13.347957	8.499086	-0.011
C	1.123278	12.667691	7.479871	0.133
O	1.477243	13.164654	6.363671	0.087
C	1.069693	11.127608	7.573637	0.099
C	0.003518	10.633789	6.574402	-0.010
C	0.703632	10.674067	8.991293	-0.013
C	2.446970	10.564503	7.176470	0.001
O	3.636289	14.396501	5.490533	0.075
C	4.747213	14.720779	6.006615	-0.101
O	4.900917	15.658724	6.849959	-0.739
C	5.995944	13.946704	5.536797	0.252
C	6.278512	14.402913	4.088477	-0.011
C	7.206720	14.256104	6.425207	-0.007
C	5.696941	12.437976	5.551210	0.000
O	1.169689	14.746269	4.116051	-0.879
C	0.671896	13.670689	3.335412	0.442
C	1.618150	13.415990	2.163337	0.177
C	-0.755782	13.976255	2.887737	-0.024
O	4.932501	18.252350	6.544668	-0.696
C	4.795165	19.098565	5.608807	0.497
O	3.685090	19.405199	5.073265	0.463
C	6.073701	19.801365	5.104709	-0.098
C	6.654293	20.618706	6.276122	0.026
C	5.764920	20.729411	3.924267	-0.004
C	7.085946	18.721582	4.677555	0.060

O	4.384540	17.139424	9.125632	0.116
C	5.551275	16.850146	9.877345	0.167
C	5.154073	16.228458	11.213410	-0.067
C	6.378602	18.122919	10.045874	0.105
O	0.901682	20.964162	7.910381	0.212
C	1.208345	21.480286	6.794590	5.203
O	1.502161	20.800559	5.757517	0.075
C	1.237657	23.018893	6.711621	-0.330
C	2.323487	23.514517	7.689221	0.325
C	-0.137498	23.553157	7.156648	0.018
C	1.555183	23.497987	5.290829	0.030
N	1.172090	19.244522	10.285283	-2.425
C	0.375559	18.888993	11.329170	3.882
C	0.726087	19.115292	12.650600	-3.352
C	1.945721	19.735841	12.944767	5.018
C	2.769559	20.101597	11.873725	-3.443
C	2.357881	19.845484	10.575604	3.750
N	1.346765	18.892694	3.503723	-1.504
C	1.450422	20.079122	2.862257	1.315
C	1.245298	20.212360	1.494384	-1.237
C	0.921762	19.083176	0.741214	1.678
C	0.818662	17.857957	1.399379	-1.182
C	1.036773	17.799267	2.771639	1.379
H	-4.741356	12.385433	8.180768	0.009
H	-4.019557	13.440561	6.936795	-0.002
H	-2.987733	12.335311	7.880520	0.009
H	-5.799116	14.402841	9.297224	-0.001
H	-4.804235	15.791324	9.793960	0.011
H	-5.081609	15.479871	8.067379	0.000
H	-4.325066	12.846397	10.645767	0.017
H	-3.292352	14.205021	11.150405	0.001
H	-2.564962	12.791032	10.362674	-0.001
H	-3.837842	17.986375	5.144061	-0.167
H	-5.126278	16.420323	3.698873	0.077
H	-4.393617	15.555018	5.076913	-0.008
H	-3.667967	15.422293	3.452977	-0.015
H	-3.840769	18.476470	2.697923	-0.027
H	-2.292270	18.956469	3.442792	-0.010
H	-2.391614	17.458514	2.481503	-0.009
H	-5.071329	21.445883	7.479168	-0.005
H	-3.396905	21.556863	6.888041	0.003
H	-4.383703	20.126446	6.494831	-0.008
H	-4.284935	21.806270	9.860086	0.040

H	-2.595933	21.926361	9.300718	-0.006
H	-3.024001	20.766614	10.568860	0.045
H	-5.697669	19.771472	9.274124	-0.042
H	-4.465458	18.717782	10.004060	-0.002
H	-4.985265	18.435523	8.326391	-0.003
H	1.956388	15.794825	12.232116	-0.071
H	2.148565	13.411935	12.940289	0.033
H	2.882154	13.648301	11.331600	-0.006
H	1.275698	12.895805	11.467757	-0.010
H	0.088655	14.855220	13.603804	-0.011
H	-0.510508	16.082057	12.457079	-0.010
H	-0.755477	14.352508	12.110228	0.006
H	-0.052157	9.536011	6.605970	-0.006
H	-0.990802	11.031691	6.822898	0.003
H	0.251820	10.940958	5.550698	0.001
H	0.655368	9.575980	9.026420	-0.015
H	-0.269641	11.075515	9.301025	-0.002
H	1.451192	11.009021	9.722146	0.001
H	2.416136	9.465314	7.189220	0.010
H	3.225531	10.889687	7.881336	0.003
H	2.732745	10.898350	6.171793	-0.002
H	7.151949	13.862262	3.695915	0.012
H	6.496541	15.479265	4.047417	0.009
H	5.418201	14.197298	3.438562	0.000
H	8.085330	13.708745	6.054250	-0.014
H	7.438703	15.328263	6.426034	0.000
H	7.027053	13.949770	7.464742	-0.001
H	6.565025	11.884980	5.164305	0.030
H	5.494709	12.082295	6.571357	0.007
H	4.824281	12.204848	4.929673	-0.001
H	0.657551	12.773748	3.981971	0.307
H	1.276488	12.553328	1.573402	0.007
H	1.658863	14.294294	1.502312	0.003
H	2.632684	13.211491	2.528741	-0.005
H	-1.181702	13.115087	2.353023	-0.008
H	-1.387609	14.204904	3.754867	-0.005
H	-0.772810	14.844794	2.212701	-0.001
H	7.582054	21.115912	5.957873	0.018
H	5.949396	21.395169	6.606936	0.003
H	6.881097	19.971039	7.132112	-0.009
H	6.691136	21.215483	3.584743	-0.034
H	5.047397	21.510330	4.207319	-0.005
H	5.337023	20.173199	3.080068	0.010

H	8.026021	19.196629	4.361636	-0.040
H	7.298220	18.035143	5.506498	-0.001
H	6.702531	18.133836	3.831050	-0.005
H	6.137939	16.115167	9.294811	0.005
H	6.050092	15.957607	11.789787	-0.024
H	4.559707	16.940801	11.803260	0.004
H	4.554594	15.324110	11.052387	0.003
H	7.315641	17.902567	10.576823	-0.036
H	5.816409	18.869244	10.625363	-0.002
H	6.622364	18.551220	9.065755	-0.006
H	2.354965	24.613672	7.681568	0.087
H	2.110993	23.176536	8.711139	0.051
H	3.317716	23.144501	7.399404	-0.010
H	-0.126387	24.652711	7.158372	-0.035
H	-0.384155	23.200070	8.165297	-0.005
H	-0.931459	23.223074	6.471260	-0.005
H	1.581278	24.597053	5.268345	-0.034
H	2.529474	23.123409	4.950350	-0.007
H	0.791950	23.161049	4.576436	0.016
H	-0.564375	18.407812	11.060453	-3.202
H	0.043139	18.808551	13.443088	0.585
H	2.243545	19.929631	13.974960	-4.876
H	3.730849	20.588021	12.040828	0.477
H	2.975714	20.109296	9.717807	-3.168
H	1.704413	20.932209	3.489765	-1.192
H	1.342206	21.194318	1.031429	0.229
H	0.757205	19.156643	-0.334332	-1.610
H	0.574669	16.943014	0.859515	0.129
H	0.979607	16.848867	3.304436	-1.074

Table S13. Cartesian coordinates of optimized closed-shell **3** (B3LYP/TZ2P) in Å

Atom	X	Y	Z
Ti	0.530809	18.945525	8.092824
Ti	3.546020	17.004804	7.502183
Ti	0.642243	15.438222	8.706043
Ti	-1.196082	16.903292	6.657735
Ti	1.795163	18.809414	5.826942
Ti	1.739282	15.217124	5.726466
O	2.387424	18.471821	7.737882
O	2.416682	16.938385	5.596251
O	2.314041	15.609935	7.787333

O	0.034195	15.510480	6.564741
O	-0.108442	17.132624	8.537064
O	-0.048765	18.377886	6.266413
O	-2.545350	15.658904	7.658375
C	-2.361029	14.878232	8.628980
O	-1.246112	14.690172	9.183623
C	-3.561908	14.054442	9.129503
C	-3.565107	12.751877	8.299695
C	-4.874035	14.816052	8.899482
C	-3.402005	13.716584	10.617966
O	-2.140504	16.766545	5.128062
C	-3.412879	17.029130	4.566303
C	-4.119098	15.712714	4.254936
C	-3.257269	17.916693	3.335237
O	-2.616565	18.281294	7.383150
C	-2.491007	19.286504	8.131265
O	-1.387678	19.686647	8.591636
C	-3.769619	20.081522	8.458581
C	-4.201922	20.795802	7.160940
C	-3.514915	21.118961	9.557327
C	-4.872929	19.106607	8.903329
O	1.189844	15.430514	10.398379
C	1.291144	15.267259	11.793076
C	1.501646	13.792838	12.126635
C	0.058502	15.852277	12.477974
O	0.857104	13.370397	8.427834
C	1.188395	12.703387	7.414966
O	1.523904	13.207497	6.308168
C	1.201687	11.165414	7.520661
C	0.328893	10.587208	6.391775
C	0.672004	10.700914	8.881355
C	2.657429	10.694723	7.339087
O	3.634809	14.439075	5.350198
C	4.752211	14.731838	5.852397
O	4.913870	15.612152	6.738330
C	5.984801	13.985655	5.310359
C	6.197248	14.465361	3.859127
C	7.230845	14.291925	6.148184
C	5.706751	12.473265	5.314721
O	1.147692	14.836840	4.057730
C	0.535957	13.770332	3.350132
C	1.612227	12.924386	2.676261
C	-0.471226	14.329801	2.351786

O	4.942253	18.226357	6.534724
C	4.801273	19.141988	5.677241
O	3.687935	19.508644	5.221441
C	6.082340	19.835540	5.179467
C	6.808023	20.436249	6.398569
C	5.757020	20.939456	4.168118
C	6.978164	18.768862	4.521637
O	4.465731	17.032134	9.058631
C	5.634440	16.511836	9.669021
C	5.248167	15.474391	10.718052
C	6.455806	17.657979	10.251406
O	0.833214	21.013903	7.934865
C	1.185911	21.563281	6.854298
O	1.504187	20.905035	5.826399
C	1.229754	23.097725	6.818619
C	2.246670	23.562648	7.880325
C	-0.170645	23.628814	7.179431
C	1.645827	23.610654	5.436115
N	1.093290	19.392017	10.287706
C	0.264158	20.102353	11.066926
C	0.568570	20.435763	12.376863
C	1.781224	20.021992	12.910922
C	2.641845	19.289691	12.107054
C	2.265248	18.995633	10.804409
N	1.316366	19.035972	3.614825
C	1.645776	20.160183	2.958595
C	1.438463	20.321176	1.599068
C	0.869563	19.280477	0.877468
C	0.532762	18.116409	1.551589
C	0.770092	18.031188	2.915095
H	-4.400263	12.120716	8.609052
H	-3.673773	12.964635	7.235795
H	-2.641264	12.192353	8.447505
H	-5.715423	14.206850	9.235035
H	-4.890388	15.752531	9.458333
H	-5.015410	15.053945	7.847360
H	-4.243775	13.105168	10.948388
H	-3.384089	14.621445	11.227572
H	-2.480698	13.168254	10.802264
H	-3.996776	17.571336	5.318261
H	-5.126596	15.901053	3.878334
H	-4.191968	15.103546	5.154848
H	-3.568626	15.149185	3.499836

H	-4.236146	18.172420	2.924662
H	-2.737694	18.839017	3.593597
H	-2.683308	17.403395	2.561971
H	-5.116164	21.364566	7.339988
H	-3.433409	21.491062	6.819230
H	-4.392778	20.077146	6.365152
H	-4.429953	21.682042	9.749888
H	-2.733846	21.820885	9.269583
H	-3.214158	20.640860	10.490484
H	-5.797894	19.655135	9.090432
H	-4.594647	18.593344	9.825648
H	-5.060413	18.353007	8.141591
H	2.174768	15.830272	12.116680
H	1.627143	13.661035	13.203154
H	2.389959	13.411706	11.624141
H	0.643345	13.205767	11.798333
H	0.157737	15.780948	13.562907
H	-0.066931	16.900288	12.208170
H	-0.835410	15.308881	12.170962
H	0.341601	9.496666	6.438152
H	-0.707936	10.915055	6.486812
H	0.696757	10.896690	5.415339
H	0.693325	9.610400	8.929667
H	-0.353717	11.030873	9.042695
H	1.276144	11.093519	9.697942
H	2.700708	9.604705	7.380886
H	3.298250	11.086875	8.130933
H	3.056842	11.022714	6.381460
H	7.053447	13.947162	3.423319
H	6.397195	15.537141	3.823811
H	5.318904	14.259232	3.249147
H	8.091546	13.767016	5.729520
H	7.449996	15.358235	6.160864
H	7.104323	13.964981	7.180617
H	6.560704	11.941542	4.891046
H	5.549999	12.103345	6.329100
H	4.822745	12.236999	4.726110
H	0.001252	13.148525	4.075767
H	1.160329	12.077954	2.154837
H	2.165410	13.523742	1.950631
H	2.314283	12.546472	3.417277
H	-1.010049	13.517476	1.860307
H	-1.192522	14.967769	2.860554

H	0.039437	14.913187	1.582906
H	7.736288	20.912748	6.077932
H	6.193864	21.192880	6.890346
H	7.047122	19.664488	7.127971
H	6.681208	21.413915	3.832614
H	5.119511	21.705851	4.608232
H	5.241926	20.538883	3.295674
H	7.912084	19.224924	4.187892
H	7.211667	17.971278	5.224761
H	6.489442	18.327954	3.651025
H	6.221018	16.019205	8.886956
H	6.141569	15.020608	11.151569
H	4.672219	15.936819	11.521945
H	4.639836	14.690236	10.269442
H	7.388128	17.281623	10.676887
H	5.899995	18.168551	11.040697
H	6.696618	18.384685	9.476365
H	2.284824	24.653190	7.899396
H	1.966648	23.206485	8.870200
H	3.248929	23.194549	7.654630
H	-0.156775	24.719915	7.199365
H	-0.484136	23.265631	8.156391
H	-0.912770	23.315666	6.442951
H	1.673140	24.701757	5.442784
H	2.634437	23.246292	5.158699
H	0.942533	23.293051	4.666627
H	-0.666415	20.403425	10.609448
H	-0.137309	21.009988	12.960744
H	2.049024	20.266617	13.930592
H	3.599231	18.946987	12.473789
H	2.921272	18.436299	10.153420
H	2.086796	20.942540	3.556244
H	1.723220	21.248457	1.121542
H	0.695058	19.374621	-0.186453
H	0.090963	17.275724	1.036683
H	0.531881	17.128664	3.457631

Table S14. Cartesian coordinates of optimized broken symmetry **3** (B3LYP /TZ2P) in Å

Atom	X	Y	Z
Ti	0.460401	18.902132	8.348231
Ti	3.481599	17.067923	7.512518
Ti	0.645157	15.392865	8.738973

Ti	-1.162645	16.928185	6.664649
Ti	1.748780	18.744119	5.640337
Ti	1.763169	15.163794	5.778479
O	2.351776	18.499240	7.656170
O	2.464197	16.837534	5.589388
O	2.294366	15.600763	7.842161
O	0.038550	15.512387	6.607575
O	-0.126928	17.115165	8.606889
O	0.003294	18.400761	6.329960
O	-2.537196	15.713543	7.647566
C	-2.375700	14.893642	8.592563
O	-1.273397	14.661675	9.150328
C	-3.615171	14.104865	9.055323
C	-3.975311	13.125406	7.918922
C	-4.781937	15.079917	9.283255
C	-3.323998	13.324491	10.341805
O	-2.051832	16.843237	5.105920
C	-3.189068	17.329814	4.418126
C	-4.142936	16.174966	4.127249
C	-2.747826	18.058785	3.152894
O	-2.571119	18.340903	7.385163
C	-2.522859	19.269292	8.235106
O	-1.476625	19.608922	8.853278
C	-3.820408	20.061913	8.490842
C	-4.008131	21.005474	7.283760
C	-3.717718	20.888910	9.778004
C	-5.013663	19.098304	8.581397
O	1.152914	15.400131	10.437988
C	1.426900	15.217629	11.806349
C	1.851136	13.774598	12.061909
C	0.210172	15.624697	12.633783
O	0.855505	13.338189	8.496161
C	1.144777	12.654506	7.477570
O	1.466766	13.145479	6.364951
C	1.110135	11.117864	7.593605
C	0.148949	10.572281	6.521144
C	0.643407	10.677554	8.985291
C	2.531670	10.589055	7.325835
O	3.643573	14.337625	5.505681
C	4.755850	14.700640	5.982373
O	4.899116	15.648194	6.793359
C	6.003439	13.942511	5.489896
C	6.222584	14.351054	4.017992

C	7.237783	14.311291	6.319895
C	5.748424	12.428574	5.570128
O	1.207048	14.765583	4.097315
C	0.654509	13.690699	3.355703
C	1.669304	13.221138	2.317278
C	-0.668462	14.123190	2.733363
O	4.901702	18.258681	6.515095
C	4.756036	19.112143	5.602439
O	3.640166	19.426006	5.102788
C	6.024969	19.816619	5.089491
C	6.630379	20.605250	6.267623
C	5.700884	20.769864	3.934363
C	7.023545	18.742068	4.622096
O	4.406176	17.149714	9.076918
C	5.617510	16.673121	9.637113
C	5.332952	15.487691	10.554159
C	6.326563	17.813136	10.362203
O	0.756133	20.903497	7.876453
C	1.089937	21.477079	6.798327
O	1.397762	20.860385	5.747660
C	1.132486	23.015429	6.812505
C	2.253105	23.434406	7.786185
C	-0.218587	23.546972	7.322949
C	1.421833	23.576263	5.416421
N	1.098460	19.411485	10.462949
C	0.232758	19.955011	11.331699
C	0.599524	20.324377	12.615585
C	1.913133	20.127712	13.019689
C	2.809314	19.565564	12.122740
C	2.367233	19.220405	10.853831
N	1.288083	18.889908	3.420917
C	1.111456	20.081577	2.830564
C	0.926154	20.216397	1.464571
C	0.924870	19.076054	0.672593
C	1.107248	17.843437	1.280924
C	1.286147	17.788743	2.655614
H	-4.860113	12.549274	8.196001
H	-4.185686	13.660700	6.994063
H	-3.161183	12.423561	7.731224
H	-5.678920	14.523299	9.561276
H	-4.557081	15.778292	10.091490
H	-4.992142	15.657099	8.385463
H	-4.212246	12.763782	10.639343

H	-3.055846	13.994896	11.158627
H	-2.501965	12.624061	10.205341
H	-3.692187	18.043108	5.080370
H	-5.054520	16.545004	3.653490
H	-4.413415	15.667728	5.052554
H	-3.675979	15.450058	3.458683
H	-3.612655	18.477112	2.633964
H	-2.066138	18.871705	3.401426
H	-2.236592	17.372223	2.476387
H	-4.921473	21.589690	7.410748
H	-3.171646	21.699906	7.193829
H	-4.087642	20.440411	6.355580
H	-4.640354	21.452906	9.927311
H	-2.888117	21.592507	9.733799
H	-3.572536	20.247562	10.648675
H	-5.936962	19.665396	8.714297
H	-4.909705	18.420557	9.429886
H	-5.100797	18.495181	7.680330
H	2.262930	15.881764	12.056156
H	2.109178	13.636343	13.113696
H	2.718546	13.519236	11.454731
H	1.039478	13.092518	11.806795
H	0.429824	15.539456	13.699764
H	-0.071514	16.654771	12.417546
H	-0.637267	14.979937	12.398271
H	0.116308	9.482571	6.576002
H	-0.865207	10.946940	6.670867
H	0.474428	10.858588	5.522681
H	0.624307	9.587137	9.037223
H	-0.356467	11.049666	9.205602
H	1.311116	11.046375	9.762714
H	2.532719	9.497988	7.363802
H	3.233969	10.951841	8.078428
H	2.887001	10.904975	6.346890
H	7.091230	13.826209	3.615852
H	6.405363	15.422997	3.930615
H	5.353962	14.099775	3.410659
H	8.109040	13.774161	5.940091
H	7.443397	15.379273	6.273080
H	7.104696	14.042604	7.368215
H	6.613345	11.888046	5.180931
H	5.588949	12.109314	6.601310
H	4.871666	12.148448	4.990031

H	0.462537	12.868908	4.052727
H	1.280797	12.364195	1.763081
H	1.888460	14.020466	1.605936
H	2.599924	12.929499	2.802725
H	-1.145209	13.279637	2.229860
H	-1.339910	14.501510	3.502250
H	-0.509068	14.914860	1.998072
H	7.547026	21.103206	5.946131
H	5.939693	21.370526	6.626340
H	6.867704	19.942228	7.097999
H	6.616260	21.257330	3.593149
H	4.994697	21.540286	4.241078
H	5.263039	20.236488	3.090937
H	7.949978	19.216292	4.292862
H	7.253191	18.047792	5.428145
H	6.622000	18.171576	3.782626
H	6.252514	16.331977	8.813671
H	6.264661	15.078755	10.950542
H	4.706175	15.794273	11.393643
H	4.812193	14.703404	10.006435
H	7.297197	17.482838	10.737360
H	5.732920	18.157696	11.211811
H	6.484473	18.653734	9.687067
H	2.305860	24.523256	7.841248
H	2.065750	23.044939	8.785694
H	3.224233	23.066212	7.451060
H	-0.188764	24.636482	7.383266
H	-0.446902	23.148245	8.309445
H	-1.030212	23.271096	6.647049
H	1.471442	24.665842	5.460593
H	2.368334	23.204519	5.026503
H	0.636703	23.304002	4.710248
H	-0.774189	20.093470	10.964771
H	-0.134924	20.758820	13.279295
H	2.231383	20.407497	14.015612
H	3.841727	19.393855	12.392130
H	3.036927	18.774824	10.130351
H	1.129586	20.938092	3.488756
H	0.789412	21.199610	1.036367
H	0.785439	19.148344	-0.398304
H	1.114810	16.928101	0.706129
H	1.431757	16.843142	3.160489

Table S15. Cartesian coordinates (Å) of optimized triplet **3** (B3LYP /TZ2P).

Atom	X	Y	Z
Ti	0.457733	18.896691	8.354923
Ti	3.485673	17.088480	7.521200
Ti	0.652085	15.396399	8.739904
Ti	-1.159986	16.921185	6.660962
Ti	1.734677	18.737451	5.625175
Ti	1.780518	15.168564	5.783786
O	2.346148	18.512344	7.648206
O	2.477499	16.842858	5.593057
O	2.302369	15.614654	7.848764
O	0.050917	15.512201	6.608001
O	-0.135213	17.113299	8.607127
O	-0.002755	18.403218	6.331190
O	-2.527511	15.699112	7.637630
C	-2.365386	14.878293	8.582209
O	-1.265024	14.652566	9.145446
C	-3.608711	14.096793	9.046549
C	-4.093569	13.243816	7.857372
C	-4.704406	15.104143	9.437459
C	-3.279609	13.191322	10.237974
O	-2.040509	16.838315	5.097473
C	-3.160257	17.339488	4.391593
C	-4.114500	16.193603	4.068156
C	-2.688369	18.078577	3.143456
O	-2.574798	18.327282	7.384929
C	-2.528295	19.255294	8.235318
O	-1.483005	19.598143	8.853558
C	-3.823841	20.051158	8.491864
C	-3.942770	21.075529	7.342817
C	-3.755623	20.791954	9.833284
C	-5.039296	19.112750	8.473825
O	1.154302	15.405732	10.440156
C	1.440086	15.219902	11.805706
C	1.894626	13.783801	12.048551
C	0.220561	15.597154	12.642838
O	0.872556	13.343979	8.501887
C	1.153287	12.659568	7.481192
O	1.482170	13.149881	6.370708
C	1.074184	11.123969	7.586556
C	-0.057684	10.652074	6.652147
C	0.777746	10.681899	9.023688
C	2.411807	10.525942	7.117072

O	3.663007	14.345608	5.528747
C	4.770671	14.708585	6.016895
O	4.908175	15.665108	6.817818
C	6.018991	13.932922	5.554462
C	6.234506	14.270627	4.064549
C	7.255365	14.340239	6.362886
C	5.763331	12.424310	5.708138
O	1.234824	14.764854	4.100502
C	0.694296	13.686992	3.354686
C	1.697934	13.257998	2.288195
C	-0.652533	14.093784	2.766842
O	4.895214	18.276455	6.513541
C	4.746380	19.114855	5.586216
O	3.630969	19.418102	5.081483
C	6.014596	19.812697	5.062312
C	6.625533	20.613398	6.229271
C	5.687533	20.753743	3.898071
C	7.009824	18.731466	4.602822
O	4.404925	17.185436	9.087577
C	5.624048	16.729771	9.648985
C	5.356632	15.546647	10.574037
C	6.317802	17.884656	10.365327
O	0.759511	20.897364	7.873602
C	1.079099	21.469119	6.790249
O	1.373198	20.851837	5.735673
C	1.122069	23.007771	6.802000
C	2.257585	23.427037	7.758226
C	-0.220411	23.541062	7.332433
C	1.390443	23.566946	5.401087
N	1.087264	19.401743	10.470042
C	0.202144	19.873409	11.360695
C	0.561055	20.226238	12.651341
C	1.887906	20.092287	13.037701
C	2.804488	19.606126	12.117469
C	2.368147	19.268478	10.844550
N	1.283404	18.873953	3.404422
C	1.068997	20.060706	2.816940
C	0.884613	20.193109	1.450541
C	0.927893	19.055876	0.655273
C	1.150636	17.828414	1.260686
C	1.322881	17.775660	2.636296
H	-4.988797	12.688450	8.143088
H	-4.331585	13.870343	6.999414

H	-3.333087	12.522618	7.553233
H	-5.610265	14.569407	9.729360
H	-4.389802	15.717466	10.284091
H	-4.940941	15.765294	8.606248
H	-4.173677	12.641454	10.538415
H	-2.932307	13.771518	11.092569
H	-2.500289	12.472729	9.988568
H	-3.672340	18.048827	5.051031
H	-5.012772	16.573501	3.577143
H	-4.409543	15.678852	4.981796
H	-3.636189	15.472368	3.403700
H	-3.539427	18.508386	2.611266
H	-2.006215	18.883843	3.414296
H	-2.168360	17.394948	2.470704
H	-4.849066	21.670320	7.471602
H	-3.089339	21.754503	7.332832
H	-3.996213	20.574415	6.376469
H	-4.674406	21.361237	9.986335
H	-2.914322	21.481985	9.863542
H	-3.652412	20.093203	10.665200
H	-5.954633	19.691843	8.610267
H	-4.982021	18.378167	9.278098
H	-5.105850	18.572386	7.532132
H	2.264451	15.898940	12.055120
H	2.158825	13.642516	13.098375
H	2.765353	13.551808	11.436808
H	1.097050	13.086770	11.789657
H	0.447859	15.508082	13.706907
H	-0.081641	16.623385	12.436048
H	-0.615671	14.938027	12.407269
H	-0.141224	9.564589	6.695236
H	-1.018245	11.075603	6.950215
H	0.139014	10.942660	5.621379
H	0.718785	9.592550	9.067171
H	-0.165648	11.093271	9.379592
H	1.559760	11.009256	9.708435
H	2.356861	9.435853	7.138036
H	3.230535	10.833366	7.769884
H	2.648157	10.844950	6.103897
H	7.102739	13.727930	3.685928
H	6.416437	15.337429	3.925697
H	5.364637	13.990555	3.472022
H	8.126338	13.788409	6.003959

H	7.459094	15.405552	6.267946
H	7.126738	14.118326	7.422537
H	6.625686	11.864691	5.340771
H	5.609849	12.155358	6.754653
H	4.882662	12.117700	5.147684
H	0.539186	12.850787	4.043785
H	1.320036	12.397956	1.731483
H	1.879867	14.071493	1.582391
H	2.646601	12.985046	2.748920
H	-1.117103	13.246339	2.258481
H	-1.317953	14.441448	3.555231
H	-0.528910	14.901914	2.042805
H	7.541883	21.106152	5.899027
H	5.937377	21.383862	6.581732
H	6.864704	19.959354	7.066180
H	6.602092	21.237020	3.548779
H	4.982733	21.527780	4.198842
H	5.246559	20.211667	3.061882
H	7.935777	19.200524	4.264879
H	7.241406	18.045630	5.415577
H	6.604101	18.152304	3.771282
H	6.262394	16.392095	8.826891
H	6.293552	15.152021	10.972685
H	4.725765	15.850255	11.411628
H	4.846115	14.752381	10.031070
H	7.293689	17.571030	10.741276
H	5.720313	18.225669	11.213598
H	6.462716	18.722762	9.684267
H	2.313244	24.515928	7.809854
H	2.083956	23.040197	8.761206
H	3.223036	23.056075	7.409951
H	-0.187290	24.630371	7.394956
H	-0.435916	23.140373	8.321078
H	-1.041756	23.268852	6.666946
H	1.440587	24.656613	5.443114
H	2.330873	23.194620	4.997343
H	0.594726	23.293793	4.707156
H	-0.814062	19.968230	11.005543
H	-0.189238	20.600344	13.333644
H	2.200581	20.362443	14.038024
H	3.847299	19.483258	12.372975
H	3.051489	18.874262	10.104493
H	1.056677	20.915445	3.477724

H	0.715311	21.172187	1.024539
H	0.790939	19.126517	-0.416052
H	1.192469	16.915667	0.683211
H	1.493840	16.834098	3.140809

Table S16. Cartesian coordinates of optimized **5** (PBE /TZ2P) in Å

Atom	X	Y	Z
C	6.889918	11.742618	15.740926
C	5.660653	10.873204	15.408791
C	10.362576	12.227136	12.993927
C	10.320432	11.570010	11.600347
C	13.919993	14.365213	14.339767
C	15.120815	14.424796	13.373716
C	14.873262	13.362864	12.281426
C	15.173614	15.822039	12.729097
C	16.435620	14.129146	14.104188
C	14.003560	16.142419	18.401920
C	15.287871	16.868564	18.851486
C	14.998409	18.316994	19.260229
C	16.296798	16.839184	17.687733
C	15.862504	16.090147	20.053400
C	10.509459	15.475018	21.025175
C	6.989334	13.570499	19.649207
C	5.711052	13.580727	20.513824
C	5.339957	15.051745	20.790666
C	4.580901	12.908207	19.710290
C	5.935687	12.835931	21.834230
O	11.281006	11.615236	19.633793
O	9.982226	11.254264	20.188688
O	9.238378	12.725494	17.620039
O	9.255950	15.487006	17.787851
O	11.536519	14.063985	18.495148
O	11.703425	15.451275	16.340772
O	11.570214	12.928586	15.824973
O	9.252364	13.918445	15.378502
O	6.711141	12.927636	16.151829
O	8.030217	11.195908	15.609128
O	10.089744	11.486850	13.988010
O	10.667501	13.457563	13.073287
O	12.794140	14.729062	13.886908
O	14.120972	13.935718	15.519498

O	12.884021	16.731921	18.521437
O	14.144472	14.964849	17.950281
O	10.737697	16.268331	20.062761
O	10.214492	14.244860	20.890059
O	6.913953	14.166918	18.523029
O	8.017688	12.995575	20.101164
O	7.057210	15.627249	16.058069
O	10.341428	16.163649	13.976035
O	10.531010	10.401702	16.640514
O	13.592056	12.192094	17.727894
Ti	9.997073	12.905096	19.373598
Ti	11.015183	15.819858	18.021855
Ti	12.797561	13.626102	17.071523
Ti	10.876462	14.648579	14.758343
Ti	9.919245	11.936581	16.014809
Ti	8.089648	14.365857	16.855582
C	8.900370	11.012503	11.380198
C	11.336668	10.409976	11.595021
C	10.666275	12.579854	10.500311
C	4.379063	11.713816	15.375078
C	5.558603	9.804146	16.518966
C	5.878915	10.183033	14.050738
C	14.317241	11.182414	18.408333
C	15.508173	10.759039	17.551036
C	14.722085	11.680790	19.792545
C	9.642262	8.420240	17.671655
C	10.898303	9.209999	17.313144
C	11.871718	8.422251	16.439219
C	5.929258	15.826841	15.223336
C	4.974055	16.814180	15.891079
C	6.388465	16.292182	13.842818
C	10.289547	17.402637	13.300463
C	11.539461	18.224581	13.612811
C	10.104639	17.156386	11.803669
C	9.251759	18.344091	17.064102
C	8.816013	19.647129	16.835511
C	9.612457	20.711756	17.252118
C	10.822074	20.430305	17.884493
C	11.192616	19.101135	18.070023
N	10.426144	18.069639	17.666942
C	10.609510	16.043251	22.453394
C	12.082571	16.442047	22.683578
C	10.175991	15.005911	23.495300

C	9.716088	17.295270	22.543126
H	15.693709	13.386970	11.549799
H	13.928874	13.556380	11.756899
H	14.831697	12.352545	12.712843
H	16.004017	15.866305	12.009908
H	15.338397	16.602942	13.485427
H	14.239397	16.045001	12.199281
H	17.270205	14.165877	13.389156
H	16.627192	14.866020	14.895300
H	16.420298	13.135863	14.569881
H	15.928627	18.799395	19.592885
H	14.273778	18.364493	20.083529
H	14.599705	18.897420	18.416779
H	17.239100	17.310951	18.001344
H	16.503625	15.807905	17.377464
H	15.914216	17.390818	16.816740
H	4.413626	15.093834	21.381637
H	6.130101	15.559323	21.362617
H	5.185528	15.601226	19.853664
H	3.644293	12.946946	20.285134
H	4.426630	13.416166	18.750116
H	4.811776	11.851874	19.511288
H	5.012391	12.857522	22.431262
H	6.740338	13.296702	22.421488
H	6.213743	11.788766	21.660458
H	8.852616	10.497143	10.410081
H	8.154376	11.820183	11.372299
H	8.631244	10.301892	12.170891
H	11.303482	9.895413	10.623924
H	12.360789	10.778867	11.749331
H	11.107088	9.685079	12.385746
H	10.626311	12.083654	9.519892
H	11.673771	12.992897	10.638062
H	9.960328	13.420396	10.492031
H	3.518698	11.063846	15.159951
H	4.203617	12.216387	16.334129
H	4.427477	12.484449	14.593559
H	4.705257	9.141716	16.314060
H	5.402156	10.267224	17.503246
H	6.471347	9.196585	16.563585
H	5.027276	9.523214	13.830927
H	6.796499	9.582822	14.058671
H	5.956935	10.919033	13.237901

H	13.627378	10.328525	18.526798
H	16.048112	9.933975	18.036333
H	16.200883	11.601520	17.416741
H	15.173856	10.422481	16.561109
H	15.247073	10.884542	20.338914
H	15.391863	12.547639	19.705070
H	13.831322	11.969392	20.363503
H	11.398971	9.524747	18.245241
H	12.746238	9.035262	16.185569
H	12.215503	7.523988	16.971087
H	11.382876	8.110177	15.505456
H	5.419908	14.850802	15.120810
H	4.066545	16.941691	15.283856
H	5.454610	17.797059	16.006480
H	4.681739	16.452445	16.885179
H	5.529299	16.380067	13.162392
H	6.877824	17.275032	13.911106
H	7.105599	15.578480	13.417875
H	9.401995	17.940742	13.684889
H	11.471080	19.214488	13.139625
H	11.652202	18.359030	14.696041
H	12.434321	17.712789	13.233673
H	10.016591	18.112111	11.267798
H	9.197336	16.567099	11.619432
H	10.965058	16.603598	11.402187
H	8.647411	17.483887	16.772414
H	7.858119	19.813309	16.342798
H	9.294359	21.742797	17.091340
H	11.478541	21.226827	18.234343
H	12.129606	18.827322	18.553769
H	12.191836	16.888096	23.682559
H	12.411159	17.173210	21.933767
H	12.743096	15.564979	22.628993
H	10.260383	15.438805	24.502500
H	10.801104	14.105596	23.449824
H	9.135104	14.693547	23.339472
H	9.807831	17.742301	23.543323
H	10.008689	18.042107	21.794903
H	8.658809	17.040883	22.381307
H	8.982633	9.026879	18.304113
H	9.916595	7.512538	18.227344
H	9.100556	8.125582	16.761639
H	15.161998	16.091839	20.900556

H	16.799447	16.560743	20.384545
H	16.071906	15.048669	19.779500

Table S17. B3LYP/TZ2P energies for compound **2**

	Hartree	eV
Closed-shell	-43.435989	0.99
Triplet	-43.472442	0.00
Broken symmetry	-43.472416	0.00

Table S18. Cartesian coordinates (Å) of optimized closed-shell configuration **2** of B3LYP/TZ2P

Atom	X	Y	Z
C	6.781411	0.822731	2.205291
C	6.124153	2.076387	1.636838
C	5.794531	-0.330168	2.359544
C	14.275130	4.283072	4.887659
C	14.886561	4.514942	6.258254
C	15.279332	3.843091	3.834827
C	10.501483	-1.261158	3.569054
C	10.788280	-2.411673	2.589356
C	9.452507	-3.037323	2.149416
C	11.501052	-1.802745	1.364569
C	11.680705	-3.475945	3.236886
C	10.256345	6.319181	4.733369
C	10.483281	7.791736	4.352033
C	11.005440	7.869073	2.907139
C	11.550770	8.347619	5.318043
C	9.190292	8.602867	4.487370
C	10.577158	2.876646	1.920075
C	10.899129	3.140480	0.438298

C	12.370437	2.755303	0.191755
C	9.985042	2.322936	-0.479881
C	10.708691	4.644907	0.166475
O	9.092483	3.203623	4.719476
O	10.820489	3.848742	6.670072
O	11.096457	1.553036	4.870720
O	6.853212	4.335761	6.023417
O	7.374910	1.111646	3.462084
O	13.200220	3.307069	4.971914
O	9.677664	-0.378333	3.197360
O	11.105364	-1.237761	4.671559
O	9.169958	5.988310	5.276757
O	11.198677	5.506019	4.496594
O	11.289937	3.485815	2.776638
O	9.648847	2.091391	2.219353
Ti	9.031035	1.343198	4.155797
Ti	11.030472	3.445024	4.829758
Ti	8.654129	4.220380	6.332174
C	6.007008	4.766239	4.968740
C	4.772021	3.872786	4.928386
C	5.646272	6.235778	5.169947
H	7.582754	0.512050	1.527381
H	6.853539	2.880847	1.554735
H	5.309479	2.409894	2.280825
H	5.717309	1.873854	0.643920
H	6.291477	-1.203168	2.781881
H	4.971181	-0.047204	3.017441
H	5.378358	-0.606380	1.388590
H	13.762185	5.183892	4.555674
H	14.114122	4.755356	6.986128
H	15.598859	5.340564	6.214044

H	15.421558	3.627576	6.602356
H	14.788080	3.685604	2.875814
H	16.050490	4.604647	3.710223
H	15.772462	2.914077	4.131458
H	9.639999	-3.835904	1.429516
H	8.805969	-2.295313	1.684999
H	8.920779	-3.470352	2.998680
H	11.704546	-2.584702	0.630869
H	10.883380	-1.038343	0.895333
H	12.453095	-1.351142	1.648484
H	11.877187	-4.274844	2.519490
H	11.203776	-3.914033	4.113361
H	12.633085	-3.055877	3.556084
H	11.207342	8.908907	2.644172
H	11.922607	7.295376	2.789190
H	10.269695	7.480992	2.201079
H	11.752209	9.393372	5.079560
H	12.482674	7.789579	5.236211
H	11.210223	8.297233	6.353349
H	9.383710	9.645806	4.229846
H	8.800684	8.564004	5.503091
H	8.415190	8.227033	3.819457
H	12.633716	2.953130	-0.848834
H	13.035931	3.330260	0.833322
H	12.538636	1.694542	0.385526
H	10.239394	2.522353	-1.522562
H	10.093824	1.254201	-0.298935
H	8.937206	2.579270	-0.328644
H	10.961921	4.867116	-0.871764
H	11.345332	5.242435	0.816250
H	9.672374	4.946259	0.330300

H	6.549362	4.658230	4.021889
H	4.106147	4.168597	4.115357
H	4.219692	3.953131	5.867582
H	5.062690	2.834571	4.772560
H	5.020173	6.591213	4.348734
H	6.547509	6.844235	5.212260
H	5.097644	6.364901	6.104876
H	13.516385	2.422524	5.207151
C	12.928409	3.594711	10.308369
C	13.586606	2.340892	10.875388
C	13.914544	4.748418	10.155270
C	5.433846	0.138959	7.627832
C	4.825246	-0.100040	6.257173
C	4.427783	0.585895	8.675930
C	9.209051	5.679145	8.944002
C	8.925985	6.832669	9.921222
C	10.263746	7.455758	10.358791
C	8.212376	6.228485	11.147845
C	8.036034	7.897839	9.271762
C	9.450906	-1.901502	7.778123
C	9.221658	-3.374782	8.155277
C	8.694856	-3.455341	9.598297
C	8.156758	-3.927337	7.184476
C	10.514388	-4.186694	8.022049
C	9.133814	1.540320	10.593276
C	8.813994	1.273099	12.074895
C	7.340754	1.649609	12.323012
C	9.724151	2.094294	12.993681
C	9.013136	-0.230759	12.344248
O	10.617893	1.214100	7.793748
O	8.890074	0.569455	5.843155

O	8.614320	2.864906	7.643156
O	12.856358	0.079654	6.491886
O	12.335254	3.306588	9.051255
O	6.510011	1.113245	7.540609
O	10.032331	4.796061	9.316197
O	8.606026	5.656101	7.840978
O	10.537598	-1.570912	7.235084
O	8.509112	-1.087931	8.015470
O	8.421167	0.931120	9.736515
O	10.061715	2.326072	10.294198
Ti	10.679090	3.074638	8.357354
Ti	8.679692	0.973107	7.683681
Ti	11.056226	0.197201	6.181358
C	13.701877	-0.350048	7.547427
C	14.936411	0.543986	7.588693
C	14.063309	-1.819536	7.347117
H	12.126877	3.904160	10.986615
H	12.857764	1.535876	10.956807
H	14.401386	2.008578	10.230894
H	13.993515	2.542686	11.868431
H	13.417068	5.621412	9.733543
H	14.738270	4.466578	9.497344
H	14.330326	5.024135	11.126535
H	5.944885	-0.760614	7.966045
H	5.599059	-0.345556	5.532511
H	4.111820	-0.924528	6.304502
H	4.292264	0.785998	5.906687
H	4.917086	0.747943	9.635205
H	3.655398	-0.174013	8.802937
H	3.936499	1.513906	8.373176
H	10.078954	8.256573	11.076910

H	10.908590	6.713096	10.824550
H	10.796059	7.885286	9.508100
H	8.011451	7.012703	11.879839
H	8.828258	5.463491	11.618445
H	7.258942	5.778814	10.865544
H	7.842169	8.698937	9.987413
H	8.513613	8.332597	8.393977
H	7.082319	7.479612	8.954127
H	8.491249	-4.495671	9.857993
H	7.777826	-2.881153	9.714869
H	9.428718	-3.069683	10.307647
H	7.953569	-4.973469	7.419791
H	7.225118	-3.368600	7.264660
H	8.500687	-3.874764	6.150411
H	10.319287	-5.230099	8.276409
H	10.907284	-4.145667	7.007683
H	11.287640	-3.813169	8.693407
H	7.079297	1.448797	13.363490
H	6.678060	1.071963	11.680990
H	7.166502	2.709711	12.130932
H	9.471326	1.892269	14.036232
H	9.609562	3.162676	12.814110
H	10.773259	1.843758	12.841647
H	8.762098	-0.456032	13.382358
H	8.379170	-0.830782	11.694125
H	10.050972	-0.526030	12.178988
H	13.158584	-0.241875	8.493701
H	15.601666	0.248700	8.402422
H	15.489611	0.463683	6.650007
H	14.645080	1.582083	7.744018
H	14.688768	-2.174444	8.169049

H	13.162326	-2.428344	7.304197
H	14.612837	-1.948830	6.412746
H	6.195084	1.997664	7.303068

Table S19. Cartesian coordinates (Å) of optimized triplet configuration **2** of B3LYP/TZ2P

Atom	X	Y	Z
C	6.818955	0.873062	2.226198
C	6.228528	2.066716	1.481749
C	5.768118	-0.176024	2.576412
C	14.462638	4.209421	4.734982
C	15.291641	4.677800	5.919015
C	15.264244	3.458962	3.685333
C	10.475460	-1.231996	3.561482
C	10.695871	-2.415519	2.601270
C	9.344813	-3.133080	2.415191
C	11.171920	-1.855151	1.248228
C	11.735537	-3.393687	3.157887
C	10.302734	6.354639	4.827376
C	10.434824	7.842531	4.447423
C	10.953834	7.948562	3.002962
C	11.462177	8.470118	5.411527
C	9.093058	8.571269	4.575164
C	10.807565	2.920760	2.012583
C	11.129037	3.202585	0.533009
C	12.647357	3.095794	0.311642
C	10.396487	2.221989	-0.389020
C	10.667632	4.644588	0.237081
O	9.162206	3.233770	4.789749
O	10.763094	3.904351	6.759605

O	11.126690	1.506614	4.859273
O	6.839801	4.318996	5.948499
O	7.458548	1.316196	3.409833
O	13.361833	3.375579	5.202781
O	9.667120	-0.337327	3.196161
O	11.103968	-1.219276	4.654061
O	9.214805	5.952860	5.324247
O	11.310532	5.618630	4.637409
O	11.506358	3.516515	2.882603
O	9.858053	2.145617	2.287439
Ti	9.101074	1.449034	4.143004
Ti	11.240353	3.525002	4.950165
Ti	8.600036	4.208305	6.361195
C	5.963947	4.724717	4.909032
C	4.753647	3.796861	4.889223
C	5.572830	6.185629	5.110331
H	7.582727	0.407682	1.594326
H	7.005809	2.799160	1.267843
H	5.453530	2.547859	2.080249
H	5.784938	1.744559	0.537604
H	6.225431	-1.004413	3.116772
H	4.987388	0.258093	3.203383
H	5.303254	-0.568005	1.669666
H	13.953507	5.057731	4.280832
H	14.661825	5.174518	6.655044
H	16.056964	5.379825	5.584474
H	15.793458	3.836616	6.401693
H	14.619539	3.131710	2.871802
H	16.042450	4.104180	3.275565
H	15.752634	2.583648	4.120845
H	9.463760	-3.967698	1.721980

H	8.593535	-2.454182	2.015334
H	8.976676	-3.532833	3.361697
H	11.301980	-2.671949	0.536043
H	10.448876	-1.150056	0.842563
H	12.130384	-1.343060	1.350535
H	11.872761	-4.219769	2.457555
H	11.422193	-3.804591	4.116565
H	12.698518	-2.906856	3.307546
H	11.093575	8.997875	2.736393
H	11.904328	7.430590	2.889654
H	10.243501	7.514988	2.297114
H	11.598387	9.525843	5.169880
H	12.426348	7.969521	5.334229
H	11.123045	8.402675	6.446536
H	9.221699	9.625207	4.321072
H	8.700221	8.505381	5.588372
H	8.347648	8.148681	3.901516
H	12.883496	3.311331	-0.731910
H	13.184234	3.803482	0.940209
H	13.009870	2.091230	0.536838
H	10.637560	2.447966	-1.429421
H	10.691004	1.192689	-0.186086
H	9.317237	2.288715	-0.264105
H	10.892670	4.897480	-0.800634
H	11.175152	5.357165	0.885615
H	9.591535	4.751191	0.383758
H	6.499846	4.623625	3.959056
H	4.070830	4.070721	4.082915
H	4.208714	3.867709	5.833547
H	5.071089	2.766121	4.731754
H	4.932265	6.523819	4.293338

H	6.461373	6.813437	5.141461
H	5.030574	6.307162	6.049817
H	13.665929	2.496372	5.472635
C	12.884680	3.551150	10.283099
C	13.486713	2.359893	11.022101
C	13.926133	4.609306	9.932286
C	5.240509	0.199886	7.767134
C	4.395154	-0.224741	6.578218
C	4.455500	0.921032	8.849341
C	9.226402	5.653712	8.950339
C	9.007079	6.838520	9.909225
C	10.359758	7.552018	10.099509
C	8.524886	6.280368	11.260934
C	7.972299	7.819627	9.348658
C	9.404203	-1.935275	7.683327
C	9.273606	-3.422649	8.066066
C	8.778159	-3.523774	9.519480
C	8.228386	-4.049013	7.121024
C	10.610596	-4.156277	7.917732
C	8.900672	1.495842	10.500352
C	8.599650	1.196928	11.980823
C	7.079567	1.186889	12.209287
C	9.259942	2.231589	12.898910
C	9.173008	-0.205321	12.275952
O	10.541172	1.188315	7.721870
O	8.938596	0.517549	5.752009
O	8.575141	2.914287	7.652129
O	12.864648	0.105876	6.560319
O	12.244429	3.106464	9.100458
O	6.341341	1.041635	7.313741
O	10.034584	4.758967	9.315977

O	8.599778	5.641779	7.856695
O	10.491794	-1.532706	7.186193
O	8.396545	-1.199671	7.874929
O	8.200883	0.900615	9.630991
O	9.846366	2.275445	10.224336
Ti	10.601143	2.972843	8.369171
Ti	8.463591	0.895098	7.562431
Ti	11.103714	0.213593	6.150465
C	13.741225	-0.296927	7.600374
C	14.950878	0.631630	7.618031
C	14.133327	-1.758039	7.402089
H	12.119514	4.008857	10.918960
H	12.715817	1.620928	11.236663
H	14.263163	1.886388	10.419420
H	13.931311	2.682557	11.965586
H	13.460461	5.435706	9.396106
H	14.707738	4.183104	9.301007
H	14.391332	5.002043	10.838542
H	5.749706	-0.665397	8.188043
H	5.012941	-0.701605	5.819255
H	3.628529	-0.931845	6.898707
H	3.893829	0.634991	6.128763
H	5.111349	1.217173	9.665812
H	3.677198	0.267962	9.246357
H	3.968643	1.813200	8.447465
H	10.241252	8.387222	10.792077
H	11.107636	6.870930	10.502093
H	10.732263	7.950356	9.154070
H	8.394569	7.098140	11.971959
H	9.244596	5.573635	11.669509
H	7.565363	5.770862	11.155623

H	7.835364	8.646409	10.048212
H	8.290164	8.229256	8.390918
H	7.008349	7.335722	9.195946
H	8.637142	-4.571821	9.790318
H	7.832445	-3.000402	9.647740
H	9.502858	-3.093207	10.212582
H	8.093514	-5.103817	7.367299
H	7.267188	-3.545502	7.213446
H	8.550072	-3.984757	6.080279
H	10.482411	-5.209512	8.174933
H	10.987507	-4.092675	6.898300
H	11.368225	-3.735599	8.578769
H	6.865832	0.952635	13.253638
H	6.594154	0.442030	11.582012
H	6.640650	2.161453	11.988012
H	9.040229	1.990033	13.940542
H	8.886801	3.235327	12.696155
H	10.340353	2.246565	12.769376
H	8.972663	-0.473714	13.314827
H	8.718306	-0.956232	11.631037
H	10.253133	-0.228395	12.123946
H	13.205707	-0.194365	8.550430
H	15.634391	0.358948	8.424147
H	15.495336	0.560001	6.673548
H	14.633255	1.662407	7.775202
H	14.774189	-2.094198	8.219663
H	13.245024	-2.386337	7.372485
H	14.675272	-1.881477	6.462680
H	6.036087	1.923852	7.055271

Table S20. Cartesian coordinates (Å) of optimized broken symmetry configuration **2** of B3LYP/TZ2P

Atom	X	Y	Z
C	6.816844	0.879538	2.223771
C	6.223522	2.078298	1.490119
C	5.768601	-0.174873	2.564906
C	14.462109	4.226930	4.742030
C	15.276674	4.693231	5.936941
C	15.279249	3.490184	3.694558
C	10.473872	-1.235326	3.564298
C	10.693676	-2.420011	2.605409
C	9.340951	-3.132741	2.412871
C	11.177958	-1.861212	1.254583
C	11.727385	-3.401995	3.166553
C	10.296541	6.352777	4.822061
C	10.425168	7.840547	4.440790
C	10.961759	7.948371	3.002958
C	11.436503	8.474591	5.417532
C	9.077813	8.562034	4.550780
C	10.806799	2.914535	2.011045
C	11.128577	3.193353	0.531051
C	12.646729	3.082015	0.310421
C	10.393420	2.213426	-0.389715
C	10.671290	4.636297	0.232973
O	9.161173	3.230289	4.789428
O	10.764139	3.902520	6.757343
O	11.127368	1.504311	4.860088
O	6.838888	4.318161	5.950599
O	7.455981	1.313840	3.411133
O	13.363689	3.381388	5.193506
O	9.664914	-0.341368	3.198575

O	11.101922	-1.221967	4.657057
O	9.210404	5.949328	5.321058
O	11.306231	5.619044	4.632785
O	11.504679	3.513993	2.879331
O	9.857941	2.139583	2.288222
Ti	9.098859	1.445872	4.143671
Ti	11.241547	3.524859	4.946746
Ti	8.599259	4.205410	6.361093
C	5.958030	4.734271	4.919089
C	4.745856	3.809169	4.898762
C	5.571263	6.194123	5.134390
H	7.581820	0.421622	1.587673
H	6.999158	2.814131	1.281647
H	5.448303	2.552770	2.093683
H	5.779410	1.763556	0.543631
H	6.227771	-1.006886	3.098130
H	4.986806	0.252235	3.195430
H	5.304616	-0.560088	1.654738
H	13.950460	5.074541	4.289227
H	14.636365	5.179849	6.670663
H	16.039830	5.403247	5.614399
H	15.780311	3.852263	6.418263
H	14.644610	3.162635	2.873135
H	16.055535	4.144647	3.295857
H	15.771293	2.616145	4.128809
H	9.459957	-3.967536	1.719789
H	8.593907	-2.450920	2.009895
H	8.966961	-3.531632	3.357541
H	11.308663	-2.678493	0.542990
H	10.459059	-1.153749	0.845577
H	12.137651	-1.352210	1.361364

H	11.864636	-4.228643	2.466818
H	11.408337	-3.811795	4.123895
H	12.691482	-2.918629	3.320478
H	11.098946	8.998258	2.737056
H	11.916464	7.435527	2.901797
H	10.262777	7.510111	2.288549
H	11.570002	9.530859	5.176470
H	12.404390	7.979259	5.353147
H	11.084286	8.406197	6.448077
H	9.203163	9.615934	4.294691
H	8.673602	8.497142	5.559618
H	8.342681	8.132997	3.869758
H	12.883576	3.294084	-0.733739
H	13.185221	3.790092	0.937229
H	13.006620	2.077129	0.538585
H	10.634665	2.437582	-1.430528
H	10.685635	1.183637	-0.185695
H	9.314307	2.282836	-0.264559
H	10.898093	4.887719	-0.804801
H	11.180039	5.348295	0.881427
H	9.595254	4.746041	0.378384
H	6.488934	4.639567	3.965565
H	4.060260	4.088960	4.096736
H	4.204835	3.875589	5.845711
H	5.060558	2.778638	4.734238
H	4.924958	6.539726	4.324941
H	6.461074	6.820242	5.163433
H	5.036285	6.309257	6.078906
H	13.672518	2.504949	5.466726
C	12.890656	3.545820	10.281203
C	13.487160	2.350947	11.018557

C	13.936559	4.600406	9.933593
C	5.239202	0.193030	7.757466
C	4.400187	-0.218623	6.559601
C	4.448073	0.899965	8.844747
C	9.230996	5.654171	8.947293
C	9.012076	6.839292	9.905805
C	10.365872	7.548920	10.101743
C	8.523410	6.281160	11.255372
C	7.981806	7.823672	9.342505
C	9.407676	-1.935333	7.687858
C	9.278144	-3.423240	8.068749
C	8.774471	-3.528081	9.518867
C	8.239948	-4.050493	7.116245
C	10.617642	-4.153567	7.927062
C	8.906093	1.498700	10.501615
C	8.603038	1.206426	11.982895
C	7.082767	1.215165	12.211377
C	9.275509	2.235653	12.898351
C	9.159645	-0.201707	12.281385
O	10.542869	1.188116	7.722331
O	8.938815	0.516950	5.754253
O	8.576004	2.914025	7.652018
O	12.866028	0.102813	6.561671
O	12.249653	3.105915	9.096805
O	6.341214	1.041266	7.320158
O	10.039152	4.759609	9.313348
O	8.603064	5.641007	7.854462
O	10.493919	-1.531675	7.189240
O	8.398600	-1.201191	7.878859
O	8.204403	0.902446	9.634392
O	9.851415	2.277560	10.222634

Ti	10.605268	2.972865	8.367721
Ti	8.462840	0.893341	7.565542
Ti	11.105141	0.213190	6.150919
C	13.744643	-0.310766	7.596324
C	14.955755	0.615727	7.617756
C	14.133600	-1.770513	7.384321
H	12.126293	4.005295	10.916955
H	12.713127	1.614717	11.231389
H	14.262209	1.875257	10.415770
H	13.932269	2.670298	11.963034
H	13.474883	5.429799	9.398496
H	14.717188	4.172194	9.302407
H	14.402415	4.989564	10.841114
H	5.747567	-0.676633	8.170539
H	5.022110	-0.686453	5.798392
H	3.632515	-0.929833	6.868447
H	3.900271	0.645808	6.117471
H	5.100181	1.188178	9.667202
H	3.669552	0.240718	9.231125
H	3.961027	1.795891	8.451390
H	10.246886	8.383788	10.794740
H	11.110391	6.865371	10.506361
H	10.743069	7.947215	9.158134
H	8.392117	7.098622	11.966675
H	9.240152	5.572566	11.666308
H	7.563198	5.773643	11.146082
H	7.844784	8.650458	10.042122
H	8.303937	8.233001	8.385979
H	7.016980	7.342491	9.186282
H	8.634069	-4.577091	9.786474
H	7.827083	-3.006759	9.642809

H	9.494333	-3.097625	10.217015
H	8.105628	-5.106087	7.359804
H	7.276990	-3.549214	7.203791
H	8.567723	-3.983803	6.077473
H	10.490740	-5.207182	8.183597
H	10.999473	-4.089285	6.909453
H	11.370929	-3.731127	8.592005
H	6.866096	0.986588	13.256436
H	6.588591	0.474070	11.586252
H	6.655278	2.194176	11.987006
H	9.053045	1.999509	13.940697
H	8.914209	3.243331	12.693016
H	10.356100	2.237696	12.768897
H	8.956374	-0.464998	13.321066
H	8.695999	-0.948541	11.638336
H	10.239465	-0.237983	12.129316
H	13.211220	-0.214820	8.548274
H	15.640771	0.337324	8.420762
H	15.497911	0.549327	6.671478
H	14.639618	1.646000	7.781349
H	14.777546	-2.114393	8.196384
H	13.244544	-2.397555	7.352960
H	14.671830	-1.886288	6.441750
H	6.035824	1.922183	7.057285

References

1. Ziegenbalg, D.; Pannwitz, A.; Rau, S.; Dietzek-Ivanšić, B.; Streb, C., *Angew. Chem. Int. Ed.* **2022,** *61* (28), e202114106.
2. Tauc, J.; Menth, A.; Wood, D. L., *Phys. Rev. Lett.* **1970,** *25* (11), 749-752.
3. Makuła, P.; Pacia, M.; Macyk, W., *J. Phys. Chem. Lett.* **2018,** *9* (23), 6814-6817.
4. Benedict, J. B.; Coppens, P., *J. Am. Chem. Soc.* **2010,** *132* (9), 2938-2944.

5. te Velde, G.; Bickelhaupt, F. M.; Baerends, E. J.; Fonseca Guerra, C.; van Gisbergen, S. J. A.; Snijders, J. G.; Ziegler, T., *J. Comput. Chem.* **2001,** *22* (9), 931-967.
6. *ADF 2021.1, SCM, Theoretical Chemistry, Vrije Universiteit, Amsterdam, The Netherlands*, http://www.scm.com.
7. Van Lenthe, E.; Baerends, E. J., *J. Comput. Chem.* **2003,** *24* (9), 1142-1156.
8. Perdew, J. P.; Burke, K.; Ernzerhof, M., *Phys. Rev. Lett.* **1996,** *77* (18), 3865-3868.
9. Lenthe, E. v.; Baerends, E. J.; Snijders, J. G., *J. Chem. Phys.* **1993,** *99* (6), 4597-4610.
10. Lenthe, E. v.; Baerends, E. J.; Snijders, J. G., *J. Chem. Phys.* **1994,** *101* (11), 9783-9792.
11. Lenthe, E. v.; Ehlers, A.; Baerends, E.-J., *J. Chem. Phys.* **1999,** *110* (18), 8943-8953.
12. van Gisbergen, S. J. A.; Snijders, J. G.; Baerends, E. J., *Comput. Phys. Commun.* **1999,** *118* (2), 119-138.
13. Rosa, A.; Baerends, E. J.; van Gisbergen, S. J. A.; van Lenthe, E.; Groeneveld, J. A.; Snijders, J. G., *J. Am. Chem. Soc.* **1999,** *121* (44), 10356-10365.
14. Lenthe, E. v.; Avoird, A. v. d.; Wormer, P. E. S., *J. Chem. Phys.* **1998,** *108* (12), 4783-4796.
15. Piszczek, P.; Grodzicki, A.; Richert, M.; Wojtczak, A., *Inorg. Chim. Acta* **2004,** *357* (9), 2769-2775.
16. Boyle, T. J.; Tyner, R. P.; Alam, T. M.; Scott, B. L.; Ziller, J. W.; Potter, B. G., *J. Am. Chem. Soc.* **1999,** *121* (51), 12104-12112.
17. Piszczek, P.; Radtke, A.; Muzioł, T.; Richert, M.; Chojnacki, J., *Dalton Trans.* **2012,** *41* (27), 8261-8269.
18. Radtke, A.; Piszczek, P.; Muzioł, T.; Wojtczak, A., *Inorg. Chem.* **2014,** *53* (20), 10803-10810.
19. Brown, I. D., Bond valence parameters https://www.iucr.org/resources/data/datasets/bond-valence-parameters. IUCr, Ed. 2020.
20. Krämer, T.; Tuna, F.; Pike, S. D., *Chem. Sci.* **2019,** *10* (28), 6886-6898.
21. Rajca, A.; Olankitwanit, A.; Rajca, S., *J. Am. Chem. Soc.* **2011,** *133* (13), 4750-4753.
22. Bader, R. F. W., *Chem. Rev.* **1991,** *91* (5), 893-928.
23. Bader, R. F. W., *Atoms in Molecules*. Clarendon Press: 1994.
